# Design, Synthesis, and Trypanosomicidal Evaluation of Eugenol-Based Azole Hybrids: Discovery of an *In Vitro* Active and Selective Compound

**DOI:** 10.1021/acsomega.5c06619

**Published:** 2025-08-29

**Authors:** José Vaz Cardoso Machado, Clara Oliveira de Carvalho Lopes, Sarah Ferreira Maciel, Valquíria Ângelis Fernandes, Sara Manuela Mendonça da Silva Cravo, Maria Emília da Silva Pereira de Sousa, Maria Elizabeth Tiritan, Thiago Belarmino de Souza, Lucas Lopardi Franco, Lívia de Figueiredo Diniz, Diogo Teixeira Carvalho

**Affiliations:** † Faculdade de Ciências Farmacêuticas, 74347 Universidade Federal de Alfenas, Alfenas, MG 37130-001, Brazil; ‡ Faculdade de Farmácia, 131671 Universidade do Porto, Porto 4099-002, Portugal; § Escola de Farmácia, 28115 Universidade Federal de Ouro Preto, Ouro Preto, MG 35400-000, Brazil; ∥ Instituto de Ciências Biomédicas, Universidade Federal de Alfenas, Alfenas, MG 37130-001, Brazil

## Abstract

Chagas disease, caused by *Trypanosoma cruzi*, remains a major public health concern in Latin America and beyond, with limited treatment options and high morbidity in chronic stages. Currently, benznidazole remains the first-line therapy for Chagas disease but exhibits considerable limitations, including dose-dependent toxicity and poor efficacy in long-term infections. In response to the pressing need for safer and more effective therapies, this study reports the design, synthesis, and biological evaluation of 17 novel eugenol-based hybrid compounds (**18**–**34**), strategically constructed by integrating pharmacophoric features from nitroaromatic trypanosomicidal agents, azole-based CYP51 inhibitors, and the phenylpropanoid core of eugenol. The synthetic sequence included key steps such as nitration, *O*-alkylation, epoxidation, nucleophilic epoxide opening, and CuAAC reactions to afford 1,2,4-triazole-, imidazole-, and 1,2,3-triazole-containing hybrids. All compounds were structurally characterized by IR, HRMS, and NMR spectroscopy. Biological assays were performed against both trypomastigote and amastigote forms of *T. cruzi* (Y strain), revealing compound-dependent antiparasitic profiles. Hybrid **29**, bearing benzyl and nitro substituents on an imidazole ring, emerged as the most active candidate (EC_50_ = 26.5 μmol·L^–1^) with a selectivity index exceeding 49. Enantioseparation by chiral HPLC enabled the isolation of **(+)-29** and **(−)-29** enantiomers, both of which showed comparable activity, indicating no significant stereoselectivity. Cytotoxicity assays in mammalian cell lines (Vero and H9c2) confirmed low toxicity for most hybrids (CC_50_ > 1300 μmol·L^–1^). Although the compounds were inactive against intracellular amastigotes, the observed trypomastigote-selective activity—particularly of nitrobenzylated derivatives suggests a non-CYP51-related mechanism of action. These findings highlight the value of phenylpropanoid-based molecular hybridization as a promising strategy for developing new antiparasitic agents against *T. cruzi*.

## Graphical abstract

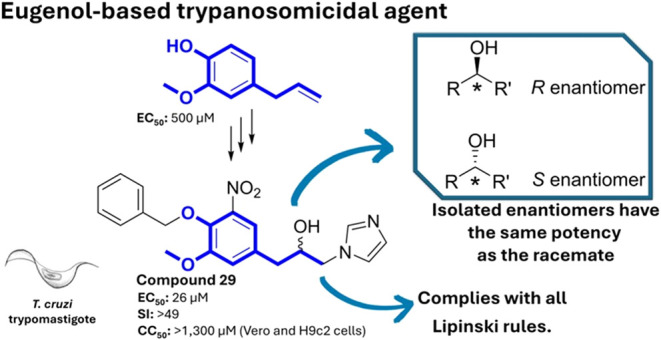

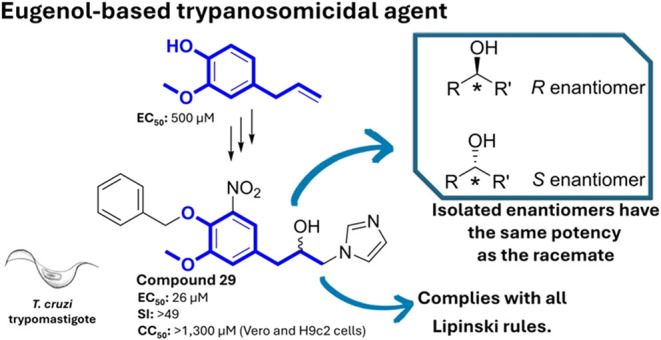

## 1 Introduction

The exploration of natural products as sources of new therapeutic agents has become a central strategy in medicinal chemistry, especially in the search for treatments against neglected tropical diseases.^1^ Among the many bioactive natural scaffolds, eugenol—a phenylpropanoid and the principal active constituent of clove oil (*Syzygium aromaticum*)—has attracted considerable interest due to its broad-spectrum antimicrobial and antiparasitic activities,^2–7^ making it a promising starting point for the development of novel antiparasitic compounds. Leveraging its chemical versatility,^8^ our research has focused on the design and synthesis of phenylpropanoid-based derivatives evaluated against diverse biological targets, including *Trypanosoma cruzi*, the etiological agent of Chagas disease.

Chagas disease, endemic to Latin America, affects 6 to 7 million people globally and remains a major public health concern.^9–11^ The disease is associated with substantial morbidity and mortality, especially during its chronic phase.^12^ Over 30% of chronically infected individuals develop life-threatening cardiac complications, including arrhythmias, cardiomyopathy, heart failure, and sudden cardiac death, while a smaller proportion experiences debilitating gastrointestinal manifestations such as megacolon and megaesophagus.^13,14^ The insidious nature of its progression, often asymptomatic for decades, masks its true public health impact, resulting in disability-adjusted life years (DALYs) and approximately 10,000 deaths annually.^15–17^ Currently, benznidazole (Figure 1) continues to be the first-line therapy, despite established limitations. Its parasitological cure rates reach 80–90% in acute or early congenital infections; however, in chronic cases—particularly in adults—its efficacy drops significantly, with cure rates ranging from 5% to 40% depending on diagnostic criteria and follow-up duration.^18^ Clinical trials like BENEFIT and STOP-CHAGAS showed no significant effect on cardiac progression,^19,20^ and adverse effects—including dermatological, gastrointestinal, and neurological symptoms—led to treatment discontinuation in 10–20% of patients.^21^ These limitations underscore the urgent need for safer and more effective therapies.

**Figure 1:**
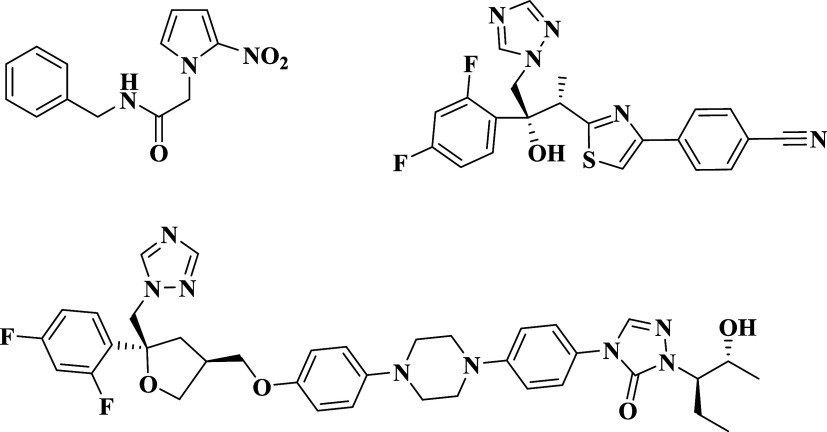
Structures of benznidazole- and azole-based CYP51 inhibitors investigated for Chagas disease.

A wide range of molecular targets has been explored for Chagas disease drug discovery, including Cruzipain, CPSF3, and ergosterol biosynthesis enzymes such as sterol 14α-demethylase (CYP51), each contributing to vital biological processes in *T. cruzi*.^22^ Among them, CYP51 has emerged as the most extensively validated target, particularly due to the ravuconazole and posaconazole *in vitro* and *in vivo* activities against *T. cruzi*. These azole-based agents demonstrate inhibitory effects at subnanomolar concentrations, thereby effectively disrupting the replication of intracellular amastigote forms of the parasite.^23–27^ Nonetheless, despite promising preclinical activity, recent clinical trials indicate that these compounds fail to deliver sustained efficacy upon treatment conclusion.^20,21,28,29^ Such findings suggest that, although azole-based CYP51 inhibitors provide a strong mechanistic foundation for drug development, combination therapy or structural optimization strategies may be necessary to overcome their current limitations.^30,31^

Our group’s ongoing studies have consistently yielded encouraging results in developing phenylpropanoid-based hybrid molecules with antimicrobial properties^32–35^ (Figure 2). Collectively, these studies underscore the valuable role of eugenol as a versatile scaffold in medicinal chemistry. Reinforcing this perspective, a comprehensive review by Reis and co-workers^36^ summarized the synthetic strategies and biological evaluations of a wide range of phenylpropanoid derivatives, demonstrating substantial antimicrobial and antiparasitic potential. For instance, Brancaglion and coauthors^32^ reported phenylpropanoid-based coumarin hybrids, with the nitrobenzoyl derivative—synthesized via Knoevenagel condensation of a formylated dihydroeugenol—showing an *in vitro* IC_50_ of 28 μmol L^–1^ against epimastigotes, along with low mammalian cytotoxicity and significant parasitaemia reduction in *in vivo* models. Nevertheless, this compound was not superior to benznidazole in reducing parasitaemia. In particular, Sierra and collaborators^37^ highlighted the potential of coumarins in antiprotozoal drug discovery, reinforcing the value of natural phenylpropanoids as starting points for medicinal chemistry efforts. Building upon the coumarin chemical framework, Oliveira and co-workers^33^ synthesized a eugenol-based chloramphenicol hybrid through reduction of a coumarin precursor, demonstrating potent *in vitro* antimicrobial activity, including effectiveness against multidrug-resistant bacterial strains and rapidly growing mycobacteria.

**Figure 2:**
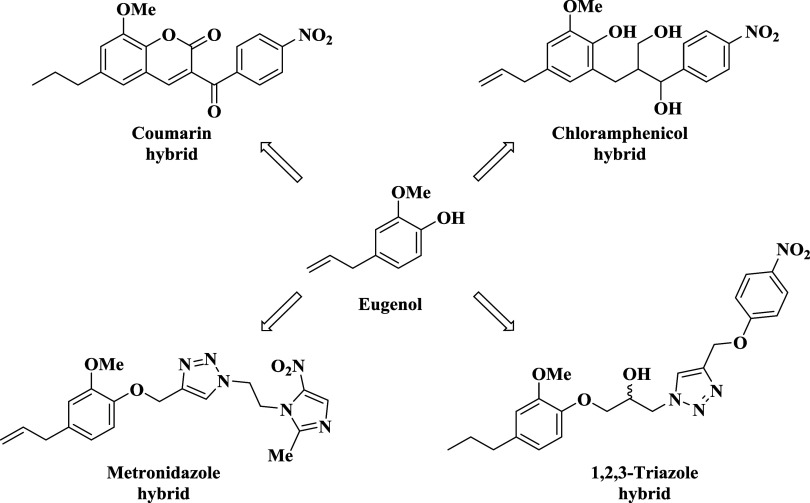
Antimicrobial compounds obtained from eugenol or dihydroeugenol were obtained by molecular hybridization.

Further advancing in this eugenol-base, Pelozo and colleagues^34^ prepared metronidazole hybrids through a 1,2,3-triazole linker via a copper­(I)-catalyzed azide–alkyne cycloaddition (CuAAC) reaction, which exhibited *in vitro* trypanosomicidal activity comparable to benznidazole against both epimastigote and trypomastigote forms of *T. cruzi*. Expanding on this work, Gonçalves-Santos and co-workers^38^ further reported the pharmacological potential of new nitroeugenol-based metronidazole hybrids, demonstrating significant *in vitro* antiparasitic effects by attenuating cellular parasitism and oxidative stress in infected cardiomyocytes. Moreover, induced relevant *in vivo* antiparasitic and cardioprotective effects, reducing parasitemia, cardiac parasite load, and myocarditis in *T. cruzi*-infected mice, while being well tolerated without causing mortality or hepatotoxicity. In line with these findings, Reis and co-workers^35^ designed and synthesized novel 1,2,3-triazole derivatives based on eugenol and its analogues, demonstrating *in vitro* activity against epimastigotes (IC_50_ of 7.3 μmol·L^–1^) and trypomastigotes (IC_50_ of 8.41 μmol·L^–1^), displaying a higher selectivity index (>77) compared to benznidazole. Critically, 100 mg/kg also demonstrated significant *in vivo* efficacy, achieving a 99.4% reduction in parasitemia peak and reducing myocarditis, positioning it as a new lead compound for Chagas disease treatment.

Building upon these previous studies, a series of novel eugenol-based azole hybrids with trypanosomicidal potential was synthesized and biologically evaluated. The rationale underlying the design of the novel structural motif was based on the hypothesis that the strategic combination of distinct pharmacophoric units—namely, residues derived from trypanosomicidal nitro-compounds (*e*.*g*., benznidazole), azole-based CYP51 inhibitors, and eugenol itself—could generate hybrid compounds with enhanced activity against *T. cruzi* (Figure 3). Seventeen hybrid compounds (**18**–**34**) were systematically evaluated for their antiparasitic activity against both extracellular (trypomastigote) and intracellular (amastigote) forms of *T. cruzi*. Furthermore, extensive cytotoxic evaluations in mammalian cell lines (Vero and H9c2) were conducted, focusing on both toxicity and selectivity—key parameters in drug discovery.

**Figure 3:**
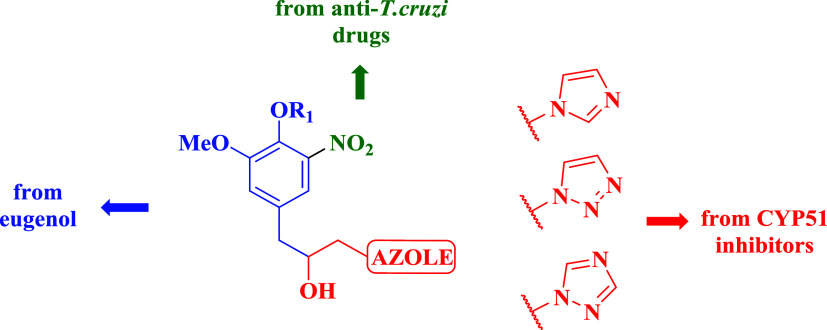
Rational approach for the design of novel molecular hybrids combining pharmacophoric units from benznidazole, azole CYP51 inhibitors, and eugenol derivatives, aiming for enhanced efficacy against *T. cruzi*.

## 2 Results and Discussion

The retrosynthetic analysis of hybrid series **A**, presented in Scheme 1, reveals a synthetic plan centered on the late-stage introduction of an azole moiety through an epoxide ring-opening, resulting in a secondary alcohol. To the best of our knowledge, these heterocyclic hybrid structures have been reported here for the first time. A strategic C–O bond disconnection was proposed using epoxide series **B**. The adopted strategy yields a racemic mixture of enantiomers, which is acceptable at this stage, as initial biological assays typically prioritize structural diversity over stereopurity. This synthetic design necessitates the late-stage installation of the epoxide, which can be efficiently formed from an allylic intermediate derived from precursor series **C** due to its nucleophilicity. The terminal disconnections, when applicable, involving the nitro functionality and *O*-alkylation step strategically led to eugenol, defined as the starting material for the synthesis.

**Scheme 1:**
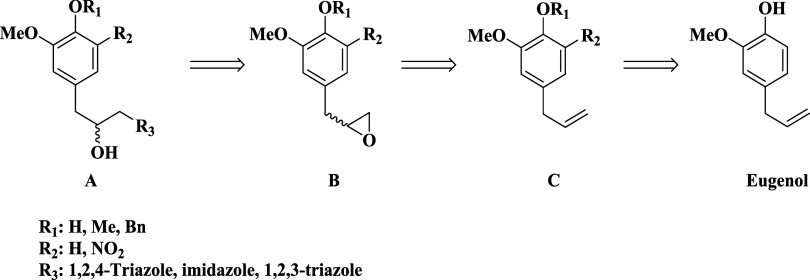
Retrosynthetic Analysis of Eugenol-Based Azole Hybrids

Based on the retrosynthetic analysis, the synthetic route employed to obtain the eugenol–azole hybrids **18–34** is illustrated in Scheme 2. The synthesis commenced with the *ortho*-nitration of eugenol, a transformation extensively explored in the literature for its regioselectivity and operational simplicity, as demonstrated in the functionalization of phenolic natural products and in the optimization of nitration protocols under mild conditions.^39,40^ Among the available methodologies, we selected a rapid protocol involving the dropwise addition of nitric acid to eugenol dissolved in glacial acetic acid. Although subsequent neutralization and purification steps were required, this approach afforded the desired nitroeugenol derivative in a satisfactory yield of 70%, which proved adequate for continuation of the synthetic sequence.^41^ An alternative method employing NaNO_3_ and KHSO_4_ demonstrated excellent yields (up to 90%) using an operationally simple and scalable protocol, although this method was not tested in the current study.^42^ Subsequently, *O*-alkylation of both eugenol and its nitro-derivative was carried out via phenoxide formation in a protic solvent, to methylation reaction, although in *O*-benzylation, DMF was used.^8,42^ No further purification was necessary, as unwanted byproducts were efficiently removed through basic aqueous washing, yielding the target compounds as clean oils upon organic extraction. Column chromatography was utilized solely to assess the product purity and determine isolated yields.

**Scheme 2:**
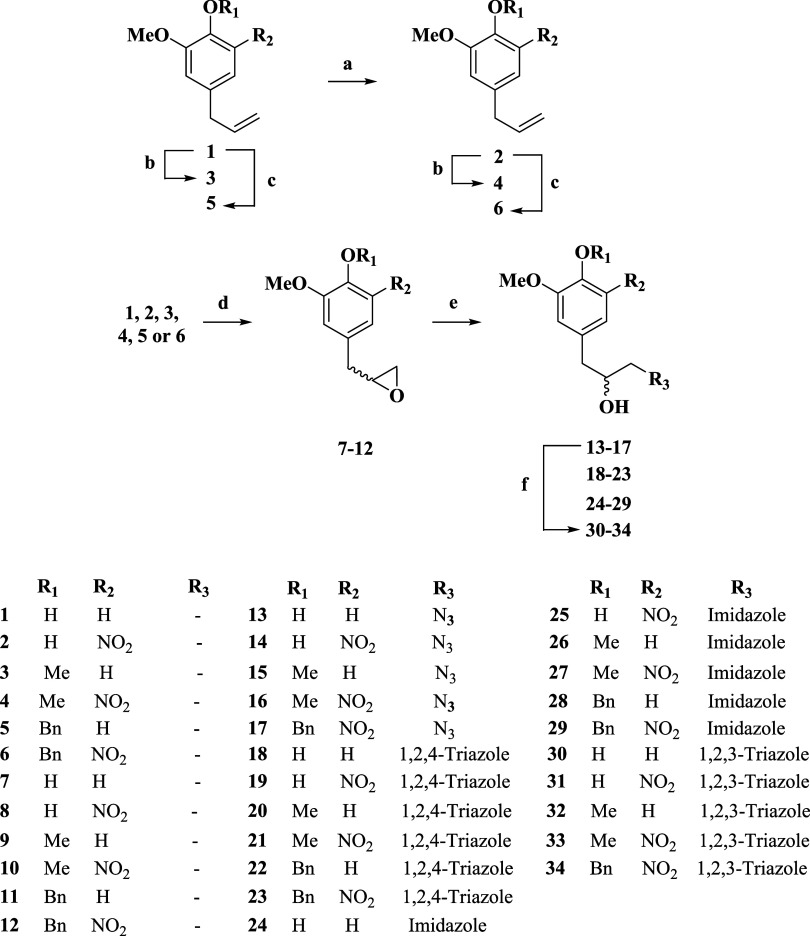
Synthetic Procedures from Eugenol (**1**) to Obtain Its Derivatives (**2**–**34**)­^a^

a Reagents and conditions: (a) Acetic acid, nitric acid, room temperature; (b) methyl iodide, K_2_CO_3_, EtOH, 70 °C; (c) benzyl chloride, K_2_CO_3_, DMF, 80 °C; (d) *m*-chloroperbenzoic acid, CHCl_3_, 0 °C to r.t.; (e) NaN_3_ or 1,2,4-triazole or imidazole, DMF, 80 °C; (f) K_2_CO_3_, ascorbic acid, Cu­(OAc)_2_, trimethylsilylacetylene, H_2_O/MeOH (1:1 v/v), r.t.

Thereafter, the allylic side chain of eugenol and its derivatives was oxidized using *m*-chloroperbenzoic acid (*m*-CPBA) as the peracid oxidant in a polar aprotic solvent, avoiding aqueous conditions.^43–45^ The epoxidation reactions herein provided moderate yields (∼50%), despite the presence of electron-donating groups in the eugenol derivatives, typically enhancing reactivity.^46^ Although a polar aprotic solvent was employed to moderate the reaction and minimize side reactions, the observed yields suggest significant steric hindrance or partial epoxide ring-opening.^43^

Following, the synthesized epoxides were subjected to nucleophilic ring-opening reactions (Step e). These transformations were carried out in DMF at elevated temperatures under vigorous stirring, employing 1,2,4-triazole, imidazole, or sodium azide as nucleophiles. This approach afforded 12 hybrid products (compounds **18**–**29**) through direct nucleophilic addition and five azide intermediates (compounds **13**–**17**), designed for subsequent CuAAC cycloaddition. The azide intermediates were then reacted with trimethylsilylacetylene via a CuAAC following a two-step, one-pot protocol, as described by Fletcher and colleagues.^47^ This strategy combines Cu-catalyzed cycloaddition with the subsequent *in situ* base-catalyzed deprotection of the TMS group, allowing efficient and regioselective formation of monosubstituted 1,2,3-triazole derivatives under mild conditions. During the *in situ* deprotection, the trimethylsilyl group is cleaved, generating volatile silanol derivatives that are removed during the evaporation of solvents, thus simplifying the isolation of the final products. The adoption of this one-pot protocol minimized purification steps and avoided the need for separate deprotection procedures, highlighting its operational simplicity and synthetic efficiency. This approach successfully afforded five additional hybrid compounds (compounds **30**–**34**). Structural characterization of all final products was supported by IR spectroscopy, which revealed O–H stretching bands between 3290 and 3456 cm^–1^, by ^1^H NMR, which showed characteristic multiplets corresponding to the 2-hydroxypropyl side chain at δ 2.0–2.5 and 4.0–4.5 ppm, and by ^13^C NMR, with distinct methylene signals observed around δ 40 and 52–55 ppm.^48,49^ Mass spectrometry data further corroborated the proposed structures.

The purity of the final eugenol-based azole hybrids was assessed by thin-layer chromatography (TLC) using multiple mobile phases of distinct polarity and a range of visualizing reagents in combination with ^1^H NMR spectra analysis. The TLC profiles consistently showed single spots under all tested conditions, with no detectable secondary spots indicative of residual starting materials or side products. Likewise, the ^1^H NMR spectra displayed well-resolved signals consistent with the proposed structures without resonances attributable to impurities. Taken together, these complementary analytical approaches provided sufficient evidence that the synthesized compounds were of adequate purity for initial exploratory biological evaluations.

With 17 eugenol-based azole hybrids (compounds **18–34**) successfully obtained and evaluated, Table 1 summarizes their biological evaluation, along with the enantiomerically resolved forms of the most active candidate, compound **29**. Following literature recommendations, the compounds were tested against both extracellular (trypomastigote) and intracellular (amastigote) forms of *T. cruzi* (Y strain) as well as for cytotoxicity in mammalian cell lines (Vero and H9c2). Benznidazole and eugenol were included as reference compounds. The selectivity index (SI) was calculated for the compounds that showed trypanosomicidal activity, providing insight into their therapeutic potential.^50–52^

**Table 1:**
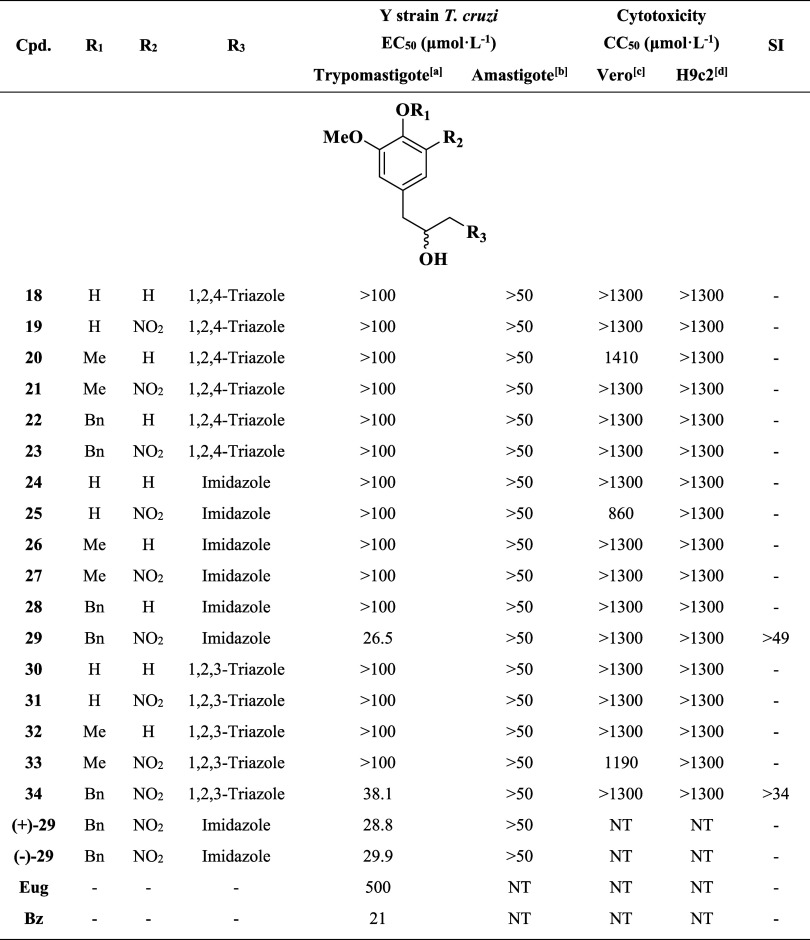
Anti-*T. cruzi* and Cytotoxic Activity of Hybrids (**18**–**34**)

a Determined after 24 h of incubation in the presence of compounds.

b Determined in Vero or H9c2 cells infected with amastigote forms of *T. cruzi* after 48 h of incubation.

c Determined in Vero cells by resazurin method for 48 h incubation.

d Determined in H9c2 cells by resazurin method for 48 h incubation. NT = not tested; **Eug** = eugenol; **Bz** = benznidazole; SI = selectivity index.

As presented, hybrids **29** and **34** exhibited significant trypanosomicidal activity against the trypomastigote form of *T. cruzi* (Y strain), with EC_50_ values of 26.5 and 38.1 μmol·L^–1^, respectively—markedly more potent than eugenol (EC_50_ =  500 μmol·L^–1^) roughly confirmed by literature,^2,34^ though less active than benznidazole (EC_50_ = 21 μmol·L^–1^) although it has high variable values.^53^ Both active compounds share a nitroaromatic substituent at the R_2_ position and a benzyl group at R_1_, suggesting that this combination may be critical for activity against trypomastigotes. The presence of the NO_2_ group is closely associated with the mechanism of action (MoA) of benznidazole,^54,55^ suggesting that the hybrids may act through a similar pathway and reinforcing the relevance of this new scaffold to potentially overcome pharmacokinetic and resistance-related limitations.^56,57^ Furthermore, the biological evaluation of hybrids **29** and **34** revealed that the incorporation of bulky hydrophobic substituents, such as a benzyl group, significantly enhanced activity against the trypomastigote forms of *T. cruzi*. These findings suggest that hydrophobic interactions play a crucial role in facilitating access to the molecular target. Supporting this, Pelozo and colleagues^34^ reported that the respective presence of allyl or propyl moiety in eugenol- and dihydroeugenol-based metronidazole hybrids was essential for *in vitro* activity against both epimastigote and trypomastigote forms. Brancaglion and coauthors^32^ demonstrated that a eugenol-based coumarin hybrid bearing a hydrophobic side chain exhibited superior *in vitro* activity against epimastigote forms of *T. cruzi* compared to a vanillin-based analogue lacking this moiety; moreover, in an acute *in vivo* assay—characterized by elevated levels of circulating trypomastigotes, the propyl-substituted compound was more effective than its allyl analogue, underscoring the critical role of lipophilic chain structure in modulating antiparasitic efficacy. Despite bearing a 1,2,3-triazole ring instead of the imidazole present in compound **29**, hybrid **34** still exhibited potent trypanosomicidal activity, albeit slightly less pronounced. In contrast, hybrid **23**—containing a 1,2,4-triazole ring—was inactive, an outcome consistent with reports that azole-based CYP51 inhibitors are generally less effective against trypomastigote forms of *T. cruzi*.^24^ Most other hybrids also lacked measurable activity against trypomastigote forms, supporting the critical role of the benzylic and nitro groups observed in active compounds **29** and **34**.

Given the prominent trypanosomicidal activity against trypomastigote forms observed for hybrid **29**, further investigation was undertaken to evaluate the influence of chirality on its biological performance. To this end, enantiomers **(+)-29** and **(−)-29** were isolated and individually evaluated. The enantiomeric resolution of compound **29** was performed by high-performance liquid chromatography (HPLC) using polysaccharide-based chiral stationary phases (CSPs), specifically derivatized amylose and cellulose columns.^58^ These CSPs are widely recognized for their high enantioselectivity, broad applicability, and compatibility with various elution modes.^59^ The enantioseparation experiments revealed superior performance on the cellulose-based chiral stationary phase (CSP), particularly when using *n*-hexane/EtOH (80:20) as the mobile phase (Figure 4), which yielded a retention factor (*k*_1_) of 5.25, a selectivity (α) of 1.3, and a resolution (*R*_s_) of 2.4—indicative of effective enantiomeric discrimination. In contrast, the amylose analytical column generally produced modest retention factors and low selectivity (α ≈ 1.0, 1.1), reflecting poor chiral discrimination under most tested conditions. These observations highlight the cellulose column as the most suitable option for analytical enantioseparation in this study. Subsequently, the enantiomers of compound **29** were isolated on a submilligram scale, guided by the retention time profile presented in Figure 4, allowing preparative-scale collection for subsequent biological evaluation, as previously demonstrated by Sousa and coauthors.^60^

**Figure 4:**
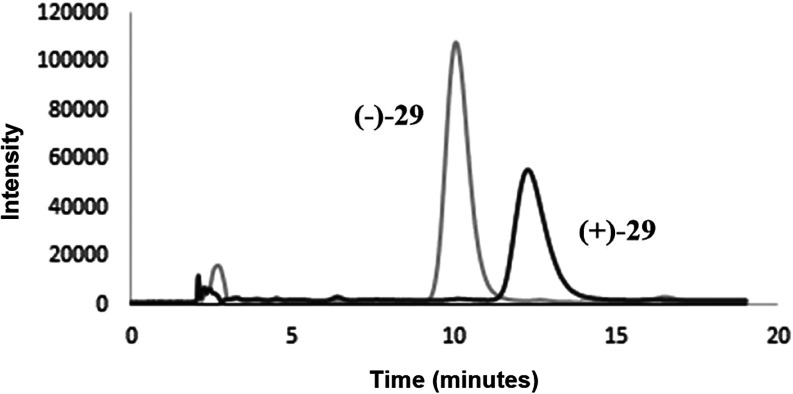
Chromatogram of isolated enantiomers of **29** under cellulose-based CSP. Chromatographic conditions: CSP: cellulose tris­(3,5-dimethylphenylcarbamate), mobile phase: *n*-hexane/ethanol (80:20, v/v), flow rate: 0.8 mL·min^–1^, detection at 260 nm.

Following these parameters, each enantiomer of compound **29** was successfully isolated, yielding high-purity fractions with enantiomeric excess (ee) values exceeding 99%, as confirmed by analytical HPLC analysis, presented in Table 2. The elution order of the enantiomers was determined based on their specific optical rotation values ([α]_D_), where the (−)-enantiomer eluted first, followed by the (+)-enantiomer. From an initial quantity of 2.00 mg of racemic compound **29**, a total of 0.77 mg of **(−)-29** and 0.73 mg of **(+)-29** were isolated under optimized chromatographic conditions. This separation corresponds to an overall recovery of approximately 75%, indicating good efficiency of the resolution process with minimal material loss.^58,60^

**Table 2:** Recovery, Enantiomeric Excess, and Specific Rotation for **29** Isolated Enantiomers

Hybrid	Elution order	[α]_D_ (c)	e.e. (%)	Recovery (%)	Mass (mg)
(+)-29	second	+111,11° (0.036)	>99	73	0.73
(−)-29	first	–42,55° (0.047)	>99	77	0.77

The obtention and subsequent re-evaluation of the isolated enantiomers **(+)-29** and **(−)-29**, as detailed in Figure 5, allowed for an in-depth analysis of their trypanosomicidal activity compared to the racemic mixture. As illustrated by the dose–response curves and corroborated by the EC_50_ values around 29 μmol·L^–1^ (Table 1), both enantiomers exhibited statistically identical activity profiles. The figure clearly demonstrates a dose dependence, where parasite mortality increases with compound concentration. At higher concentrations, notably at 100 μmol·L^–1^, the activity reached approximately 99% for all forms (racemic and enantiomeric), indicating a comparable maximum effect. At intermediate concentrations, such as 50 μmol·L^–1^, the activity remained substantial but not total, corroborating the reported EC_50_. The almost perfect superimposition of the curves for **(+)-29**, **(−)-29**, and the racemic mixture across all tested dilutions visually confirms that enantiomeric separation did not confer any significant advantage in potency or efficacy against *T. cruzi* trypomastigote forms in this *in vitro* assay.

**Figure 5:**
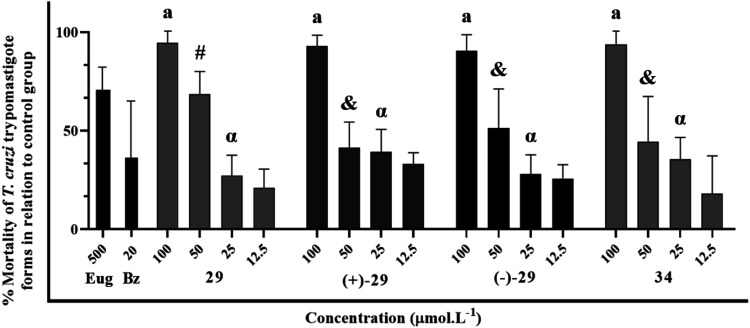
Dose–response curves of trypanosomicidal activity for racemic **29**, **(+)-29**, **(−)-29**, and **34** against *T. cruzi* Y strain trypomastigotes incubated for 24 h with serial dilution of 100 to 12.5 μmol L^–1^ of hybrids. Statistical analysis with *P* < 0.05.

*In vitro* assays were performed using host cells infected with intracellular amastigote forms of *T. cruzi* revealed that none of the synthesized hybrids exhibited measurable trypanosomicidal activity at the tested concentrations (Table 1). This lack of efficacy is particularly notable, given that some of the compounds, especially hybrids **29** and **34**, showed promising activity against the extracellular trypomastigote stage. The discrepancy between activity profiles across parasite stages raises important questions regarding the underlying mechanism of action. Notably, inhibitors targeting sterol 14α-demethylase (CYP51)—such as ravuconazole and posaconazole—have shown limited clinical success in treating Chagas disease despite exhibiting potent *in vitro* activity at subnanomolar concentrations against amastigotes.^28,61^ Their mechanism involves a pathway that is crucial for maintaining membrane integrity and functionality in the replicative forms of the parasite. However, the hybrids synthesized in this study—despite sharing some structural similarity with azole-based CYP51 inhibitors, particularly in the presence of 1,2,3-triazole or imidazole moieties failed to replicate this activity, suggesting that their mechanism of action does not involve disruption of the sterol biosynthetic pathway. It is also plausible that the structural features essential for activity against the trypomastigotes—such as nitroaromatic substitution and benzylic moieties are insufficient to exhibit effects within the intracellular environment, where distinct molecular targets and drug accessibility barriers are involved. Supporting this hypothesis, previous studies have demonstrated that certain nitro heterocyclic drugs, such as benznidazole, exert stage-dependent activity influenced by the metabolic competence of the parasite to bioactivate the prodrug.^54,62^ Hence, it is conceivable that the observed inactivity of the hybrids against amastigotes may be linked to a failure in bioactivation. Altogether, these findings highlight the importance of evaluating the activity across multiple stages of *T. cruzi* and underscore the need for mechanistic studies to elucidate the specific molecular targets of these hybrid compounds. Structural optimization efforts should also consider physicochemical properties that enhance cell permeability, particularly if the aim is to improve the efficacy against the intracellular forms responsible for disease persistence and progression.

Among the evaluated parameters, the absence of cytotoxic effects against H9c2 cardiac cells was one of the most relevant findings, as all 17 eugenol-based hybrids demonstrated no detectable toxicity at concentrations up to 1400 μmol·L^–1^ (Table 1). This result is particularly encouraging, given that H9c2 cells are widely employed in toxicological assays related to Chagas disease, and early detection of cardiotoxicity is essential in the development of antiparasitic agents intended for chronic administration. Similarly, most of the tested compounds also exhibited low cytotoxic potential against Vero cells, with CC_50_ values exceeding 1300 μmol·L^–1^, further supporting their overall safety profile. However, hybrids **20**, **25**, and **33** represented notable exceptions, exhibiting significantly higher cytotoxicity toward Vero cells (Figure 6). This observation suggests that subtle variations in the molecular structures of these compounds may critically influence their interaction with mammalian cells. One plausible explanation involves the presence of nitroaromatic substituents, structural motifs known to play a pivotal role in antiparasitic activity^63^ but also widely recognized as toxicophores due to their propensity to undergo bioactivation into reactive intermediates.^64^ Hybrid **25**, which presented the highest cytotoxicity, contains both a nitro group and a free phenolic hydroxyl moiety—an arrangement associated with increased redox reactivity and potential oxidative stress induction, which may account for the observed toxicity.

**Figure 6:**
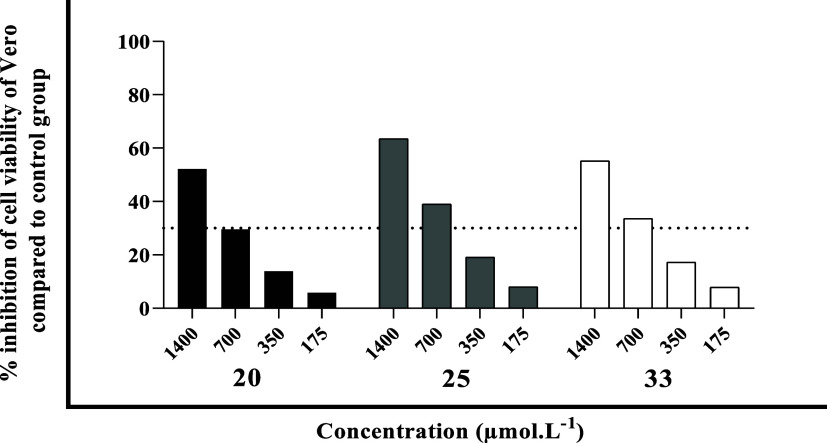
Inhibition percentage of cell viability of Vero cells incubated for 48 h with serial dilution of 1400 to 175 μmol·L^–1^ of hybrids, evaluated by resazurin.

To further explore the balance between efficacy and safety, selectivity indices (SI) were calculated for the active compounds (Table 1). Compound **29** exhibited an SI greater than 49, while compound **34** showed an SI above 34, indicating a favorable therapeutic window. These findings reinforce the potential of selected eugenol-based hybrids as trypanosomicidal agents while simultaneously emphasizing the importance of structure–toxicity relationships (STR) in guiding future optimization. Further investigations, including mechanistic toxicology and metabolic profiling, may provide deeper insight into the safety liabilities associated with specific structural motifs and aid in the rational refinement of these hybrid molecules.

Figure 7 provides a concise visual summary of the main Structure–Activity Relationship (SAR) findings for the synthesized eugenol-based hybrids, focusing on their trypanosomicidal activity against *T. cruzi*. This figure illustrates the crucial structural elements that influenced the potency and selectivity of the compounds as determined by biological assays. The figure highlights that the azole ring (triazole or imidazole) is an essential component for trypanocidal activity. The absence of these groups resulted in a significant loss of activity. Compound **29**, which showed the highest activity (EC_50_ = 26.5 μmol L^–1^), possesses a nitroaromatic group. The presence of this group is strongly correlated with activity against trypomastigotes, suggesting a mechanism of action that may involve the reduction of the nitro group, similar to the case for benznidazole. Variation or the absence of this group in other compounds led to a notable reduction in potency. The benzyl group appears to contribute positively to the activity. The most active compounds, such as **29** and **34**, share the presence of this substituent, indicating that the nature and size of the group are important for molecular recognition or optimization of physicochemical properties. The figure may also illustrate that other modifications, such as variations in the spacer between the azole ring and the eugenol core or the presence of different functional groups at other positions, can lead to decreased activity or increased toxicity, highlighting the delicate balance between efficacy and selectivity. In summary, the SAR revealed in Figure 7 provides clear guidelines for the future design of new trypanosomicidal agents. The activity profile indicates that the optimization of eugenol-based hybrids should prioritize the incorporation of nitroaromatic groups and the maintenance of a specific structure in the azole ligand while seeking a favorable balance in drug-likeness properties. Understanding these parameters is fundamental to guiding the synthesis of more potent and selective analogs with the potential to advance to more in-depth *in vivo* studies.

**Figure 7:**
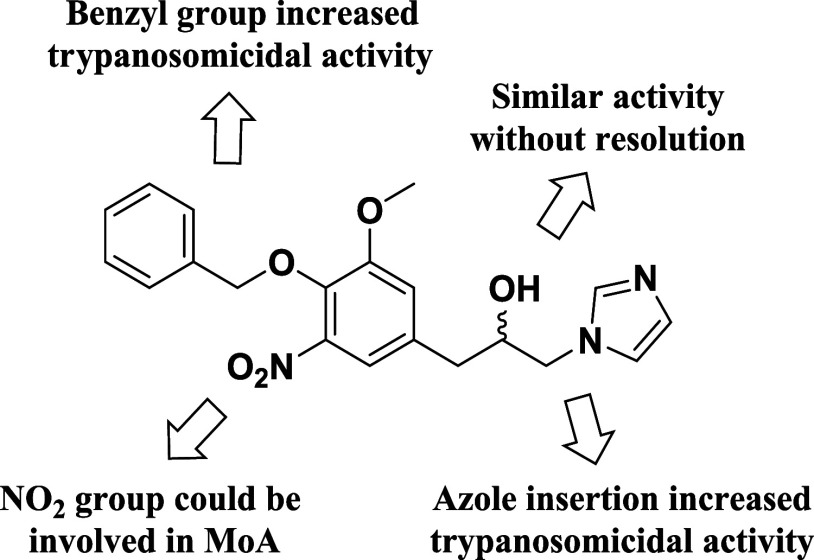
Summary of SAR for the observed trypanosomicidal activity.

Computer-aided drug discovery tools facilitated early-stage screening of physicochemical and pharmacokinetic parameters for the synthesized eugenol-azole hybrids^65^ (**18**–**34**). Thus, the synthesized compounds were evaluated via the SwissADME online platform (http://www.swissadme.ch/). These *in silico* analyses, based on Lipinski’s Rule of Five, assessed drug-likeness and oral bioavailability (Table 3). All hybrid compounds complied with Lipinski’s parameters, exhibiting molecular weights from 248.28 to 384.39 g/mol and acceptable CLog *P* values. Most also showed favorable polar surface area (PSA) values (<140 Å^2^), indicating good membrane permeability and intestinal absorption, even for derivatives with higher HBA values. This suggests that the structural modifications preserved compliance with key drug-likeness criteria and a promising oral bioavailability profile.

**Table 3:** Physicochemical Properties Calculated for the Hybrids

Cpd.	MW	CLog *p*	HBD	HBA	PSA
Optimal range	<500	<5	<5	<10	<140
**18**	249.27	0.31	2	5	80.40
**19**	294.26	–0.58	2	7	126.22
**20**	263.29	0.58	1	5	69.40
**21**	308.29	–0.31	1	7	115.22
**22**	339.39	1.83	1	5	69.40
**23**	384.39	0.94	1	7	115.22
**24**	248.28	0.18	2	4	67.51
**25**	293.28	–0.75	2	6	113.33
**26**	262.30	0.44	1	4	56.51
**27**	307.30	–0.48	1	6	102.33
**28**	338.40	1.65	1	4	56.51
**29**	383.40	0.73	1	6	102.33
**30**	249.27	0.31	2	5	80.40
**31**	294.26	–0.58	2	7	126.22
**32**	263.29	0.58	1	5	69.40
**33**	308.29	–0.31	1	7	115.22
**34**	384.39	0.94	1	7	115.22
**Bz**	260.24	0.9	1	4	92.7

Specifically, hybrid **29**, the most active, compared favorably with benznidazole. Both compounds adhered to Lipinski’s rule, supporting their potential as orally bioavailable drug candidates. Notably, hybrid **29** displayed a lower molecular weight and comparable lipophilicity and hydrogen bonding capacity. Its PSA was also within limits, suggesting favorable membrane permeability and potential CNS accessibility. These findings collectively support that the structural modifications in the hybrid series maintained the physicochemical properties required for good oral bioavailability, aligning them with an established therapeutic agent and supporting their potential for further evaluation.

## 3 Conclusions

This research successfully demonstrated a promising strategy in the design, synthesis, and evaluation of novel eugenol-based hybrids aimed at the development of more effective and safer therapies for Chagas disease. By integrating known trypanosomicidal pharmacophores with the eugenol scaffold, the results revealed that hybrid **29** emerged as a good starting point for further SAR exploration. Its good trypanosomicidal activity against the trypomastigote forms of *T. cruzi* (Y strain), with an EC_50_ of 26.5 μmol·L^–1^, and its promising selectivity (SI > 49) toward mammalian cells, positioning it favorably for further investigation, surpassing benznidazole in terms of *in vitro* toxicity.

The relevance of the nitroaromatic substituent and benzyl group in the observed trypanosomicidal activity against trypomastigotes suggests it may act as other compounds presenting these functionalities, which is under investigation in computational and biochemical studies by our group. The excellent drug-likeness properties and compliance with Lipinski’s Rule of Five, coupled with favorable PSA values, reinforce the promising pharmacokinetic profile of hybrid **29**, indicating good oral absorption and bioavailability.

However, it is crucial to acknowledge the limitations found in the absence of significant activity against the intracellular amastigote forms of *T. cruzi*. This gap could be attributed to challenges in the compound’s penetration into host cells or its bioactivation within the intracellular environment. Future investigations should therefore focus on the structural optimization of these hybrids to improve their ability to reach and eliminate amastigote forms, which are crucial for the chronic phase of the infection.

In summary, this study validates molecular hybridization as a potent strategy in the search for new antiparasitic drug candidates.

## 4 Materials and Methods

### 4.1 Chemistry

#### 4.1.1 Generalities

All reagents were purchased from Sigma-Aldrich (São Paulo, Brazil) and used without further purification except DMF, which was dried before use. Reaction progress was monitored by TLC on silica gel plates with aluminum backing and a fluorescent indicator (Macherey-Nagel, ALUGRAM Xtra Sil G/UV254). Products were purified by column chromatography using silica gel 60 (70–230 mesh, Sorbline) or neutral alumina. Melting points were determined using a Bücher 535 apparatus (0–300 °C), calibrated with Merck P.A. vanillin (M.P.: 80–81 °C). IR spectra were recorded on a Thermo Scientific Nicolet-iS50 spectrometer with an ATR accessory, and values were reported in wavenumbers (υ̅_max_, cm^–1^). ^1^H and ^13^C NMR spectra were obtained on a Bruker AC-300 spectrometer using CDCl_3_ or DMSO-*d*_6_ as the solvent and TMS or residual CHCl_3_ as the internal reference. Chemical shifts (δ) are reported in ppm; multiplicities as s (singlet), d (doublet), dd (double doublet), ddd (double doublet of doublets), dt (double triplet), dtd (double triplet of doublets), and m (multiplet). Coupling constants (*J*) are given in Hz, and proton integration was used to determine hydrogen counts. Mass spectra were recorded on an Acquity UPLC system (Waters, USA) coupled to a single quadrupole mass analyzer (Acquity QDa) with electrospray ionization (ESI), which was also used to assess purity. Enantiomeric separation was performed using a Jasco 880-PU Intelligent HPLC Pump (Japan) with manual injection (20 μL loop), coupled to a Jasco 875-UV UV/vis detector, a Jasco OR-2090Plus chiral polarimeter, and a Jasco LC-Net II/ADC controller. Compounds **2**,^41,42^ **3**,^66^ **4**,^42^ **5**,^67^ and **7** ^68^ were synthesized as previously.

#### 4.1.2 General Procedures for the Synthesis and Structural Characterization of Eugenol Derivatives (**6** and **8**–**34**)

##### 4.1.2.1 Benzyl Ether Derivative **6**

The compound **2** (4.78 mmol; 1.0 equiv) was dissolved in *N*,*N*-dimethylformamide (DMF, 20 mL) and stirred with anhydrous potassium carbonate (19.14 mmol; 4.0 equiv) for 5 min at room temperature. Benzyl chloride (4.78 mmol; 1.0 equiv) was then added dropwise, and the reaction mixture was refluxed at 70 °C overnight. After completion, the mixture was filtered under reduced pressure, and the solvent was removed by using a rotary evaporator. The resulting residue was dissolved in ethyl acetate and sequentially washed with 2 mol·L^–1^ NaOH solution and saturated sodium chloride solution. The organic layer was dried over anhydrous sodium sulfate, filtered, and concentrated under reduced pressure. The crude product was purified by column chromatography on silica gel by using a mixture of hexane and ethyl acetate (9:1, v/v) as the eluent.

##### 4.1.2.2 5-Allyl-2-(benzyloxy)-1-methoxy-3-nitrobenzene (**6**)

From **2**. Yellow oil; yield 100%. IR (υ̅_max_, in cm^–1^): 2930 (C–H), 1614 (C = C_ar_), 1275 (C–O). ^1^H NMR (δ ppm; CDCl_3_, 300 MHz): 7.15 (d; ^4^ *J*_H3,H5_ = 2.0 Hz; 1H, H-3), 6.93 (d; ^4^ *J*_H5,H3_ = 2.0; 1H, H-5), 3.90 (s; 3H, H-7), 3.37 (d; ^3^ *J*_H8,H9_ = 6.7 Hz; 2H, H-8), 5.92 (dtd; ^3^ *J*_H9,H8_ = 6.7 Hz, ^3^ *J*_H9,H10a_ = 10.3 Hz, ^3^ *J*_H9,H10b_ = 16.9 Hz; 1H, H-9), 5.09 (m; 1H, H-10a), 5.05 (m; 1H, H-10b), 5.13 (s; 2H, H-11). ^13^C NMR (δ ppm; CDCl_3_, 75 MHz): 154.1 (C-2), 145.1 (C-6), 139.8 (C-1), 135.8 (C-9), 136.5 (C-4), 117.3 (C-10), 116.5 (C-3), 115.8 (C-5), 56.5 (C-7), 76.0 (C-11), 39.6 (C-8).

##### 4.1.2.3 Epoxide Derivatives (**8**–**12**)

The allyl derivative (**2**, **3**, **4**, **5**, or **6**; 1 mmol; 1.0 equiv) was dissolved in chloroform (30 mL) and cooled in an ice bath under an argon atmosphere. A solution containing *m*-chloroperbenzoic acid (2 mmol; 2.0 equiv) in chloroform was added, and the reaction mixture was stirred overnight under the same conditions. Following this period, the reaction mixture was neutralized by washing with saturated sodium bicarbonate solution, followed by distilled water. The organic layer was dried over anhydrous sodium sulfate, filtered, and concentrated under reduced pressure. The crude product was purified by column chromatography on silica gel, using a gradient of hexane and ethyl acetate (from 10:0 to 8:2, v/v) as eluent.

##### 4.1.2.4 (±)-2-Methoxy-6-nitro-4-(oxiran-2-ylmethyl)­phenol (**8**)

From **2**. Orange semisolid; yield 52%. IR (υ̅_max_, in cm^–1^): 3230 (O–H), 2969 (C–H), 1622 (C = C_ar_), 1537,1332 (NO_2_), 1265 (C–O–C). ^1^H NMR (δ ppm; CDCl_3_, 300 MHz): 7.52 (d; ^4^ *J*_H3,H5_ = 1.9 Hz; 1H; H-3), 7.02 (d; ^4^ *J*_H5,H3_ = 1.9 Hz; 1H; H-5), 3.91 (s; 3H; H-7), 2.69 (dd; ^3^ *J*_H8a,H9_ = 6.5 Hz, ^2^ *J*_H8a,H8b_ = 14.9 Hz; 1H; H-8a), 2.92 (dd; ^3^ *J*_H8b,H9_ = 4.0 Hz, *J*_H8b,H8a_ = 14.8 Hz; 1H; H-8b), 3.11 (dtd; ^3^ *J*_H9,H10b_ = 2.7 Hz, ^3^ *J*_H9,H10a_ = 3.9 Hz, ^3^ *J*_H9,H8a_ = 6.6 Hz; 1H; H-9), 2.79 (dd; ^3^ *J*_H10a,H9_ = 4.0 Hz, ^2^ *J*_H10a,H10b_ = 4.7 Hz; 1H; H-10b). ^13^C NMR (δ ppm; CDCl_3_, 75 MHz): 149.9 (C-2), 145.2 (C-6), 133.5 (C-1), 128.5 (C-4), 118.9 (C-3), 115.5 (C-5), 56.7 (C-7), 51.9 (C-9), 46.6 (C-10), 37.9 (C-8).

##### 4.1.2.5 (±)-2-(3,4-Dimethoxybenzyl)­oxirane (**9**)

From **3**. Brown oil; yield 45%. IR (υ̅_max_, in cm^–1^): 2936 (C–H), 1590 (C = C_ar_), 1257 (C–O–C).). ^1^H NMR (δ ppm; CDCl_3_, 300 MHz): 6.80–6.74 (m; 3H; H-5), 3.85 (s; 3H; H-7), 2.80–2.73 (m; 3H; H-8), 3.10 (dtd; ^3^ *J*_H9,H10b_ = 2.7 Hz, ^3^ *J*_H9,H10a_ = 3.9 Hz, ^3^ *J*_H9,H8a_ = 5.4 Hz; 1H; H-9), 2.80–2.73 (m; 3H; H-10a), 2.51 (dd; ^3^ *J*_H10b,H9_ = 2.7 Hz, ^2^ *J*_H10b,H10a_ = 4.9 Hz; 1H; H-10b), 3.83 (s; 3H; H-11). ^13^C NMR (δ ppm; CDCl_3_, 75 MHz): 148.9 (C-2), 147.8 (C-1), 129.7 (C-4), 120.9 (C-3), 112.2 (C-5), 111.2 (C-6), 55.9 (C-7), 55.8 (C-11), 52.6 (C-9), 46.9 (C-10), 38.3 (C-8).

##### 4.1.2.6 (±)-2-(3,4-Dimethoxy-5-nitrobenzyl)­oxirane (**10**)

From **4**. Yellow oil; yield 42%. IR (υ̅_max_, in cm^–1^): 2943 (C–H), 1614 (C = C_ar_), 1528,1359 (NO_2_), 1278 (C–O–C). ^1^H NMR (δ ppm; CDCl_3_, 300 MHz): 7.18 (d; ^4^ *J*_H3,H5_ = 2.0 Hz; 1H; H-3), 6.99 (d; ^4^ *J*_H5,H3_ = 2.0 Hz; 1H; H-5), 3.92 (s; 3H; H-7), 2.72 (dd; ^3^ *J*_H8a,H9_ = 6.5 Hz, ^2^ *J*_H8a,H8b_ = 14.8 Hz; 1H; H-8a), 2.93 (dd; ^3^ *J*_H8b,H9_ = 4.1 Hz, ^2^ *J*_H8b,H8a_ = 14.8 Hz; 1H; H-8b), 3.13 (dtd; ^3^ *J*_H9,H10a_ = 2.6 Hz, ^3^ *J*_H9,H10b_ = 4.0 Hz, ^3^ *J*_H9,H8a_ = 6.6 Hz; 1H; H-9), 2.80 (dd; ^3^ *J*_H10a,H9_ = 3.9 Hz, ^2^ *J*_H10a,H10b_ = 4.8 Hz; 1H; H-10a), 2.52 (dd; ^3^ *J*_H10b,H9_ = 2.6 Hz, ^2^ *J*_H10b,H10a_ = 4.8 Hz; 1H; H-10b), 3.89 (s; 3H; H-11). ^13^C NMR (δ ppm; CDCl_3_, 75 MHz): 154.0 (C-2), 144.6 (C-6), 141.5 (C-1), 133.5 (C-4), 116.8 (C-3), 116.0 (C-5), 61.9 (C-7), 56.4 (C-11), 51.8 (C-9), 46.6 (C-10).

##### 4.1.2.7 (±)-2-(4-(Benzyloxy)-3-methoxybenzyl)­oxirane (**11**)

From **5**. Colorless oil; yield 52%. IR (υ̅_max_, in cm^–1^): 2926 (C–H), 1599 (C = C_ar_), 1260 (C–O–C), 844 (epoxide). ^1^H NMR (δ ppm; CDCl_3_, 300 MHz): 6.87 (m; 3H; H-3, H-5, H-6), 3.89 (s; 3H; H-7), 2.79 (m; 3H; H-8a, H-8b, H-10a), 2.54 (dd; ^3^ *J*_H10b,H9_ = 2.6 Hz, ^2^ *J*_H10b,H10a_ = 4.8 Hz; 1H; H-10b), 5.14 (s; 2H; H-11). ^13^C NMR (δ ppm; CDCl_3_, 75 MHz): 149.7 (C-2), 112.8 (C-6), 147.0 (C-1), 130.4 (C-4), 120.9 (C-3), 114.2 (C-5), 56.0 (C-7), 71.1 (C-11), 52.6 (C-9), 46.9 (C-10).

##### 4.1.2.8 (±)-2-(4-(Benzyloxy)-3-methoxy-5-nitrobenzyl)­oxirane (**12**)

From **6**. Yellow semisolid; yield 49%. IR (υ̅_max_, in cm^–1^): 2930 (C–H), 1666 (C = C_ar_), 1533,1361 (NO_2_), 1281 (C–O–C), 837 (epoxide). ^1^H NMR (δ ppm; CDCl_3_, 300 MHz): 7.22 (d; ^4^ *J*_H3,H5_ = 2.1; 1H; H-3), 7.04 (d; ^4^ *J*_H5,H3_ = 2.0; 1H; H-5), 3.94 (s; 3H; H-7), 2.77 (dd; ^3^ *J*_H8a,H9_ = 6.4 Hz, ^2^ *J*_H8a,H8b_ = 14.9 Hz; 1H; H-8a), 2.97 (dd; ^3^ *J*_H8b,H9_ = 4.2 Hz, ^2^ *J*_H8b,H8a_ = 14.8 Hz; 1H; H-8b), 5.15 (s; 2H; H-11). ^13^C NMR (δ ppm; CDCl_3_, 75 MHz): 154.2 (C-2), 145.1 (C-6), 140.3 (C-1), 133.7 (C-4), 117.0 (C-3), 116.2 (C-5), 56.5 (C-7), 76.0 (C-11), 51.8 (C-9), 46.6 (C-10).

##### 4.1.2.9 Azide Derivatives (**13**–**17**)

The corresponding epoxide (**7**, **8**, **9**, **10**, or **12**; 0.5 mmol; 1.0 equiv) was dissolved in *N*,*N*-dimethylformamide (DMF, 10 mL) and stirred with sodium azide (1.5 mmol; 3.0 equiv) under reflux at 80 °C for 24 h. After completion, the solvent was removed under a gentle stream of air. The resulting residue was purified by column chromatography on neutral alumina using a gradient of chloroform and methanol (up to 9:1, v/v) as the eluent.

##### 4.1.2.10 (±)-4-(3-Azido-2-hydroxypropyl)-2-methoxyphenol (**13**)

From **7**. Yellow oil; yield 33%. IR (υ̅_max_, in cm^–1^): 3434 (O–H), 2936 (C–H), 2101 (N=N=N), 1605 (C = C_ar_), 1271 (C–O–C). ^1^H NMR (δ ppm; CDCl_3_, 300 MHz): 6.83 (d; ^4^ *J*_H3,H5_ = 7.9 Hz, 1H; H-3), 6.69–6.65 (m; 2H; H-5), 3.85 (s; 3H; H-6), 2.67 (dd; ^3^ *J*_H8a,H9_ = 6.2 Hz, ^2^ *J*_H8a,H8b_ = 12.5 Hz; 1H; H-8a), 2.74 (dd; ^3^ *J*_H8b, H9_ = 4.5 Hz, ^2^ *J*_H8b,H8a_ = 12.5 Hz; 1H; H-8b), 3.97–3.91 (m; 1H; H-9), 3.26 (dd; ^3^ *J*_H10a,H9_ = 6.7 Hz, ^2^ *J*_H10a,H10b_ = 12.5 Hz; 1H; H-10a), 3.35 (dd; ^3^ *J*_H10b,H9_ = 3.8 Hz, ^2^ *J*_H10b,H10a_ = 12.5 Hz; 1H; H-10b). ^13^C NMR (δ ppm; CDCl_3_, 75 MHz): 146.6 (C-2), 144.4 (C-1), 128.8 (C-4), 121.9 (C-5), 114.6 (C-6), 111.8 (C-3), 71.9 (C-9), 55.9 (C-10), 55.9 (C-7), 40.5 (C-8).

##### 4.1.2.11 (±)-4-(3-Azido-2-hydroxypropyl)-2-methoxy-6-nitrophenol (**14**)

From **8**. Orange oil; yield 39%. IR (υ̅_max_, in cm^–1^): 3391 (O–H), 2928 (C–H), 2101 (N=N=N), 1644 (C = C_ar_), 1544; 1363 (NO_2_), 1264 (C–O–C). ^1^H NMR (δ ppm; CDCl_3_, 300 MHz): 7.53 (d; ^4^ *J*_H3,H5_ = 2.0 Hz, 1H; H-3), 7.01 (d; ^4^ *J*_H3,H5_ = 1.9 Hz, 1H; H-5), 3.92 (s; 3H; H-7), 2.72 (dd; ^3^ *J*_H8a,H9_ = 7.0 Hz, ^2^ *J*_H8a,H8b_ = 13.3 Hz; 1H; H-8a), 2.78 (dd; ^3^ *J*_H8b,H9_ = 4.5 Hz, ^2^ *J*_H8b,H8a_ = 13.3 Hz; 1H; H-8b), 4.01–3.94 (m; 1H; H-9), 3.29 (dd; ^3^ *J*_H10a,H9_ = 6.8 Hz, ^2^ *J*_H10a,H10b_ = 12.4 Hz, 1H; H-10a), 3.42 (dd; ^3^ *J*_H10b,H9_ = 3.8 Hz, ^2^ *J*_H10b,H10a_ = 12.4 Hz; 1H; H-10b).). ^13^C NMR (δ ppm; CDCl_3_, 75 MHz): 149.9 (C-2), 145.2 (C-6), 133.5 (C-1), 128.6 (C-4), 119.1 (C-5), 115.9 (C-3), 71.2 (C-9), 56.8 (C-7), 56.2 (C-10), 39.9 (C-8).

##### 4.1.2.12 (±)-1-Azido-3-(3,4-dimethoxyphenyl)­propan-2-ol (**15**)

From **9**. Colorless oil; yield 31%. IR (υ̅_max_, in cm^–1^): 3406 (O–H), 2925 (C–H), 2099 (N=N=N), 1592 (C = C_ar_), 1261 (C–O–C). ^1^H NMR (δ ppm; CDCl_3_, 300 MHz): 6.81 (d; ^4^ *J*_H3,H5_ = 7.9 Hz, 1H; H-3), 6.75–6.71 (m; 2H; H-5; H-6), 3.86 (s; 3H; H-7), 2.70 (dd; ^3^ *J*_H8a,H9_ = 6.8 Hz, ^2^ *J*_H8a,H8b_ = 12.9 Hz; 1H; H-8a), 2.77 (dd; ^3^ *J*_H8b, H9_ = 4.8 Hz, ^2^ *J*_H8b,H8a_ = 13.0 Hz; 1H; H-8b), 4.00–3.92 (m; 1H; H-9), 3.28 (dd; ^3^ *J*_H10a,H9_ = 6.7 Hz, ^2^ *J*_H10a,H10b_ = 12.5 Hz; 1H; H-10a), 3.37 (dd; ^3^ *J*_H10b,H9_ = 3.8 Hz, ^2^ *J*_H10b,H10a_ = 12.5 Hz; 1H; H-10b), 3.85 (s; 3H; H-11). ^13^C NMR (δ ppm; CDCl_3_, 75 MHz): 154.0 (C-2), 144.6 (C-6), 141.5 (C-1), 133.7 (C-4), 117.2 (C-5), 116.3 (C-3), 71.2 (C-9), 62.0 (C-7), 56.5 (C-11), 59.1 (C-10), 40.1 (C-8).

##### 4.1.2.13 (±)-1-Azido-3-(3,4-dimethoxy-5-nitrophenyl)­propan-2-ol (**16**)

From **10**. Yellow oil; yield 36%. IR (υ̅_max_, in cm^–1^): 3374 (O–H), 2942 (C–H), 2097 (N=N=N), 1614 (C = C_ar_), 1529; 1358 (NO_2_), 1278 (C–O–C). ^1^H NMR (δ ppm; CDCl_3_, 300 MHz): 7.16 (d; ^4^ *J*_H3,H5_ = 2.0 Hz; 1H; H-3), 6.96 (dd; ^4^ *J*_H5,H3_ = 1.9 Hz; 1H; H-5), 3.92 (s; 3H; H-7), 2.73 (dd; ^3^ *J*_H8a,H9_ = 6.6 Hz, ^2^ *J*_H8a,H8b_ = 12.8 Hz; 1H; H-8a), 2.79 (dd; ^3^ *J*_H8b, H9_ = 4.1 Hz, ^2^ *J*_H8b,H8a_ = 12.8 Hz; 1H; H-8b), 4.02–3.94 (m; 1H; H-9), 3.29 (dd; ^3^ *J*_H10a,H9_ = 6.8 Hz, ^2^ *J*_H10a,H10b_ = 12.5 Hz, 1H; H-10a), 3.40 (dd; ^3^ *J*_H10b,H9_ = 3.8 Hz, ^2^ *J*_H10b,H10a_ = 12.4 Hz; 1H; H-10b), 3.89 (s; 3H; H-11). ^13^C NMR (δ ppm; CDCl_3_, 75 MHz): 154.0 (C-2), 144.6 (C-6), 141.5 (C-1), 133.7 (C-4), 117.2 (C-5), 116.3 (C-3), 71.2 (C-9), 62.0 (C-7), 56.5 (C-11), 56.1 (C-10), 40.1 (C-8).

##### 4.1.2.14 (±)-1-Azido-3-(4-(benzyloxy)-3-methoxy-5-nitrophenyl)­propan-2-ol (**17**)

From **12**. Black oil; yield 51%. IR (υ̅_max_, in cm^–1^): 3396 (O–H), 2930 (C–H), 2100 (N=N=N), 1662 (C = C_ar_), 1531; 1361 (NO_2_), 1280 (C–O–C). ^1^H NMR (δ ppm; CDCl_3_, 300 MHz): 7.08 (d; ^4^ *J*_H3,H5_ = 2.0 Hz; 1H; H-3), 7.20 (dd; ^4^ *J*_H5,H3_ = 2.0 Hz; 1H; H-5), 3.93 (s; 3H; H-7), 2.77 (dd; ^3^ *J*_H8a,H9_ = 7.3 Hz, ^2^ *J*_H8a,H8b_ = 14.1 Hz; 1H; H-8a), 2.79 (m; 1H; H-8b), 4.50 (m; 1H; H-9), 1.21 (dd; ^3^ *J*_H10a,H9_ = 2.4 Hz, ^2^ *J*_H10a,H10b_ = 6.0 Hz, 1H; H-10a), 1.35 (m; 1H; H-10b), 5.13 (s; 3H; H-11). ^13^C NMR (δ ppm; CDCl_3_, 75 MHz): 154.3 (C-2), 136.4 (C-1), 130.6 (C-4), 117.4 (C-5), 116.5 (C-3), 70.8 (C-9), 56.6 (C-7), 76.0 (C-11), 54.5 (C-10), 40.2 (C-8).

##### 4.1.2.15 1,2,4-Triazole and Imidazole Derivatives (**18**–**29**)

The corresponding epoxide (**7**, **8**, **9**, **10**, **11**, or **12**; 0.5 mmol; 1.0 equiv) was dissolved in DMF/H_2_O (1:1; 10 mL) and stirred with 1,2,4-triazole or imidazole (1.5 mmol; 3.0 equiv) under reflux at 80 °C for 24 h. After completion, the solvent was removed under a gentle stream of air, and the resulting residue was purified by column chromatography on neutral alumina using a chloroform/methanol gradient (up to 9:1, v/v) as the eluent.

##### 4.1.2.16 (±)-4-(2-Hydroxy-3-(1*H*-1,2,4-triazol-1-yl)­propyl)-2-methoxyphenol (**18**)

From **7**. White solid; yield 34%; m.p.: 123–125 °C. IR (υ̅_max_, in cm^–1^): 3419 (O–H), 2919 (C–H), 1605 (C = C_ar_), 1277 (C–O–C). ^1^H NMR (δ ppm; CDCl_3_, 300 MHz): 6.82 (d; ^4^ *J*_H3,H5_ = 7.9 Hz; 1H; H-3), 6.66 (dd; ^3^ *J*_H5,H6_ = 1.9 Hz, ^4^ *J*_H5,H3_ = 7.9 Hz; 1H; H-5), 6.69 (d; ^3^ *J*_H6,H5_ = 1.7 Hz; 1H; H-6), 3.83 (s; 3H; H-7), 2.74–2.66 (m; 2H; H-8a; H-8b), 4.17–4.03 (m; 2H; H-9; H-10a), 4.32 (ddd; ^3^ *J*_H10b,H9_ = 2.9 Hz, ^2^ *J*_H10b,H10a_ = 12.3 Hz; 1H; H-10b), 8.06 (s; 1H; H-11), 7.87 (s; 1H; H-12). ^13^C NMR (δ ppm; CDCl_3_, 75 MHz): 151.7 (C-11), 146.7 (C-2), 144.6 (C-1), 144.1 (C-12), 128.5 (C-4), 121.9 (C-5), 114.6 (C-6), 111.8 (C-3), 71.1 (C-9), 55.9 (C-7), 54.4 (C-10), 40.6 (C-8). HRMS (ESI) calcd for C_12_H_15_O_3_N_3_ [M + H]^+^ 250.1113, found 250.1183.

##### 4.1.2.17 (±)-4-(2-Hydroxy-3-(1*H*-1,2,4-triazol-1-yl)­propyl)-2-methoxy-6-nitrophenol (**19**)

From **8**. Orange semisolid; yield 37%. IR (υ̅_max_, in cm^–1^): 3388 (O–H), 2917 (C–H), 1622 (C = C_ar_), 1531; 1324 (NO_2_), 1262 (C–O–C). ^1^H NMR (δ ppm; CDCl_3_, 300 MHz): 7.53 (d; ^4^ *J*_H3,H5_ = 2.0 Hz; 1H; H-3), 7.05 (d; ^4^ *J*_H5,H3_ = 1.9 Hz; 1H; H-5), 3.89 (s; 3H; H-7), 2.76 (ddd; ^3^ *J*_H8b,H9_ = 4.5 Hz, ^3^ *J*_H8a,H9_ = 13.8 Hz, ^2^ *J*_H8a,H8b_ = 21.9 Hz; 2H; H-8a, H-8b), 4.23–4.05 (m; 2H; H-9; H-10a), 4.29 (dd; ^3^ *J*_H10b,H9_ = 2.4 Hz, ^2^ *J*_H10b,H10a_ = 13.3 Hz; 1H; H-10b), 8.10 (s; 1H; h-11), 7.84 (s; 1H; H-12). ^13^C NMR (δ ppm; CDCl_3_, 75 MHz): 151.7 (C-11), 149.9 (C-2), 145.1 (C-6), 144.1 (C-12) 133.5 (C-1), 128.6 (C-4), 119.2 (C-3), 116.0 (C-5), 70.3 (C-9), 56.8 (C-7), 54.8 (C-10), 40.0 (C-8). HRMS (ESI) calcd for C_12_H_13_O_5_N_4_ [M + H]^+^ 295.0964, found 295.1033.

##### 4.1.2.18 (±)-1-(3,4-Dimethoxyphenyl)-3-(1*H*-1,2,4-triazol-1-yl)­propan-2-ol (**20**)

From **9**. Yellow oil; yield 32%. IR (υ̅_max_, in cm^–1^): 3392 (O–H), 2924 (C–H), 1593 (C = C_ar_), 1275 (C–O–C). ^1^H NMR (δ ppm; CDCl_3_, 300 MHz): 6.80 (d; ^4^ *J*_H3,H5_ = 8.7 Hz; 1H; H-3), 6.73 (dd; ^3^ *J*_H5,H6_ = 1.9 Hz, ^4^ *J*_H5,H3_ = 6.7 Hz; 2H; H-5; H-6), 3.85 (s; 3H; H-7), 2.76 (ddd; ^3^ *J*_H8b,H9_ = 2.5 Hz, ^3^ *J*_H8a,H9_ = 6.0 Hz, ^2^ *J*_H8a,H8b_ = 16.5 Hz; 2H; H-8a; H-8b), 4.26–4.05 (m; 2H; H-9; H-10a), 4.28 (dd; ^3^ *J*_H10b,H9_ = 2.4 Hz, ^2^ *J*_H10b,H10a_ = 13.3 Hz; 1H; H-10b), 8.08 (s; 1H; H-11), 7.91 (s; 1H; H-12), 3.84 (s; 3H; H-13). ^13^C NMR (δ ppm; CDCl_3_, 75 MHz): 151.8 (C-11), 149.1 (C-2), 148.0 (C-1), 144.1 (C-12), 129.1 (C-4), 121.3 (C-3), 112.3 (C-5), 111.3 (C-6), 71.1 (C-9), 55.9 (C-7), 55.9 (C-13), 54.3 (C-10), 40.5 (C-8). HRMS (ESI) calcd for C_13_H_17_O_3_N_3_ [M + H]^+^ 264.1270, found 264.1338.

##### 4.1.2.19 (±)-1-(3,4-Dimethoxy-5-nitrophenyl)-3-(1*H*-1,2,4-triazol-1-yl)­propan-2-ol (**21**)

From **10**. Yellow solid; yield 36%; m.p.: 123–125 °C. IR (υ̅_max_, in cm^–1^): 3290 (O–H), 2943 (C–H), 1614 (C = C_ar_), 1530; 1357 (NO_2_), 1276 (C–O–C). ^1^H NMR (δ ppm; CDCl_3_, 300 MHz): 7.15 (d; ^4^ *J*_H3,H5_ = 2.0 Hz; 1H; H-3), 6.98 (d; ^4^ *J*_H5,H3_ = 2.0; 1H; H-5), 3.85 (s; 3H; H-7), 2.73 (ddd; ^3^ *J*_H8b,H9_ = 6.2 Hz, ^3^ *J*_H8a,H9_ = 13.9 Hz, ^2^ *J*_H8a,H8b_ = 21.8 Hz; 2H; H-8a; H-8b), 4.21–4.01 (m; 2H; H-9; H-10a), 4.26 (dd; ^3^ *J*_H10b,H9_ = 2.5 Hz, ^2^ *J*_H10b,H10a_ = 13.1 Hz; 1H; H-10b), 8.10 (s; 1H; H-11), 7.78 (s; 1H; H-12), 3.82 (s; 3H; H-13). ^13^C NMR (δ ppm; CDCl_3_, 75 MHz): 153.9 (C-11), 153.8 (C-6), 144.4 (C-2), 144.1 (C-1), 144.1 (C-12), 133.9 (C-4), 117.5 (C-3), 116.3 (C-5), 70.0 (C-9) 62.0 (C-7), 54.9 (C-10), 40.3 (C-8). HRMS (ESI) calcd for C_13_H_16_O_5_N_4_ [M + H]^+^ 309.1121, found 309.1190.

##### 4.1.2.20 (±)-1-(4-(Benzyloxy)-3-methoxyphenyl)-3-(1*H*-1,2,4-triazol-1-yl)­propan-2-ol (**22**)

From **11**. Orange semisolid; yield 52%. IR (υ̅_max_, in cm^–1^): 3356 (O–H), 2925 (C–H), 1660 (C = C_ar_), 1261 (C–O–C). ^1^H NMR (δ ppm; CDCl_3_, 300 MHz): 6.75 (d; ^4^ *J*_3,5_ = 2.0 Hz; 1H; H-3), 6.67 (dd; ^4^ *J*_5,3_ = 2.0, ^3^ *J*_5,6_ = 8.1; 1H; H-5), 6.82 (d; ^3^ *J*_6,5_ = 8.1; 1H; H-6), 3.86 (s; 3H; H-7), 2.73–2.70 (m; 2H; H-8a, H-8b), 4.20–4.16 (m; 1H, H-9), 4.10–4.03 (m; 1H, H-10a), 4.29–4.23 (m; 1H, H-10b), 8.06 (s; 1H, H-11), 7.88 (s; 1H, H-12), 5.12 (s; 2H, H-13), 7.43–7.32 (m; 5H, H-15, H-16, H-17). ^13^C NMR (δ ppm; CDCl_3_, 75 MHz): 151.7 (C-11), 149.8 (C-2), 147.2 (C-1), 144.1 (C-12), 137.1 (C-14), 129.9 (C-4), 128.6 (C-16), 127.8 (C-17), 127.3 (C-15), 121.3 (C-3), 114.3 (C-5), 113.1 (C-6), 71.1 (C-13), 71.0 (C-9), 56.0 (C-7), 54.5 (C-10), 40.5 (C-8). HRMS (ESI) calcd for C_19_H_21_O_3_N_3_ [M + H]^+^ 340.1583, found 340.22.

##### 4.1.2.21 (±)-1-(4-(Benzyloxy)-3-methoxy-5-nitrophenyl)-3-(1*H*-1,2,4-triazol-1-yl)­propan-2-ol (**23**)

From **12**. Gray semisolid; yield 55%. IR (υ̅_max_, in cm^–1^): 3125 (O–H), 2900 (C–H), 1614 (C = C_ar_), 1283 (C–O–C), 1534; 1364 (NO_2_). ^1^H NMR (δ ppm; CDCl_3_, 300 MHz): 7.21 (d; ^4^ *J*_3,5_ = 2.1; 1H, H-3), 7.03 (d; ^4^ *J*_5,3_ = 2.0; 1H, H-5), 3.92 (s; 3H, H-7), 2.88–2.84 (m; 2H, H-8a, H-8b), 4.12–4.01 (m; 1H, H-9), 3.50 (dd; ^3^ *J*_10a,9_ = 6.1, ^2^ *J*_10a,10b_ = 11.2; 1H, H-10a), 3.61 (dd; ^3^ *J*_10b,9_ = 4.2, ^2^ *J*_10b,10a_ = 11.2; 1H, H-10b), 7.49–7.32 (m; 7H, H-11, H-12, H-15, H-16, H-17), 5.13 (s; 2H, H-13). ^13^C NMR (δ ppm; CDCl_3_, 75 MHz): 154.1 (C-2), 154.1 (C-11), 145.0 (C-6), 140.2 (C-1), 136.5 (C-14), 133.9 (C-4), 133.7 (C-12), 128.6 (C-16), 128.5 (C-17), 128.4 (C-15), 117.4 (C-3), 116.5 (C-5), 76.0 (C-13), 71.7 (C-9), 56.5 (C-7), 49.1 (C-10), 40.0 (C-8). HRMS (ESI) calcd for C_19_H_20_O_5_N_4_ [M + H]^+^ 385.1434, found 385.22.

##### 4.1.2.22 (±)-4-(2-Hydroxy-3-(1*H*-imidazol-1-yl)­propyl)-2-methoxyphenol (**24**)

From **7**. Brown oil; yield 29%. IR (υ̅_max_, in cm^–1^): 3361 (O–H), 2922 (C–H), 1591 (C = C_ar_), 1262 (C–O–C). ^1^H NMR (δ ppm; CDCl_3_, 300 MHz): 6.68–6.60 (m; 2H; H-3; H-5), 6.84 (d; ^3^ *J*_H6,H5_ = 7.9 Hz; 1H; H-6), 3.82 (s; 3H; H-7), 2.73–2.59 (m; 2H; H-8a; H-8b), 4.07–3.96 (m; 2H; H-9; H-10a), 3.89 (m; 1H; H-10b), 7.48 (s; 1H; H-11), 6.97 (d; ^3^ *J*_H12,H13_ = 5.8 Hz; 2H; H-12; H-13). ^13^C NMR (δ ppm; CDCl_3_, 75 MHz): 147.7 (C-2), 145.3 (C-1), 129.6 (C-4), 128.3 (C-11), 121.9 (C-12), 118.6 (C-13), 115.7 (C-3), 113.8 (C-5), 112.1 (C-6), 71.4 (C-9), 55.9 (C-7), 52.3 (C-10), 40.8 (C-8). HRMS (ESI) calcd for C_13_H_16_O_3_N_2_ [M + H]^+^ 249.1161, found 249.1228.

##### 4.1.2.23 (±)-4-(2-Hydroxy-3-(1*H*-imidazol-1-yl)­propyl)-2-methoxy-6-nitrophenol (**25**)

From **8**. Red semisolid; yield 39%.IR (υ̅_max_, in cm^–1^): 3408 (O–H), 2917 (C–H), 1622 (C = C_ar_), 1510; 1336 (NO_2_), 1234 (C–O–C). ^1^H NMR (δ ppm; DMSO, 300 MHz): 7.58 (s; 1H; H-13), 7.27 (d; *J* = 1.8 Hz; 1H; H-12), 7.15 (s; 1H), 6.99 (s; 1H), 6.87 (s; 1H), 4.05–3.99 (m; 2H), 3.80 (s; 3H), 3.43–3.24 (m; 2H), 2.66–2.45 (m; 1H). ^13^C NMR (δ ppm; CDCl_3_, 75 MHz): 172.6 (C-6), 150.8 (C-2), 138.2 (C-11), 136.4 (C-1), 128.3 (C-12), 126.9 (C-4), 120.6 (C-13), 117.2 (C-3), 116.9 (C-5), 70.1 (C-9), 56.7 (C-7), 52.4 (C-10), 40.3 (C-8). HRMS (ESI) calcd for C_13_H_15_O_5_N_3_ [M + H]^+^ 294.1012, found 249.1079.

##### 4.1.2.24 (±)-1-(3,4-Dimethoxyphenyl)-3-(1*H*-imidazol-1-yl)­propan-2-ol (**26**)

From **9**. Brown solid; yield 31%; m.p.: 130–133 °C. IR (υ̅_max_, in cm^–1^): 3393 (O–H), 2918 (C–H), 1589 (C = C_ar_), 1236 (C–O–C). ^1^H NMR (δ ppm; CDCl_3_, 300 MHz): 6.81 (s; 1H; H-3), 6.74 (d; ^3^ *J*_H6,H5_ = 6.7 Hz; 2H; H-5; H-6), 3.85 (s; 6H; H-7), 2.71 (d; ^3^ *J*_H8,H9_ = 6.6 Hz; 2H; H-8a; H-8b), 4.01 (d; ^3^ *J*_H9,H8=_ 10.9 Hz; 1H; H-9), 4.49 (s; 2H; H-10a, H-10b), 7.42 (s; 1H; H-11), 6.92 (s; 2H; H-12; H-13); 3.85 (s; 6H; H-14). ^13^C NMR (δ ppm; CDCl_3_, 75 MHz): 149.0 (C-2), 147.8 (C-1), 137.6 (C-11), 129.7 (C-4), 128.4 (C-12), 121.3 (C-13), 112.4 (C-5), 111.3 (C-6), 71.7 (C-9), 55.9 (C-7), 55.9 (C-14), 52.6 (C-10), 40.8 (C-8). HRMS (ESI) calcd for C_14_H_18_O_3_N_2_ [M + H]^+^ 263.1317, found 263.1382.

##### 4.1.2.25 (±)-1-(3,4-Dimethoxy-5-nitrophenyl)-3-(1*H*-imidazol-1-yl)­propan-2-ol (**27**)

From **10**. Orange semisolid; yield 42%. IR (υ̅_max_, in cm^–1^): 3456 (O–H), 2917 (C–H), 1615 (C = C_ar_), 1521; 1358 (NO_2_), 1281 (C–O–C). ^1^H NMR (δ ppm; CDCl_3_, 300 MHz): 6.94 (s; 1H; H-3), 6.91 (s; 1H; H-5), 3.91 (s; 3H; H-7), 2.73 (m; 2H; H-8a; H-8b), 3.94–3.87 (d; 1H; H-9), 4.09–3.99 (m; 2H; H-10a; H-10b), 7.43 (s; 1H; H-11), 6.99 (d; ^3^ *J*_H12,H13_ = 2.0 Hz; 1H; H-12), 7.18 (d; ^3^ *J*_H13,H12_ = 1.9 Hz; 1H; H-13), 3.86 (s; 3H; H-14). ^13^C NMR (δ ppm; CDCl_3_, 75 MHz): 153.4 (C-2), 144.5 (C-6), 140.1 (C-11), 136.2 (C-1), 128.4 (C-4), 123.2 (C-12), 120.6 (C-13), 118.6 (C-3), 116.1 (C-5), 70.7 (C-9), 61.9 (C-7), 56.9 (C-14), 52.6 (C-10), 40.3 (C-8). HRMS (ESI) calcd for C_14_H_17_O_5_N_3_ [M + H]^+^ 308.1168, found 308.1235.

##### 4.1.2.26 (±)-1-(4-(Benzyloxy)-3-methoxyphenyl)-3-(1*H*-imidazol-1-yl)­propan-2-ol (**28**)

From **11**. Gray semisolid; yield 44%. IR (υ̅_max_, in cm^–1^): 3200 (O–H), 2922 (C–H), 1590 (C = C_ar_), 1226 (C–O–C). ^1^H NMR (δ ppm; CDCl_3_, 300 MHz): 6.75 (d; ^4^ *J*_3,5_ = 2.0; 1H, H-3), 6.67 (dd; ^4^ *J*_5,3_ = 2.0, ^3^ *J*_5,6_ = 8.1; 1H, H-5), 6.83 (d; ^3^ *J*_6,5_ = 8.1; 1H, H-6), 3.85 (s; 3H, H-7), 2.71–2.67 (m; 2H, H-8a, H-8b), 4.06–4.00 (m; 2H, H-9, H-10a), 3.71–3.64 (m; 1H, H-10b), 7.59 (s; 1H, H-11), 6.96 (m; 2H, H-12, H-13), 5.12 (s; 2H, H-14), 7.48–7.29 (m; 5H, H-16, H-17, H-18). ^13^C NMR (δ ppm; CDCl_3_, 75 MHz): 149.8 (C-2), 147.1 (C-1), 137.8 (C-11), 135.1 (C-15), 128.9 (C-12), 128.6 (C-17), 127.9 (C-18), 127.3 (C-16), 121.9 (C-4), 121.3 (C-3), 119.9 (C-13), 114.1 (C-5), 113.1 (C-6), 71.8 (C-7), 71.2 (C-14), 56.0 (C-9), 52.4 (C-10), 40.8 (C-8). HRMS (ESI) calcd for C_20_H_22_O_3_N_2_ [M + H]^+^ 339.1630, found 339.23.

##### 4.1.2.27 (±)-1-(4-(Benzyloxy)-3-methoxy-5-nitrophenyl)-3-(1*H*-imidazol-1-yl)­propan-2-ol (**29**)

From **12**. Orange semisolid; yield 44%. IR (υ̅_max_, in cm^–1^): 3195 (O–H), 2921 (C–H), 1612 (C = C_ar_), 1280 (C–O–C), 1527; 1358 (NO_2_). ^1^H NMR (δ ppm; CDCl_3_, 300 MHz): 7.07 (s; 1H, H-3), 6.94 (s; 1H, H-5), 3.89 (s; 3H, H-7), 2.71 (dd; ^2^ *J*_8a,8b_ = 14.0; ^3^ *J*_8a,9_ = 8.7; 1H, H-8a), 2.80 (dd; ^2^ *J*_8b,8a_ = 14.0; ^3^ *J*_8b,9_ = 4.1; 1H, H-8b), 3.96–3.88 (m; 1H, H-9), 4.04 (dd; ^2^ *J*_10a,10b_ = 4.0; ^3^ *J*_10a,9_ = 8.7; 1H, H-10a), 4.08 (dd; ^2^ *J*_10b,10a_ = 7.7; ^3^ *J*_10b,9_ = 3.5; 1H, H-10b), 7.42 (s; 1H, H-11), 7.02 (d; ^3^ *J*_12,13_ = 2.1; 1H, H-12), 7.21 (d; ^3^ *J*_13,12_ = 2.0; 1H, H-13), 5.14 (s; 2H, H-14), 7.48–7.46 (m; 2H, H-16), 7.37–7.33 (m; 3H, H-17, H-18). ^13^C NMR (δ ppm; CDCl_3_, 75 MHz): 155.1 (C-2), 145.1 (C-6), 141.1 (C-1), 137.3 (C-11), 135.0 (C-15), 129.9 (C-12), 129.6 (C-17), 129.4 (C-16), 129.3 (C-18), 120.6 (C-13), 118.4 (C-3), 117.2 (C-5), 76.9 (C-14), 72.1 (C-9), 57.5 (C-7), 53.8 (C-10), 41.4 (C-8). HRMS (ESI) calcd for C_20_H_21_O_5_N_3_ [M + H]^+^ 384.1481, found 384.23.

##### 4.1.2.28 1,2,3-Triazole Derivatives (**30**–**34**)

Potassium carbonate (0.43 mmol; 1.2 equiv) was dissolved in a mixture of methanol and water (1:1, v/v, 10 mL). To this solution, the corresponding azide (13, 14, 15, 16, or 17; 0.27 mmol; 1.0 equiv), trimethylsilylacetylene (0.43 mmol; 1.2 equiv), copper­(II) sulfate (0.054 mmol; 0.2 equiv), and sodium ascorbate (0.108 mmol; 0.4 equiv) were sequentially added. The reaction mixture was stirred vigorously at room temperature overnight. Upon completion (monitored by TLC), the mixture was diluted with dichloromethane, washed with water, dried over anhydrous sodium sulfate, and concentrated under reduced pressure. The crude product was purified by column chromatography on neutral alumina using a chloroform/methanol (9:1, v/v) mixture as the eluent, affording the desired monosubstituted 1,2,3-triazoles (compounds 30–34).

In most cases, complete *in situ* desilylation was observed under the reaction conditions. However, for one compound, residual trimethylsilyl-protected triazole was detected. In this case, the crude product was dissolved in tetrahydrofuran (5 mL) and treated with tetrabutylammonium fluoride (0.27 mmol; 1.0 equiv) at room temperature for 24 h. After completion (monitored by TLC), the reaction mixture was processed by standard aqueous workup to yield the deprotected triazole.

##### 4.1.2.29 (±)-4-(2-Hydroxy-3-(1*H*-1,2,3-triazol-1-yl)­propyl)-2-methoxyphenol (**30**)

From **13**. White semisolid; yield 59%. IR (υ̅_max_, in cm^–1^): 3139 (O–H), 2941 (C–H), 1601 (C = C_ar_), 1269 (C–O–C). ^1^H NMR (δ ppm; CDCl_3_, 300 MHz): 6.71–6.67 (m; 2H, H-3, H-5), 6.82–6.79 (m; 1H, H-6), 3.82 (s; 3H, H-7), 2.65 (dd; ^3^ *J*_8a,9_ = 7.5, ^2^ *J*_8a,8b_ = 13.9; 1H, H-8a), 2.74 (m; 1H, H-8b), 4.31–4.20 (m; 2H, H-9, H-10a), 4.49 (dd; ^3^ *J*_10b,9_ = 2.5, ^2^ *J*_10b,10a_ = 13.4; 1H, H-10b), 7.67 (s; 1H, H-11), 7.60 (m; 1H, H-12). ^13^C NMR (δ ppm; CDCl_3_, 75 MHz): 146.8 (C-2), 144.7 (C-1), 133.4 (C-12), 128.5 (C-11), 125.1 (C-4), 122.0 (C-6), 114.7 (C-3), 112.1 (C-5), 71.4 (C-9), 55.9 (C-7), 54.9 (C-10), 40.1 (C-8). HRMS (ESI) calcd for C_12_H_15_O_3_N_3_ [M + H]^+^ 250.1113, found 250.21.

##### 4.1.2.30 (±)-4-(2-Hydroxy-3-(1*H*-1,2,3-triazol-1-yl)­propyl)-2-methoxy-6-nitrophenol (**31**)

From **14**. Red oil; yield 60%. IR (υ̅_max_, in cm^–1^): 3394 (O–H), 2937 (C–H), 1632 (C = C_ar_), 1531; 1381 (NO_2_), 1267 (C–O–C). ^1^H NMR (δ ppm; CDCl_3_, 300 MHz): 7.07 (s; 1H; H-3), 7.55 (s; 1H; H-5), 3.93 (s; 3H; H-7), 2.87–2.73 (m; 2H; H-8a; H-8b), 4.55–4.32 (m; 3H; H-9; H-10a; H-10b), 7.66 (s; 2H; H-11; H-12). ^13^C NMR (δ ppm; CDCl_3_, 75 MHz): 150.1 (C-2), 145.4 (C-6), 133.7 (C-11), 133.6 (C-1), 125.2 (C-12), 119.3 (C-3), 116.0 (C-5), 70.9 (C-9), 56.9 (C-7), 55.1 (C-10), 40.1 (C-8). HRMS (ESI) calcd for C_12_H_14_O_5_N_4_ [M + H]^+^ 295.0964, found 295.1032.

##### 4.1.2.31 (±)-1-(3,4-Dimethoxyphenyl)-3-(1*H*-1,2,3-triazol-1-yl)­propan-2-ol (**32**)

From **15**. Colorless oil;yield 55%. IR (υ̅_max_, in cm^–1^): 3382 (O–H), 2936 (C–H), 1591 (C = C_ar_), 1259 (C–O–C). ^1^H NMR (δ ppm; CDCl_3_, 300 MHz): 6.79–6.71 (m; 3H; H-3; H-5; H-6), 3.82 (s; 3H; H-7), 2.66 (dd; ^3^ *J*_H8a,H9_ = 7.5 Hz, ^2^ *J*_H8a,H8b_ = 13.8 Hz; 1H; H-8a), 2.76 (dd; ^3^ *J*_H8b,H9_ = 5.2 Hz, ^2^ *J*_H8b,H8a_ = 13.8 Hz; 1H; H-8b), 4.30–4.20 (m; 2H; H-9; H-10a), 4.49 (dd; ^3^ *J*_H10a,H9_ = 2.0 Hz, ^2^ *J*_H10a,H10b_ = 12.9 Hz; 1H; H-10b), 7.65 (d; ^3^ *J*_H11,H12_ = 1.0 Hz; 1H; H-11), 3.81 (s; 3H; H-13). ^13^C NMR (δ ppm; CDCl_3_, 75 MHz): 149.0 (C-2), 147.9 (C-1), 133.5 (C-11), 129.2 (C-4), 125.1 (C-12), 121.4 (C-3), 112.4 (C-5), 111.3 (C-6), 71.3 (C-9) 55.9 (C-7), 55.9 (C-13), 54.9 (C-10), 40.5 (C-8). HRMS (ESI) calcd for C_13_H_17_O_3_N_3_ [M + H]^+^ 264.1270, found 264.1337.

##### 4.1.2.32 (±)-1-(3,4-Dimethoxy-5-nitrophenyl)-3-(1*H*-1,2,3-triazol-1-yl)­propan-2-ol (**33**)

From **16**. Orange oil; yield 65%. IR (υ̅_max_, in cm^–1^): 3383 (O–H), 2941 (C–H), 1615 (C = C_ar_), 1531; 1361 (NO_2_), 1281 (C–O–C). ^1^H NMR (δ ppm; CDCl_3_, 300 MHz): 7.05 (dd; ^4^ *J*_3,5_ = 2.0; 1H, H-3), 7.21 (d; ^4^ *J*_5,3_ = 1.8, 1H, H-5), 3.94 (s; 3H, H-7), 2.77 (dd; ^3^ *J*_8a,9_ = 7.2, ^2^ *J*_8a,8b_ = 13.9; 1H, H-8a), 2.87 (dd; ^3^ *J*_8b,9_ = 4.6, ^2^ *J*_8b,8a_ = 14.0; 1H, H-8b), 4.38–4.28 (m; 2H, H-9, H-10a), 4.53 (dd; ^3^ *J*_10b,9_ = 7.6, ^2^ *J*_10b,10a_ = 10.6; 1H, H-10b), 7.68–7.62 (m; 2H, H-11, H-12), 3.91 (s; 3H, H-13). ^13^C NMR (δ ppm; CDCl_3_, 75 MHz): 141.4 (C-1), 153.9 (C-2), 116.4 (C-3), 133.7 (C-4), 117.6 (C-5), 144.5 (C-6), 56.5 (C-7), 40.3 (C-8), 70.8 (C-9), 55.1 (C-10), 133.4 (C-11), 130.7 (C-12), 62.0 (C-13). HRMS (ESI) calcd for C_13_H_16_O_5_N_4_ [M + H]^+^ 309.1121, found 309.1188.

##### 4.1.2.33 (±)-1-(4-(Benzyloxy)-3-methoxy-5-nitrophenyl)-3-(1*H*-1,2,3-triazol-1-yl)­propan-2-ol (**34**)

From **17**. Brown semisolid, yield 58%. IR (υ̅_max_, in cm^–1^): 3302 (O–H), 2917 (C–H), 1612 (C = C_ar_), 1223 (C–O–C), 1530; 1357 (NO_2_). ^1^H NMR (δ ppm; CDCl_3_, 300 MHz): 6.45 (d; ^4^ *J*_3,5_ = 2.0; 1H, H-3), 7.09 (d; 1H, H-5), 3.33 (s; 3H, H-7), 2.14 (dd; ^3^ *J*_8a,9_ = 7.6, ^2^ *J*_8a,8b_ = 14.0; 1H, H-8a), 2.25 (dd; ^3^ *J*_8b,9_ = 4.0, ^2^ *J*_8b,8a_ = 13.6; 1H, H-8b), 3.11–3.05 (m; 2H, H-9, H-10a), 3.95 (m; 1H, H-10b), 6.90–6.71 (m; 7H, H-11, H-12, H-14, H-15, H-16, H-17), 4.53 (s; 2H, H-13). ^13^C NMR (δ ppm; CDCl_3_, 75 MHz): 154.3 (C-2), 145.1 (C-6), 140.3 (C-1), 136.4 (C-14), 133.4 (C-4), 129.7 (C-11), 128.6 (C-16), 128.5 (C-17), 128.4 (C-15), 117.4 (C-3), 116.5 (C-5), 76.0 (C-13), 70.8 (C-9), 56.5 (C-7), 54.9 (C-10), 40.3 (C-8). HRMS (ESI) calcd for C_19_H_20_O_5_N_4_ [M + H]^+^ 385.1434, found 385.21.

### 4.2 Chiral Resolution

The enantiomers **(+)-29** and **(−)-29** were resolved by high-performance liquid chromatography (HPLC) using chiral stationary phase (CSP) analytical columns based on derivatized polysaccharides. All solvents used were HPLC-grade. Mobile phases were prepared using mixtures of *n*-hexane and ethanol in specified volume ratios and degassed in an ultrasonic bath for 15 min. Sample solutions were filtered through 0.45 μm Durapore-GV membranes (Millipore) prior to injection. Chromatographic analyses were carried out at room temperature on analytical chiral columns (25 × 0.46 or 15 × 0.46 cm), packed with amylose or cellulose tris­(3,5-dimethylphenylcarbamate) as the chiral selector. The flow rate was set to 0.8 mL·min^–1^, and detection was performed at 260 nm, selected based on UV absorption scans. Before analysis, columns were conditioned overnight with absolute ethanol at a flow rate of 0.4 mL·min^–1^. The retention factor (*k*) was calculated using the equation: *k* = *t*_R_ – *t*_0_/*t*_0_; where *t*_0_ is the retention time of the solvent front, and *t*_R_ is the retention time of the analyte. Enantioselectivity (α) was determined as α = *k*_2_/*k*_1_; and resolution (*R*_s_) was calculated by *R*_s_ = 2­(*t*_R2_ – *t*_R1_)*W*_1_ + *W*_2_; where *t*_R_ and *W* correspond to the retention times and baseline peak widths of the enantiomers, respectively.

For preparative purposes, fractions were collected manually from the UV/vis detector outlet, following disconnection of the polarimeter. A timing correction of approximately 30 s was applied to align detector response with outlet elution, enabling precise determination of collection windows. The collected fractions were concentrated under reduced pressure at room temperature and subsequently analyzed for enantiomeric excess. Optical rotations were measured at 22.8 °C using a PerkinElmer 241 polarimeter equipped with a sodium lamp and a 10 dm path length cell. Specific rotation was calculated as [α]_D_ = α × 100*l* × *c*; where α is the observed rotation, *l* is the path length in decimeters, and *c* is the sample concentration in g·mL^–1^.

### 4.3 Biological Studies

Stock solutions of hybrids and benznidazole were prepared in a water/dimethyl sulfoxide (DMSO) mixture in concentrations around 50 to 100 mmol L^–1^.

#### 4.3.1 Evaluation of Anti-*T. cruzi* Activity

The *T. cruzi* Y strain was selected for this study due to its known partial sensitivity to benznidazole chemotherapy.^69^

##### 4.3.1.1 Evaluation against Trypomastigote Forms

Trypomastigote forms of *T. cruzi* (2.5 ×  10^5^ parasites/well) were incubated in 96-well plates with serial dilutions of the hybrid compounds (100, 50, 25, and 12.5 μmol·L^–1^) for 24 h at 37 °C in a humidified 5% CO_2_ atmosphere. Following incubation, parasite viability was assessed under an optical microscope by evaluating motility, and the number of live parasites was quantified using a Neubauer chamber. Untreated parasite cultures were maintained in parallel as negative controls. The mortality percentage was calculated by comparing the number of live parasites in treated wells to that of untreated controls. All experiments were conducted in triplicate with at least two technical replicates per concentration. EC_50_ values were determined by nonlinear regression using GraphPad Prism 8 and CompuSyn software (Biosoft, U.K.).

##### 4.3.1.2 Evaluation against Amastigote Forms^50,51^

Vero or H9c2 cells (7.5 × 10^3^ cells/well) were seeded on sterile glass coverslips placed in 24-well plates and incubated for 24 h at 37 °C in 5% CO_2_ to allow cell adhesion. Following this period, cells were infected with trypomastigote forms of *T. cruzi* Y strain at a ratio of 20 parasites per cell. After 24 h of parasite-host interaction, noninternalized parasites were removed by washing with fresh medium, and the infected monolayers were incubated for 48 h with the hybrid compounds at a fixed concentration of 50 μmol·L^–1^, following toxicity parameters. Infected but untreated cells were included as negative controls in all experiments. At the end of the treatment period, coverslips were washed with medium, fixed with methanol, and stained with Giemsa solution. After drying, the coverslips were mounted onto microscope slides using Entellan (Merck) and analyzed under a light microscope at 100× magnification. At least 200 cells were counted per coverslip, and only intracellular parasite forms with characteristic morphology were considered for analysis. The infection index was determined by the percentage of infected cells relative to the total number of cells analyzed. The inhibition percentage was calculated based on the reduction in infection in treated samples compared to the untreated control. Dose–response curves were constructed using GraphPad Prism 8 and CompuSyn (Biosoft, U.K.). All experiments were performed in triplicate, with at least two technical replicates per condition.

#### 4.3.2 Cell Viability Assay^50,70^

Vero and H9c2 cells (2 × 10^3^ cells/well) were seeded in 96-well plates in Dulbecco’s modified Eagle’s medium (DMEM) supplemented with 5% fetal bovine serum (FBS) and incubated for 24 h at 37 °C in a humidified atmosphere with 5% CO_2_. After incubation, the medium was replaced with fresh DMEM + 5% FBS containing serial dilutions of each hybrid compound (1400 to 175 μmol·L^–1^). Cells were exposed to the compounds for 48 h. Four wells with untreated cells served as a positive control. Additionally, eight wells without cells were maintained as negative controls: four containing DMEM + 5% FBS + resazurin (1 mmol·L^–1^), and four containing only DMEM + 5% FBS. After the 48-h incubation, the treatment medium was removed and replaced with DMEM + 5% FBS containing 10% resazurin solution (1 mmol·L^–1^). Plates were incubated for an additional 6 h, and then absorbance was measured at 570 and 600 nm using a microplate reader. Dose–response curves were constructed using GraphPad Prism 8 software, and CC_50_ values were determined by nonlinear regression.

### 4.4 Statistical Analysis

Statistical analyses were performed using the *t*-test. Differences were considered statistically significant when the probability of error was less than 5% (*p* < 0.05).

## Supplementary Material



## Notes

Structural characterization data (Infrared, ^1^H, and ^13^C NMR and mass spectra for all new compounds (PDF)
