# Supplementary material for: Design, Synthesis, and Trypanosomicidal Evaluation of Eugenol-Based Azole Hybrids: Discovery of an In Vitro Active and Selective Compound
Source: ACS Omega. 2025 Aug 29;10(36):41958–74. doi: 10.1021/acsomega.5c06619 (PMC12444550; doi:10.1021/acsomega.5c06619)
Supplement: Supplementary file 1 [file ao5c06619_si_002.pdf]

SUPPLEMENTARY DATA

Design, synthesis, and trypanosomicidal evaluation of eugenol-based azole hybrids: discovery of an *in vitro* active and selective compound

José Vaz Cardoso Machado[a], Clara Oliveira de Carvalho Lopes[a], Sarah Ferreira Maciel[a], Valquíria Ângelis Fernandes[a], Sara Manuela Mendonça da Silva Cravo[b], Maria Emília da Silva Pereira de Sousa[b], Maria Elizabeth Tiritan[b], Thiago Belarmino de Souza[c], Lucas Lopardi Franco[a], Lívia de Figueiredo Diniz[d], Diogo Teixeira Carvalho[a*]

[a]Faculdade de Ciências Farmacêuticas, Universidade Federal de Alfenas, MG, 37130-001, Brazil
[b]Faculdade de Farmácia, Universidade do Porto, 4099-002, Porto, Portugal
[c]Escola de Farmácia, Universidade Federal de Ouro Preto, 35400-000, MG, Brazil
[d]Instituto de Ciências Biomédicas, Universidade Federal de Alfenas, MG, 37130-001, Brazil

**Corresponding author: Diogo Teixeira Carvalho. Faculdade de Ciências Farmacêuticas, Universidade Federal de Alfenas, Rua Gabriel Monteiro da Silva, 700, sala D-208e, Alfenas, MG, 37130-001, Brazil. E-mail: diogo.carvalho@unifal-mg.edu.br. Phone number: +55 35 3701-9213*

INFRARED, NMR AND MASS SPECTRA

OF SYNTHESIZED COMPOUNDS

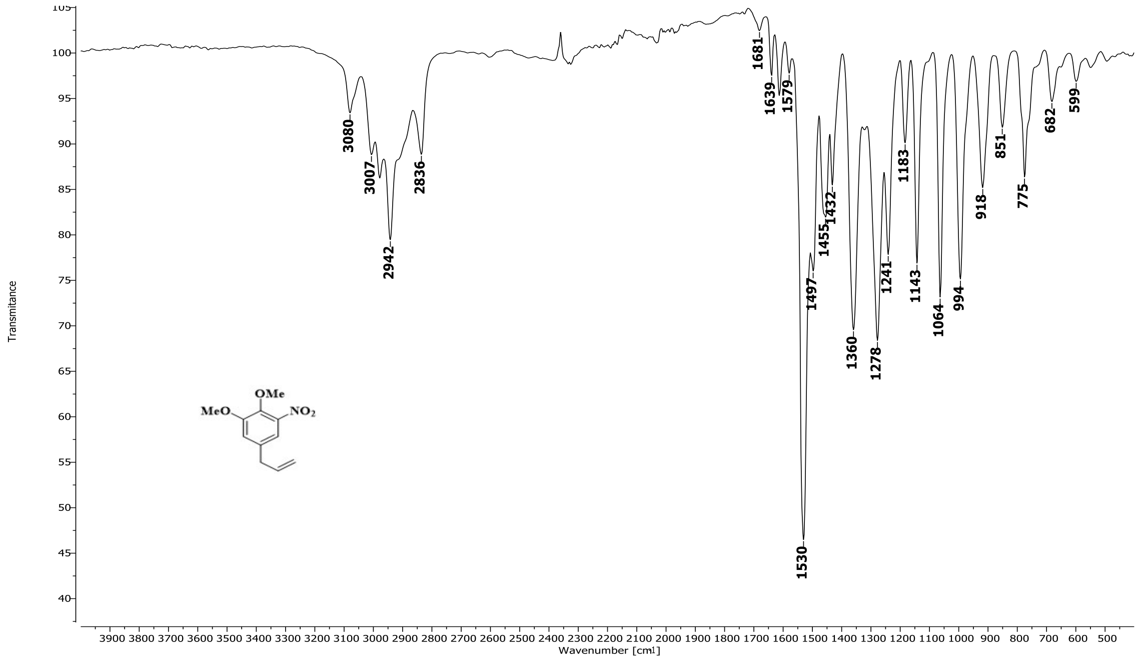

Figure 1: IR spectrum of compound **4**

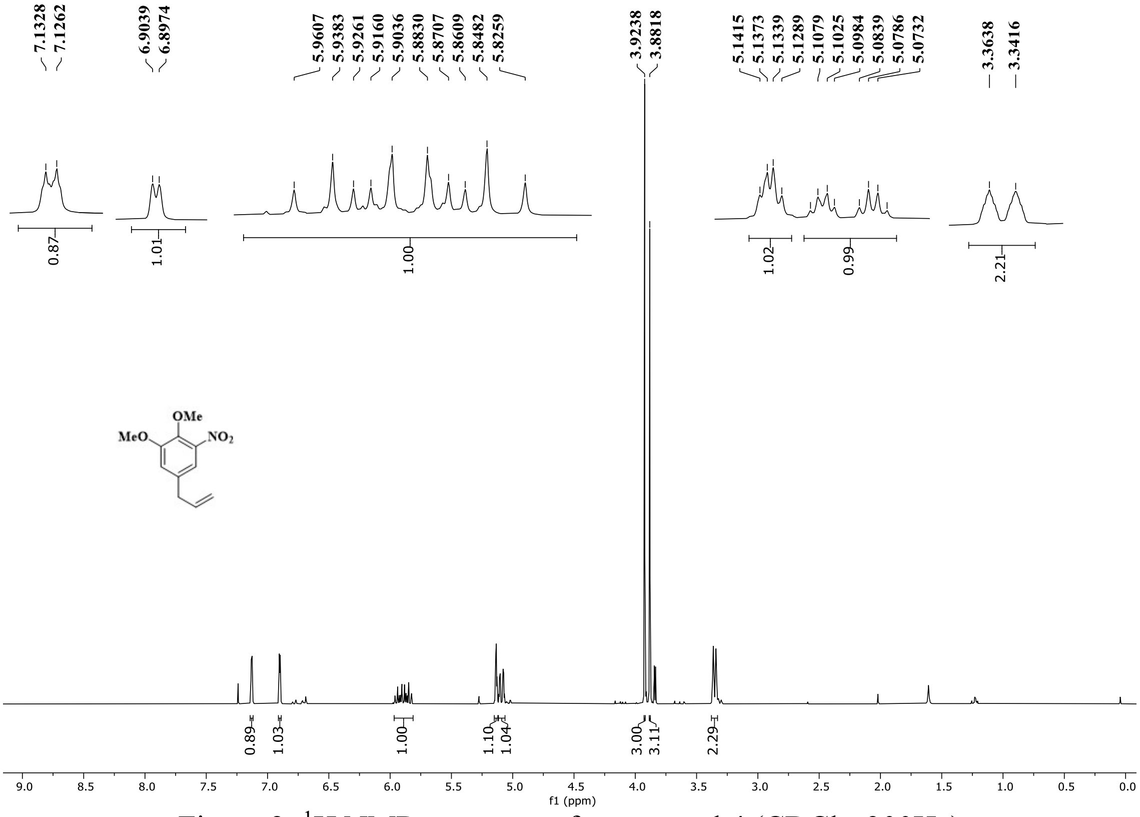

Figure 2: ^{1}H NMR spectrum of compound **4** ($CDCl_3$, 300Hz)

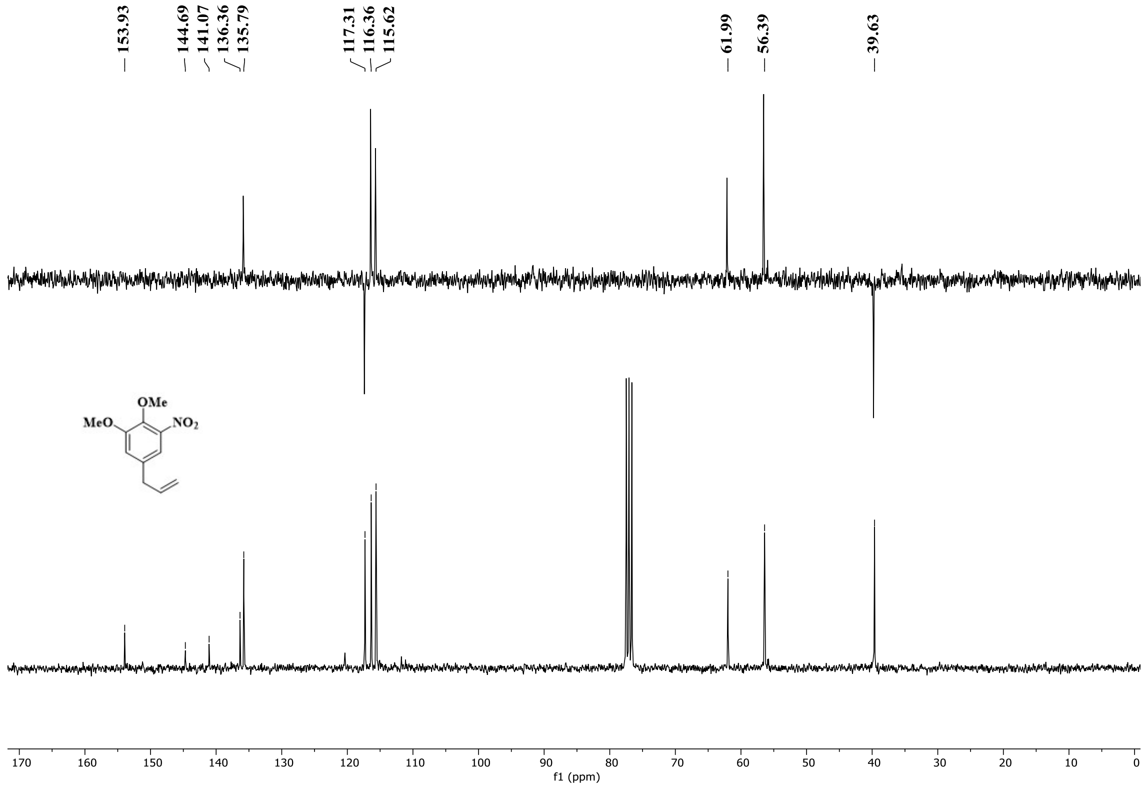

Figure 3: ^{13}C NMR spectrum and DEPT-135 of compound **4** ($CDCl_3$, 75Hz)

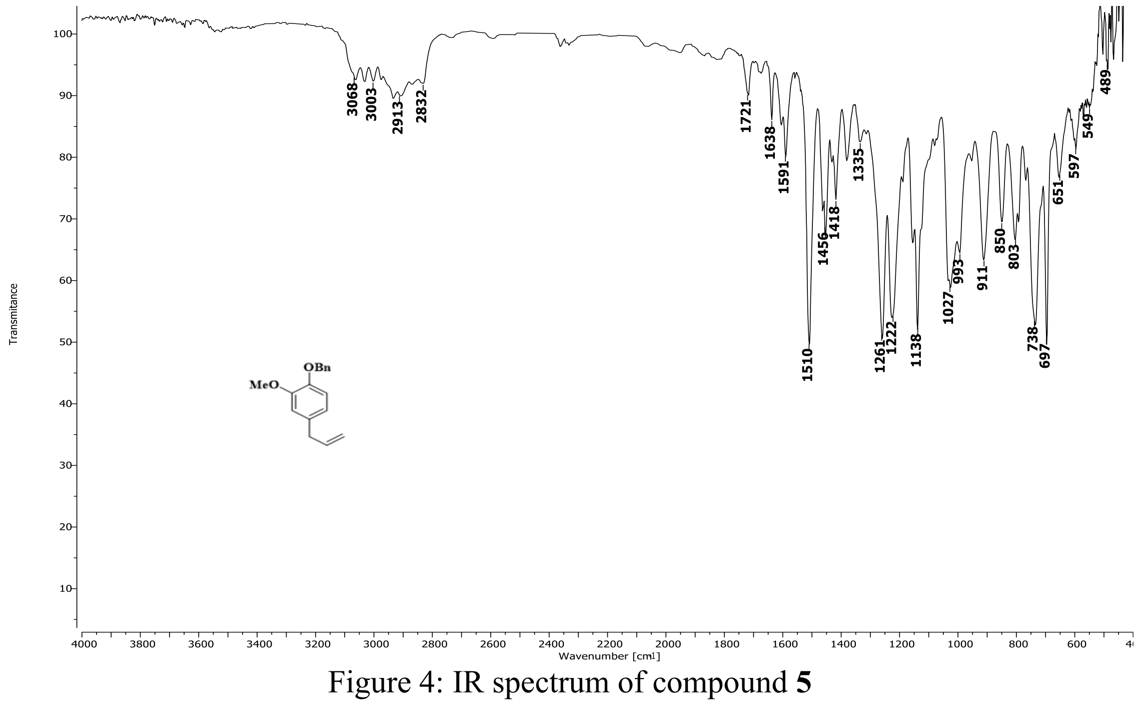

Figure 4: IR spectrum of compound **5**

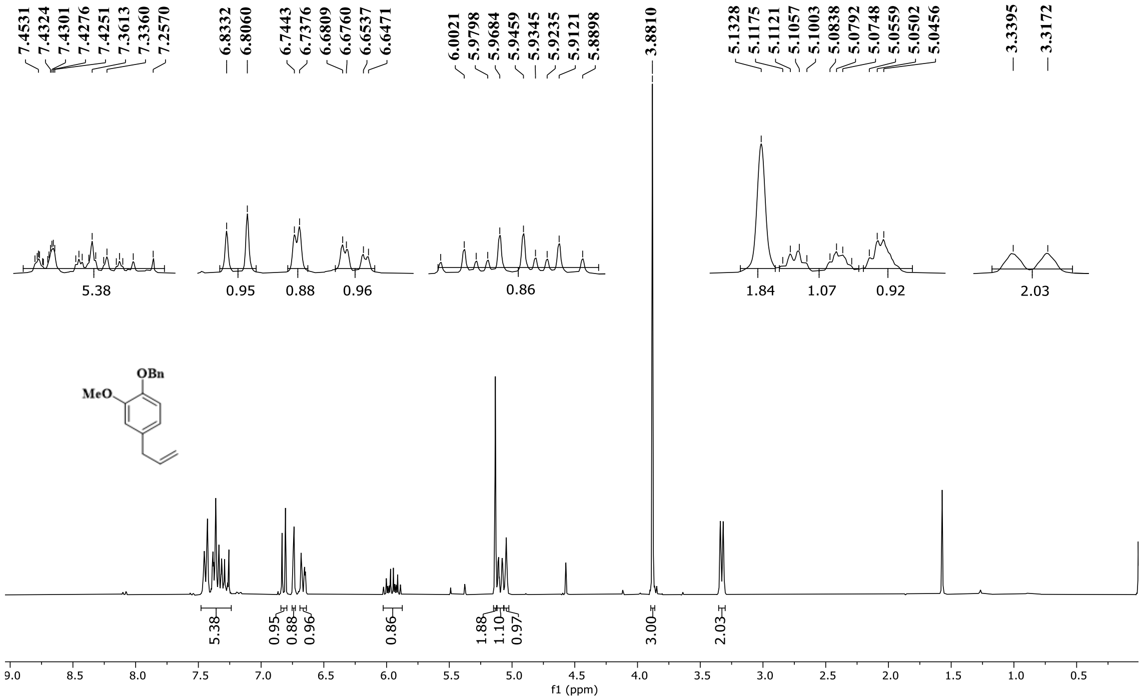

Figure 5: ^{1}H NMR spectrum of compound **5** ($CDCl_3$, 300Hz) NMR

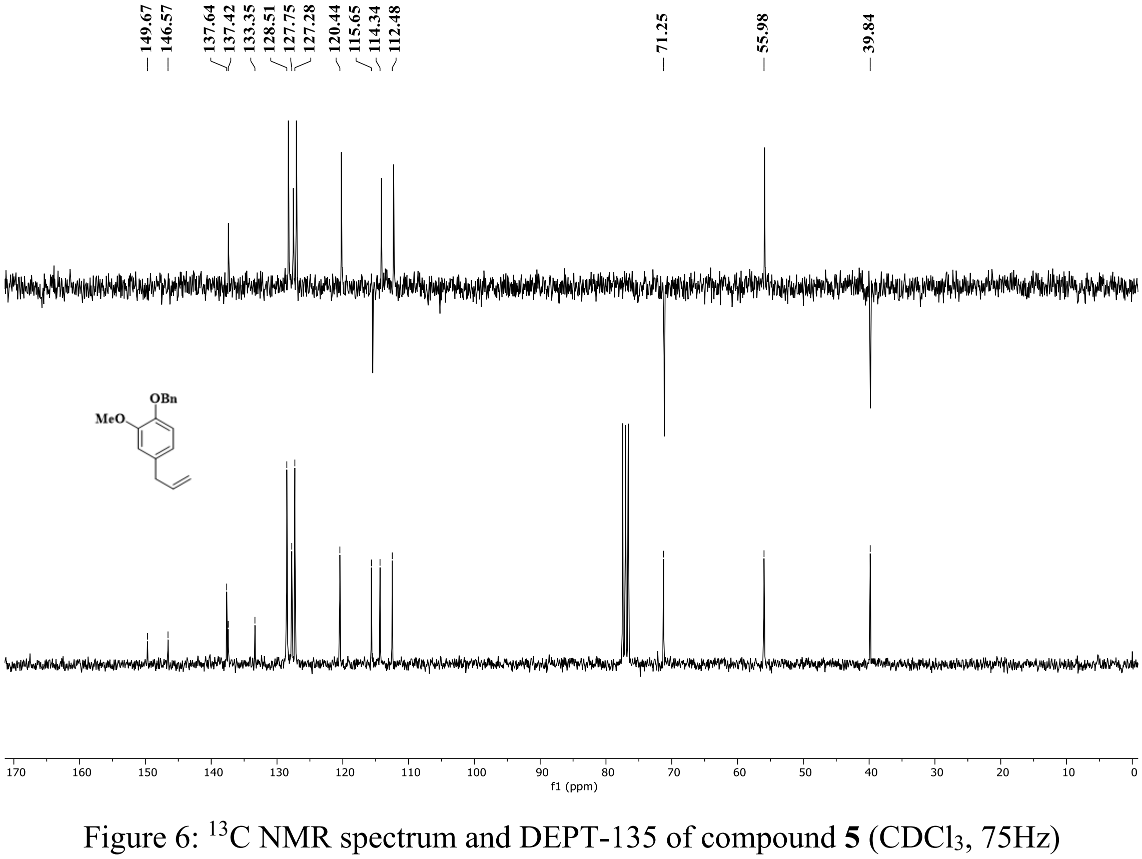

Figure 6: ^{13}C NMR spectrum and DEPT-135 of compound **5** ($CDCl_3$, 75Hz)

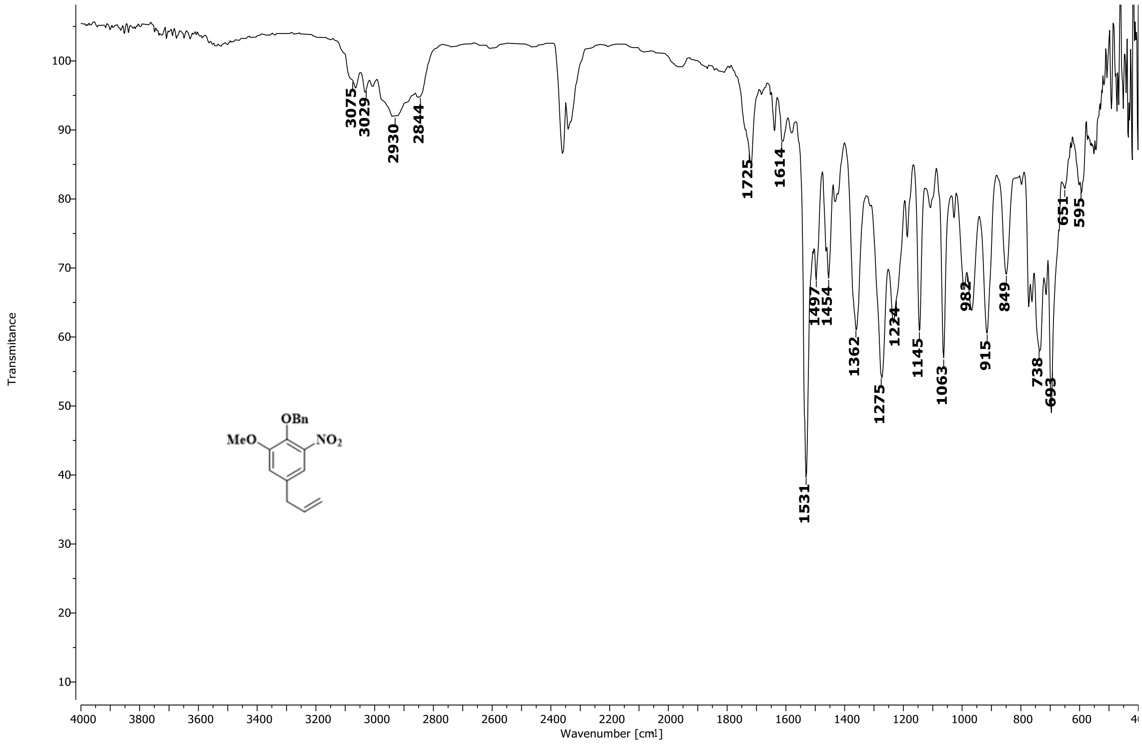

Figure 7: IR spectrum of compound **6**

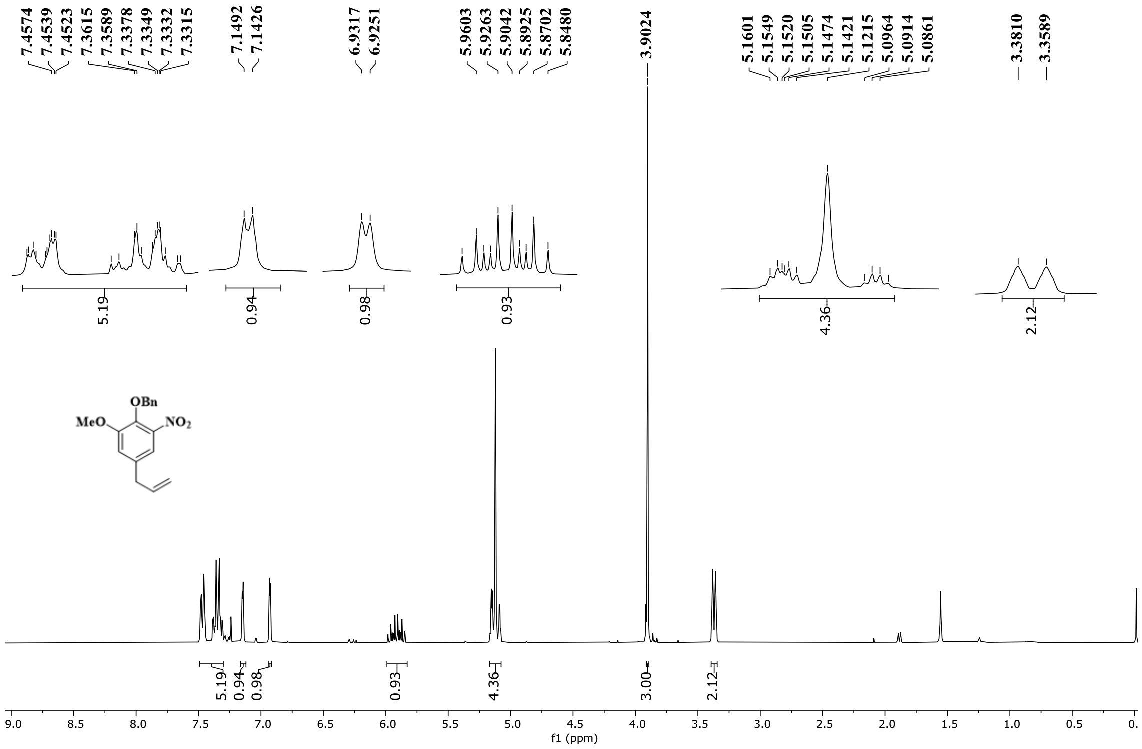

Figure 8: ^{1}H NMR spectrum of compound **6** (CDCl$_3$, 300Hz)

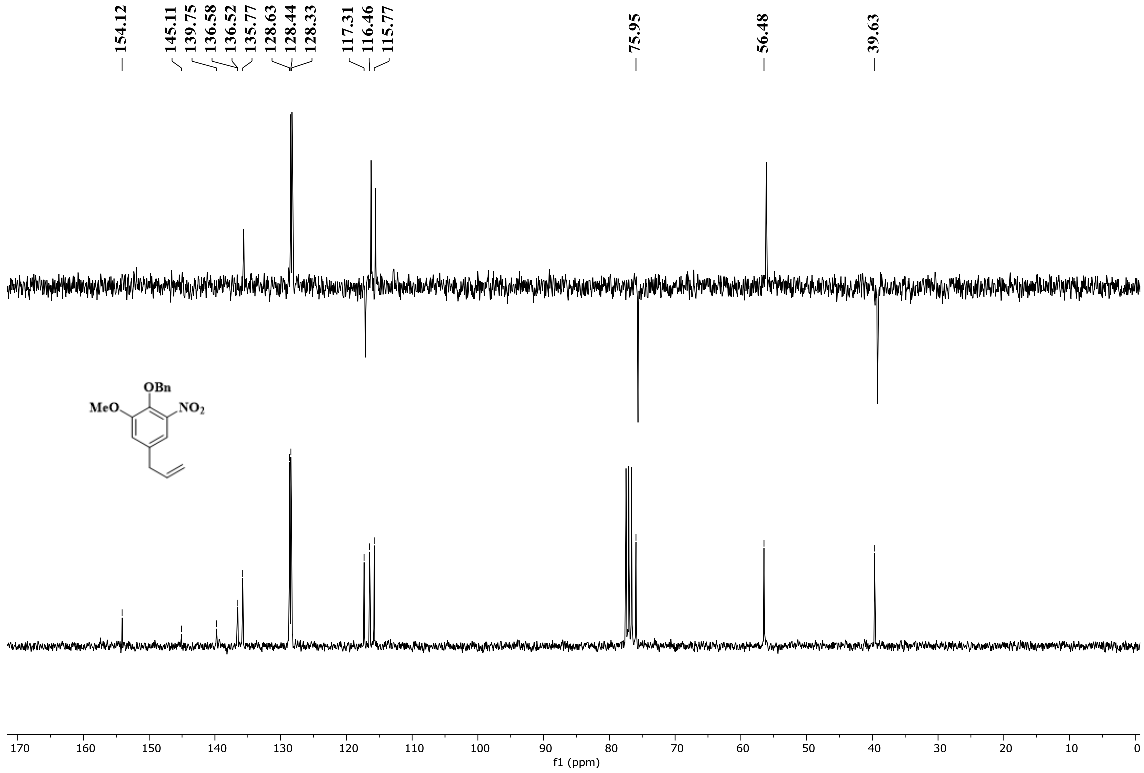

Figure 9: ^{13}C NMR spectrum and DEPT-135 of compound **6** ($CDCl_3$, 75Hz)

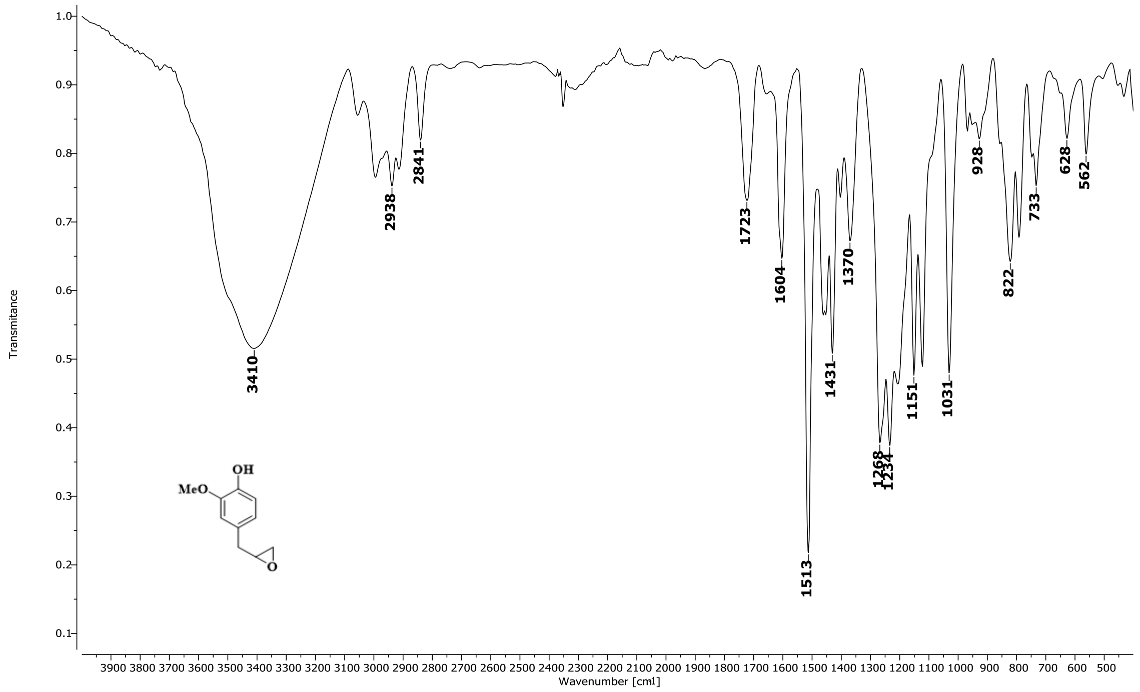

Figure 10: IR spectrum of compound **7**

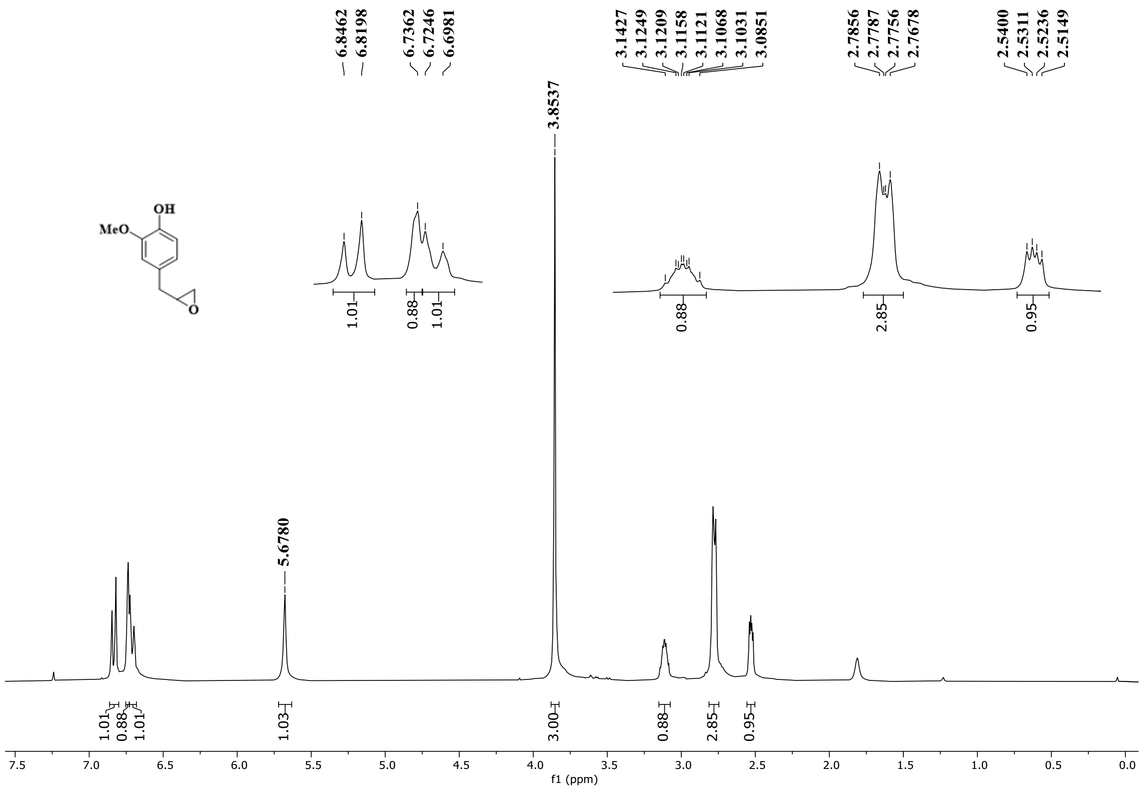

Figure 11: ^{1}H NMR spectrum of compound **7** ($CDCl_3$, 300Hz)

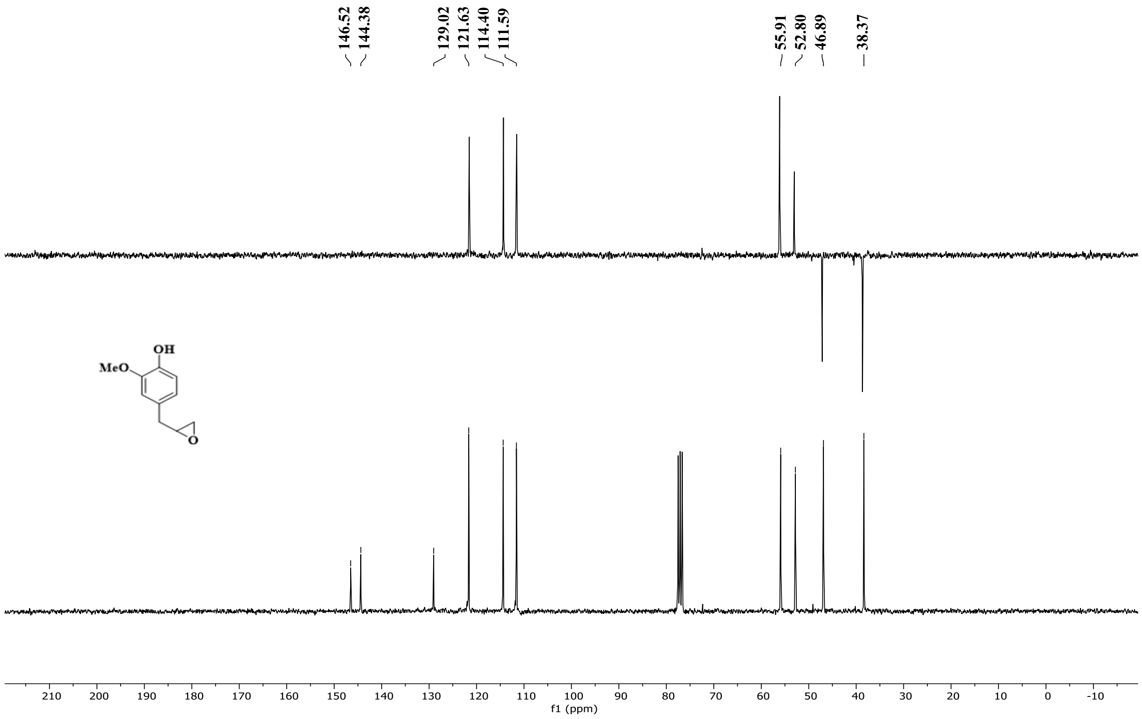

Figure 12: ^{13}C NMR spectrum and DEPT-135 of compound **7** ($CDCl_3$, 75Hz)

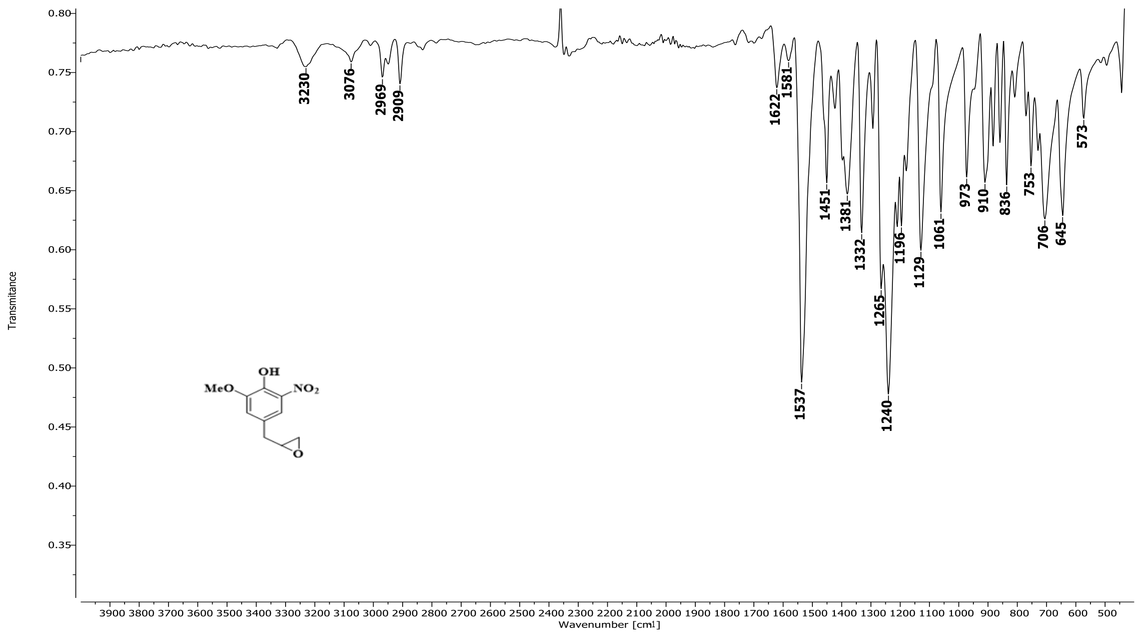

Figure 13: IR spectrum of compound **8**

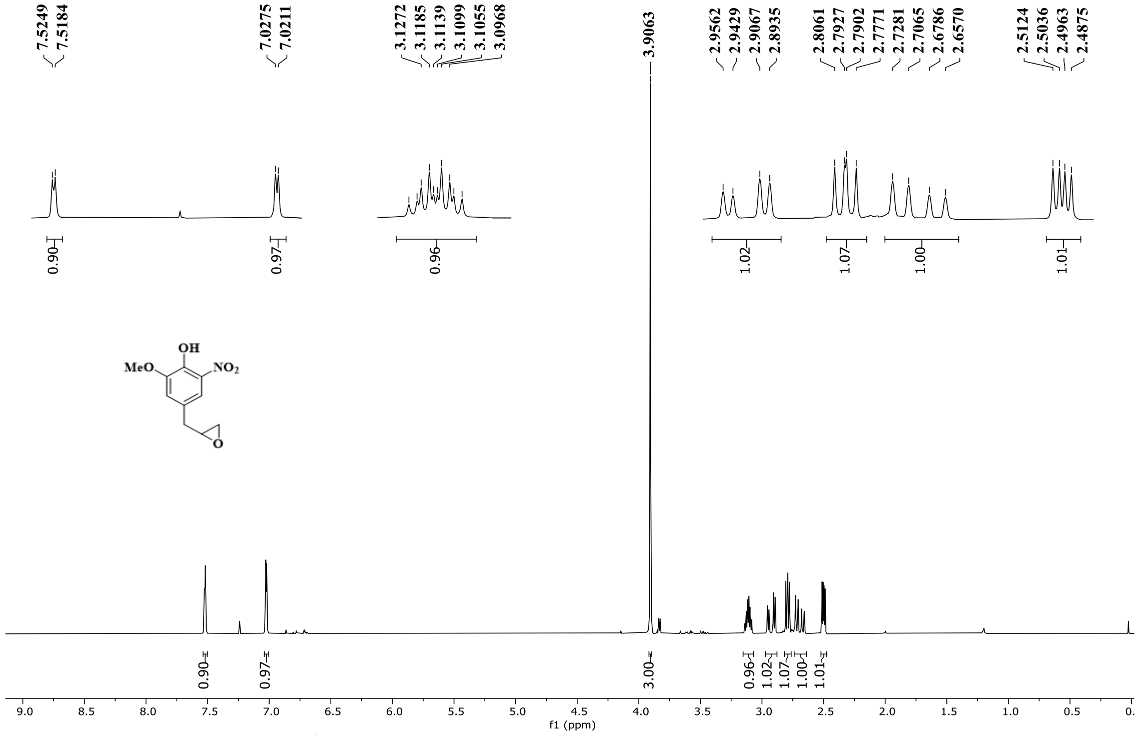

Figure 14: ^{1}H NMR spectrum of compound **8** ($CDCl_3$, 300Hz)

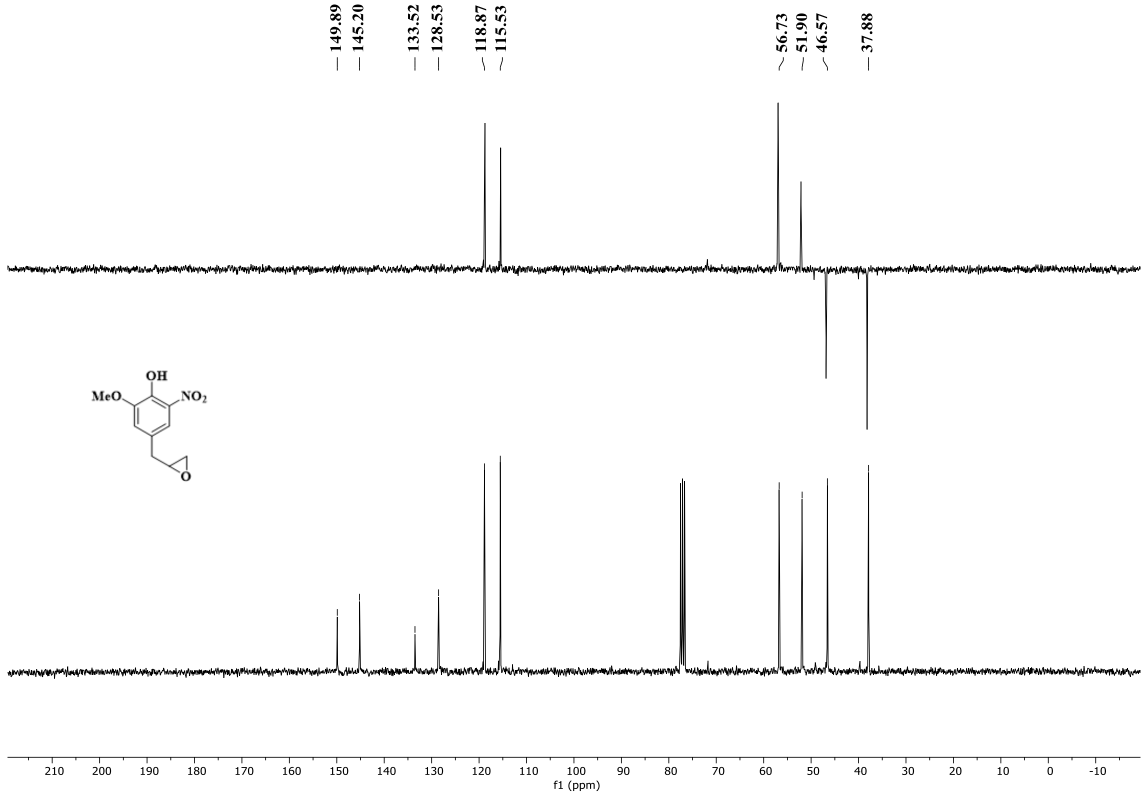

Figure 15: ^{13}C NMR spectrum and DEPT-135 of compound **8** ($CDCl_3$, 75Hz)

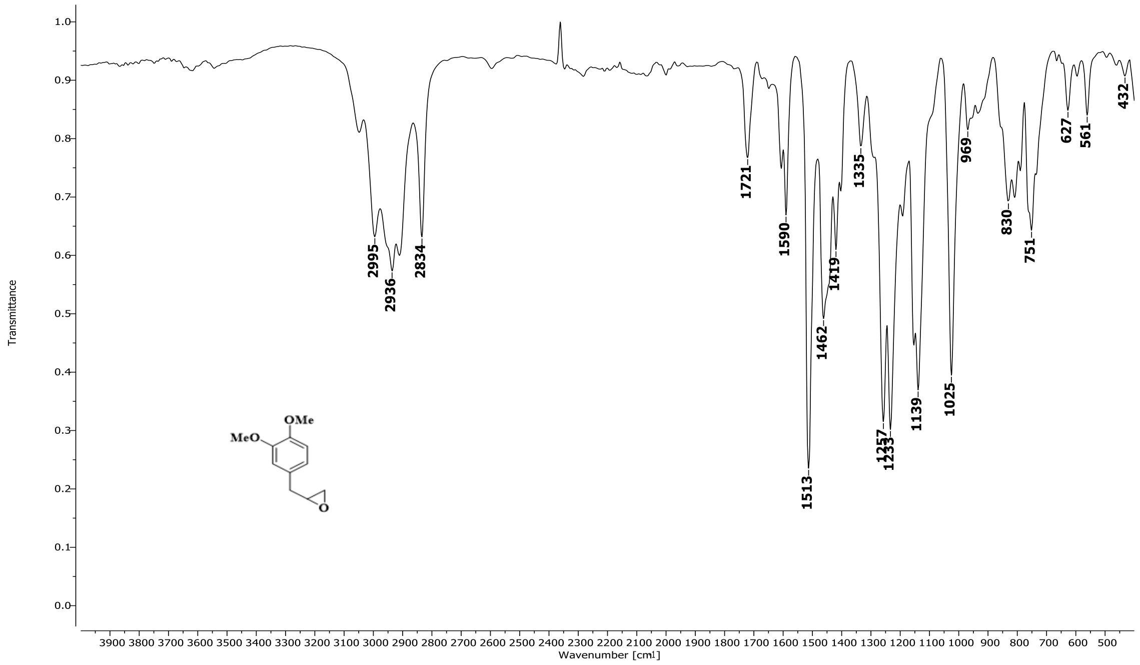

Figure 16: IR spectrum of compound **9**

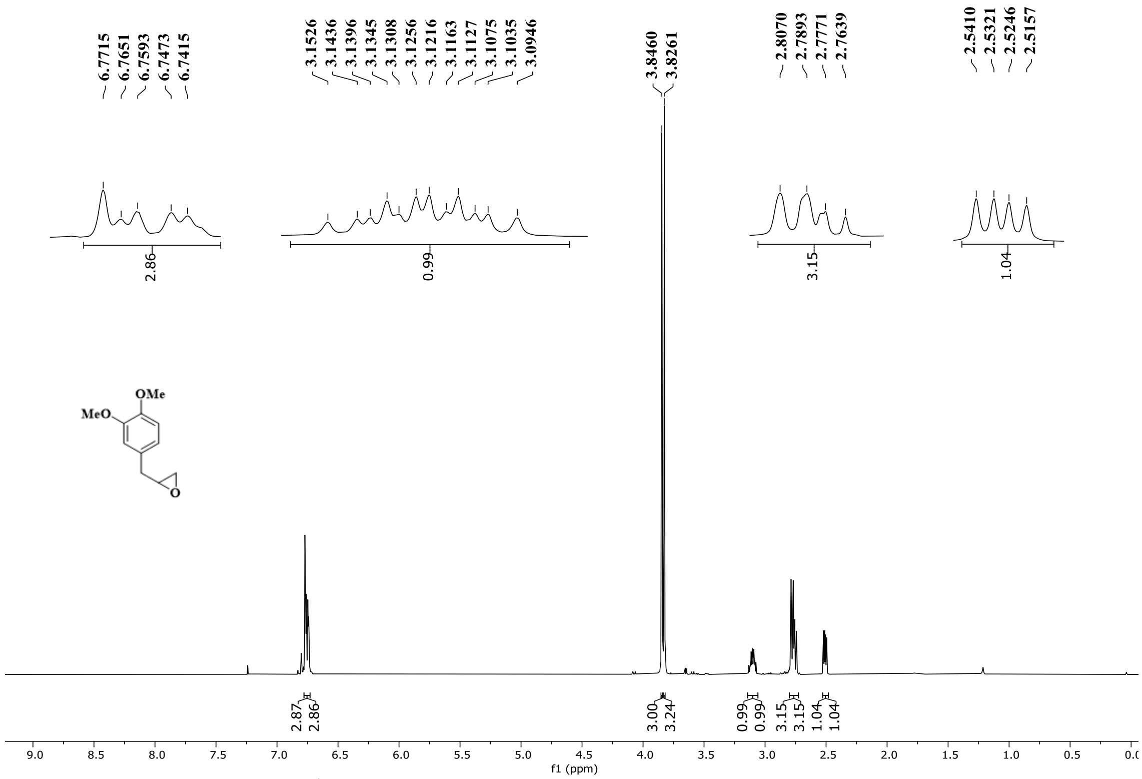

Figure 17: ^{1}H NMR spectrum of compound **9** ($CDCl_3$, 300Hz)

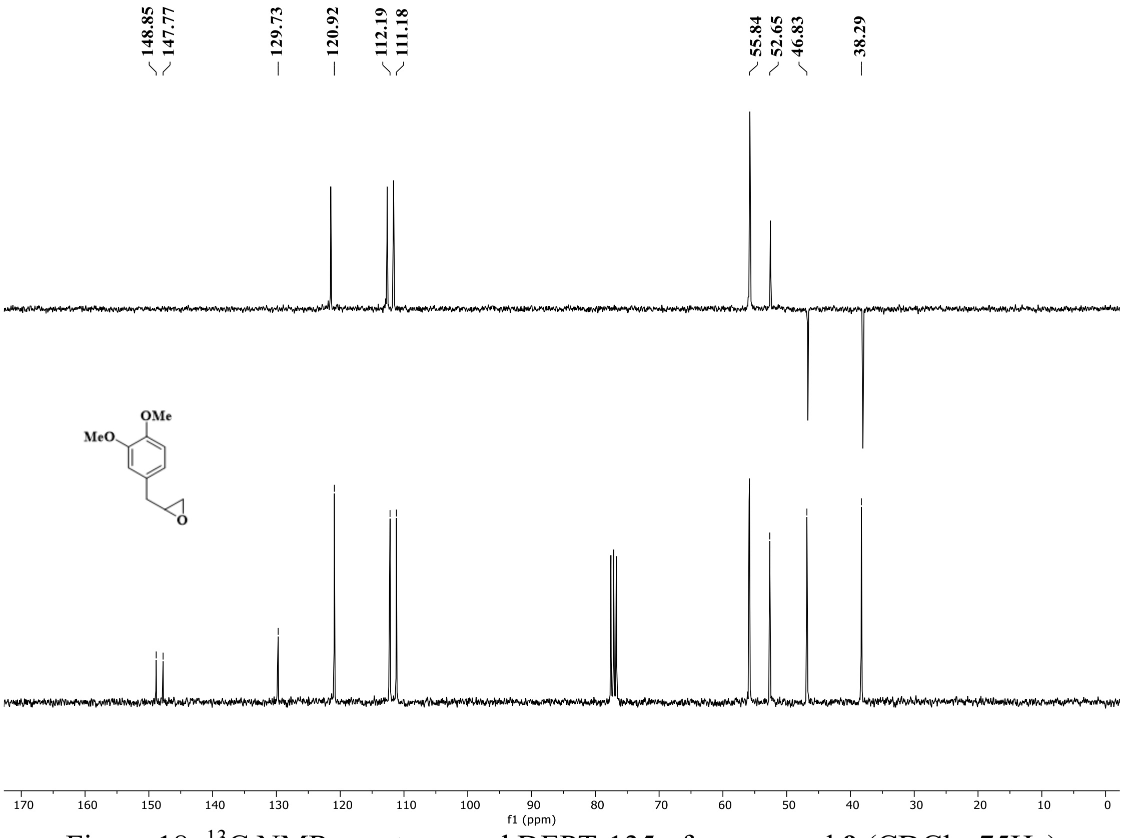

Figure 18: ^{13}C NMR spectrum and DEPT-135 of compound **9** ($CDCl_3$, 75Hz)

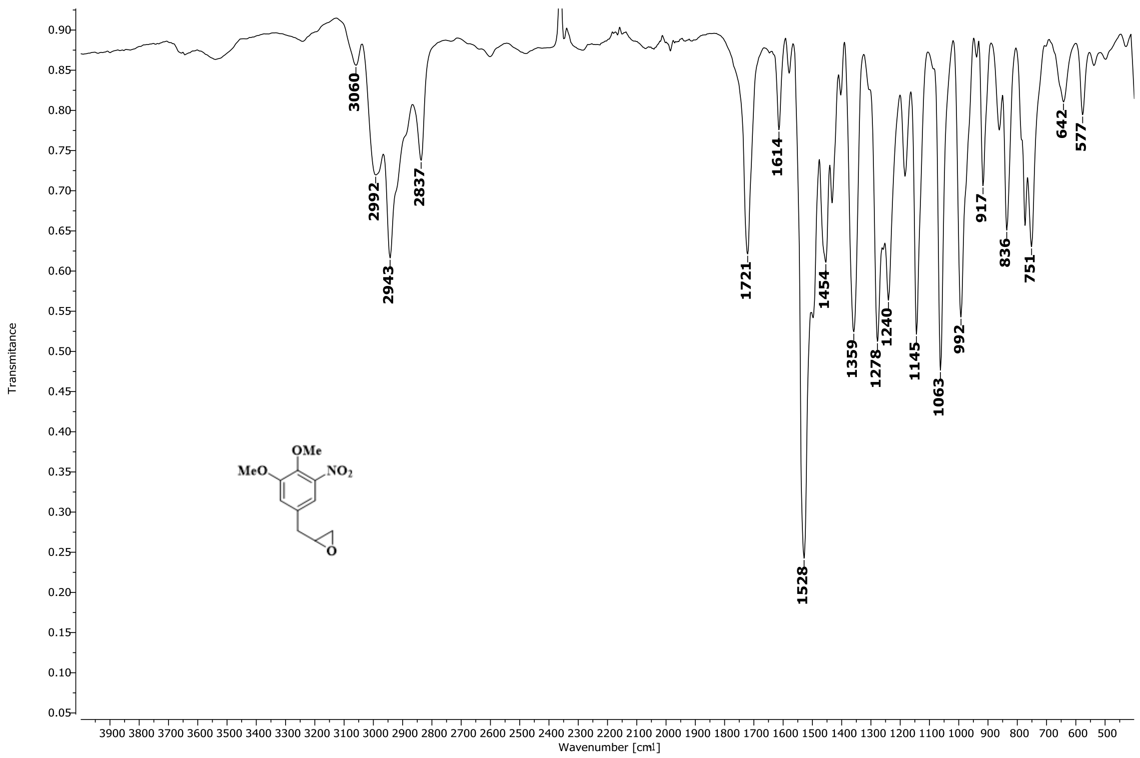

Figure 19: IR spectrum of compound **10**

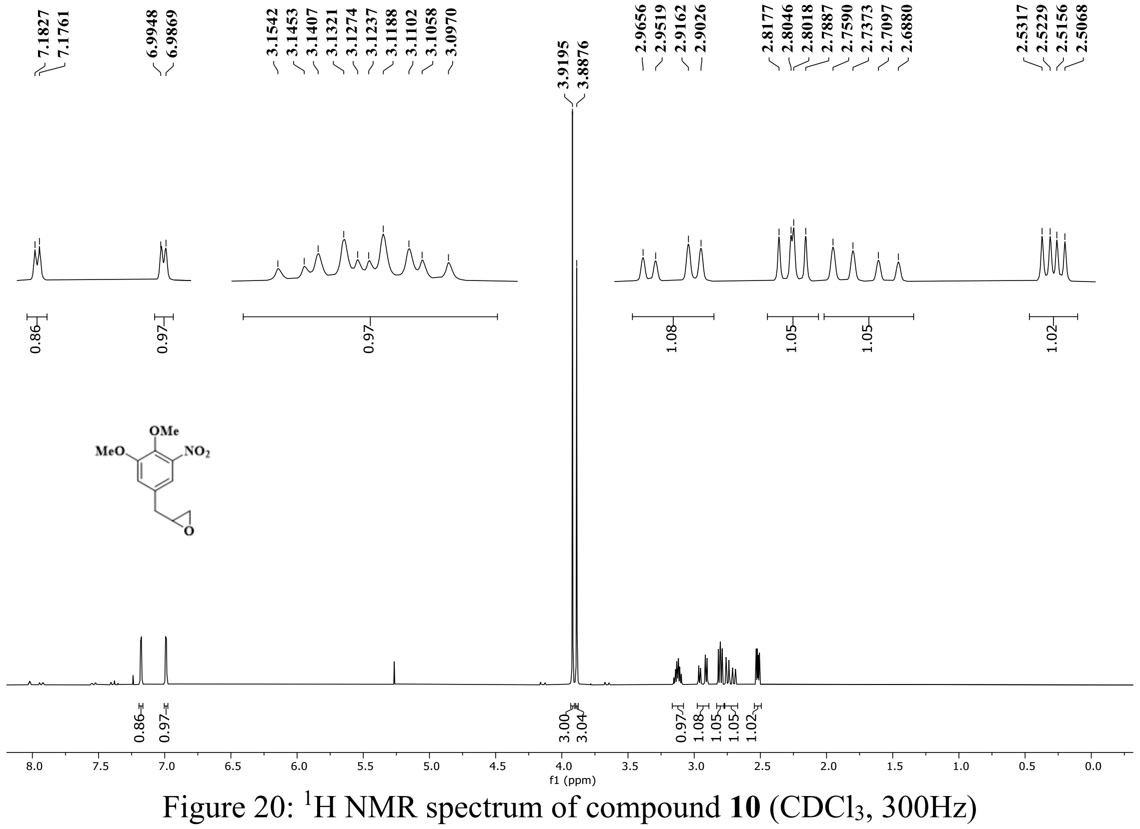

Figure 20: ^{1}H NMR spectrum of compound **10** ($CDCl_3$, 300Hz)

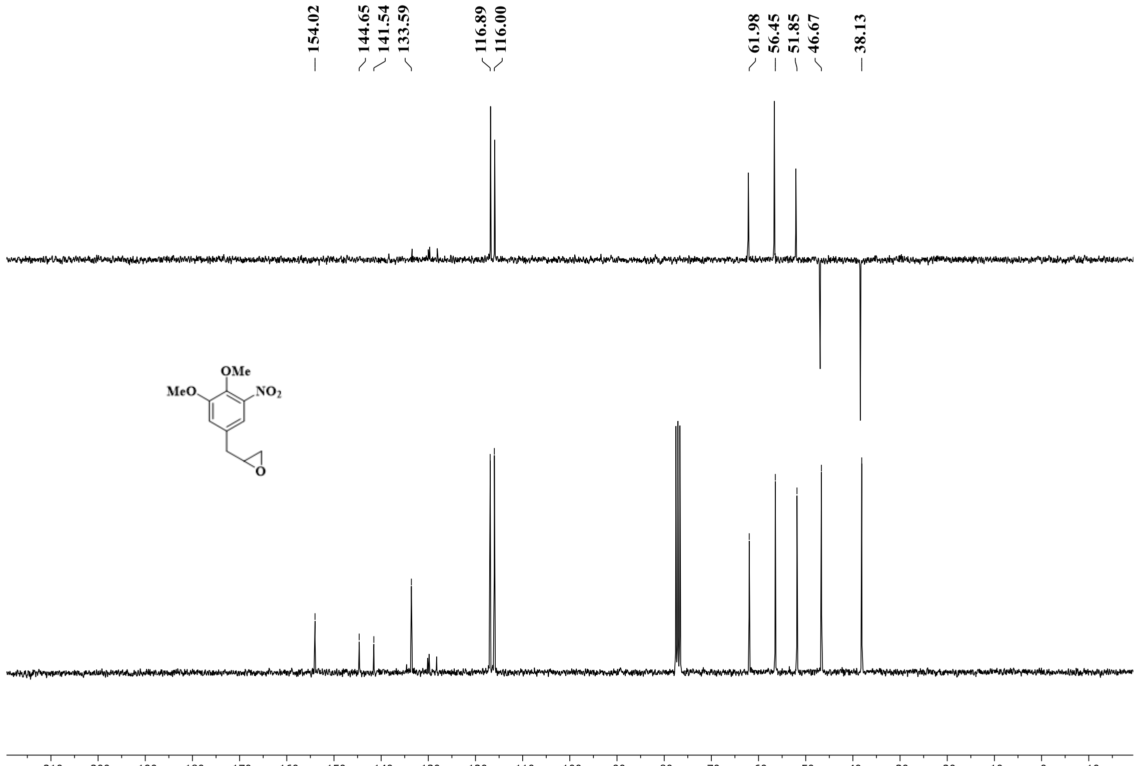

Figure 21: ^{13}C NMR spectrum and DEPT-135 of compound **10** ($CDCl_3$, 75Hz)

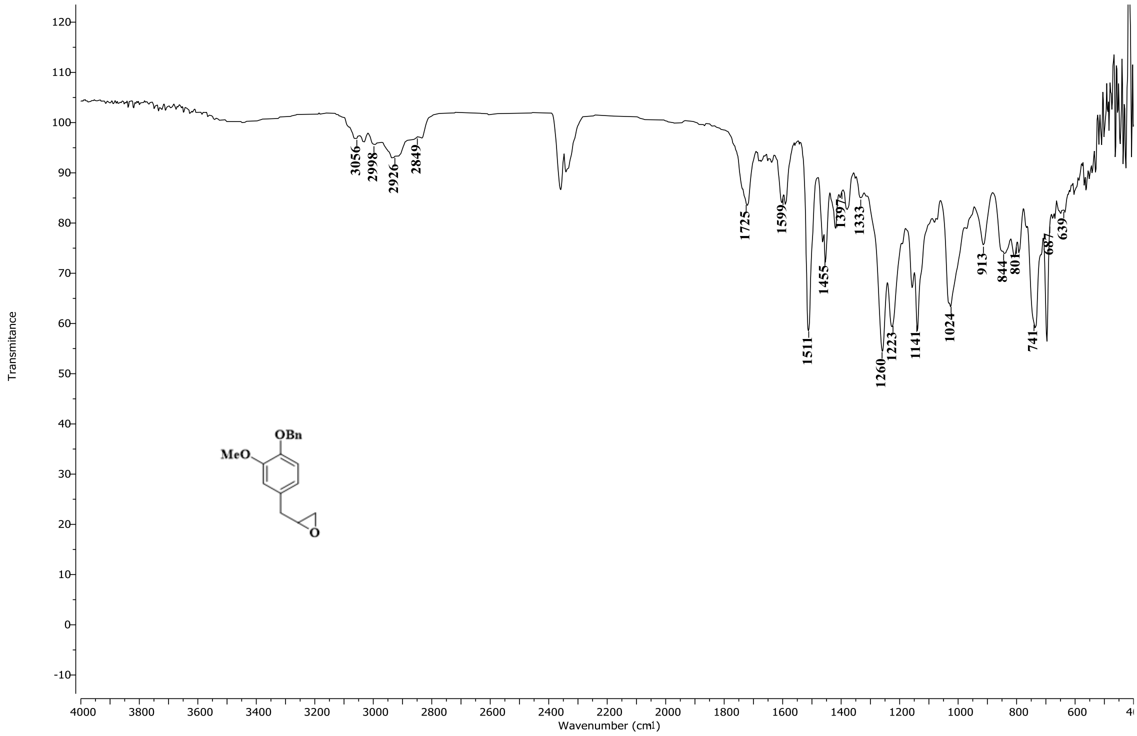

Figure 22: IR spectrum of compound **11**

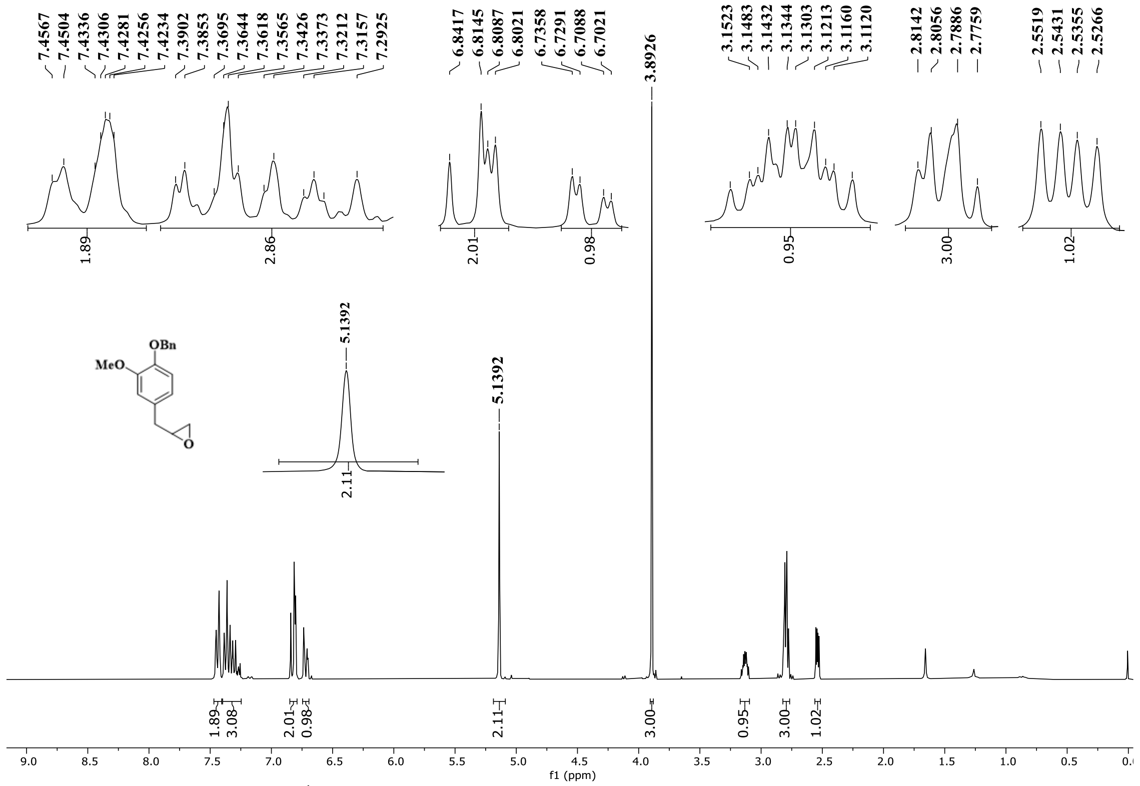

Figure 23: ^{1}H NMR spectrum of compound **11** ($CDCl_3$, 300Hz)

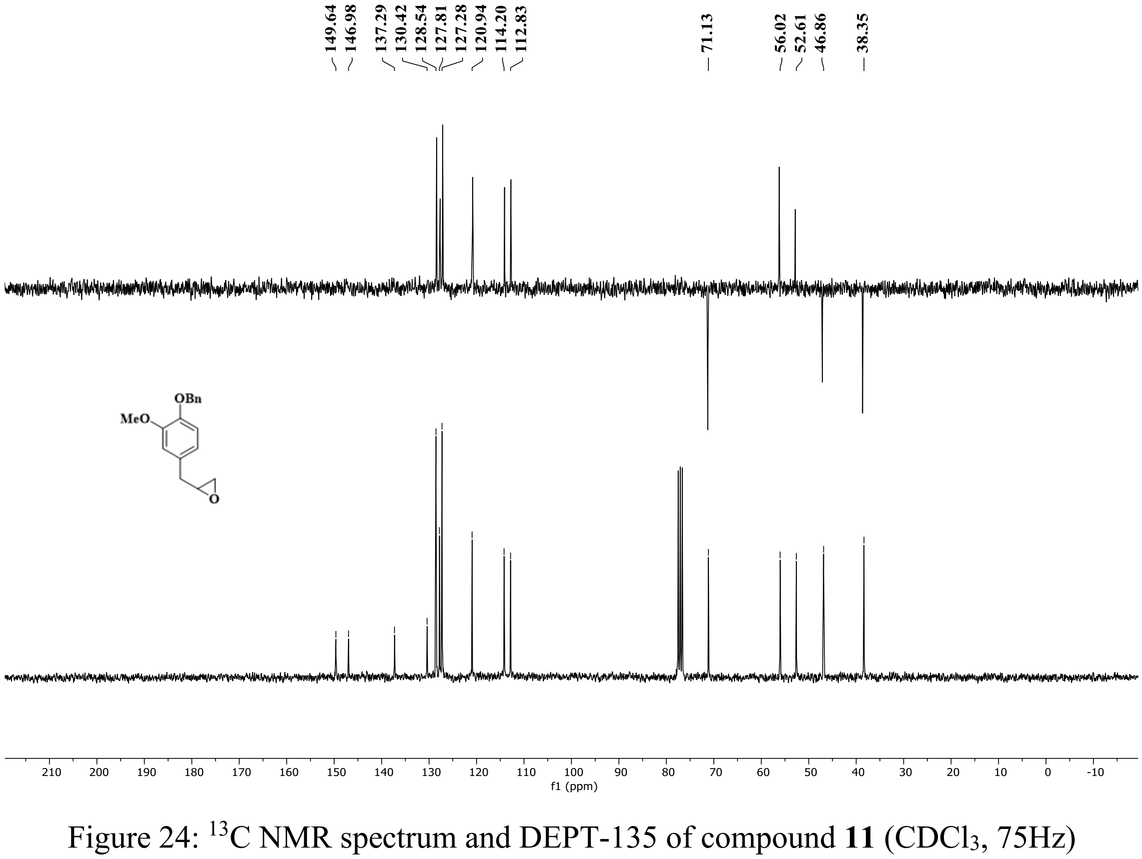

Figure 24: ^{13}C NMR spectrum and DEPT-135 of compound **11** ($CDCl_3$, 75Hz)

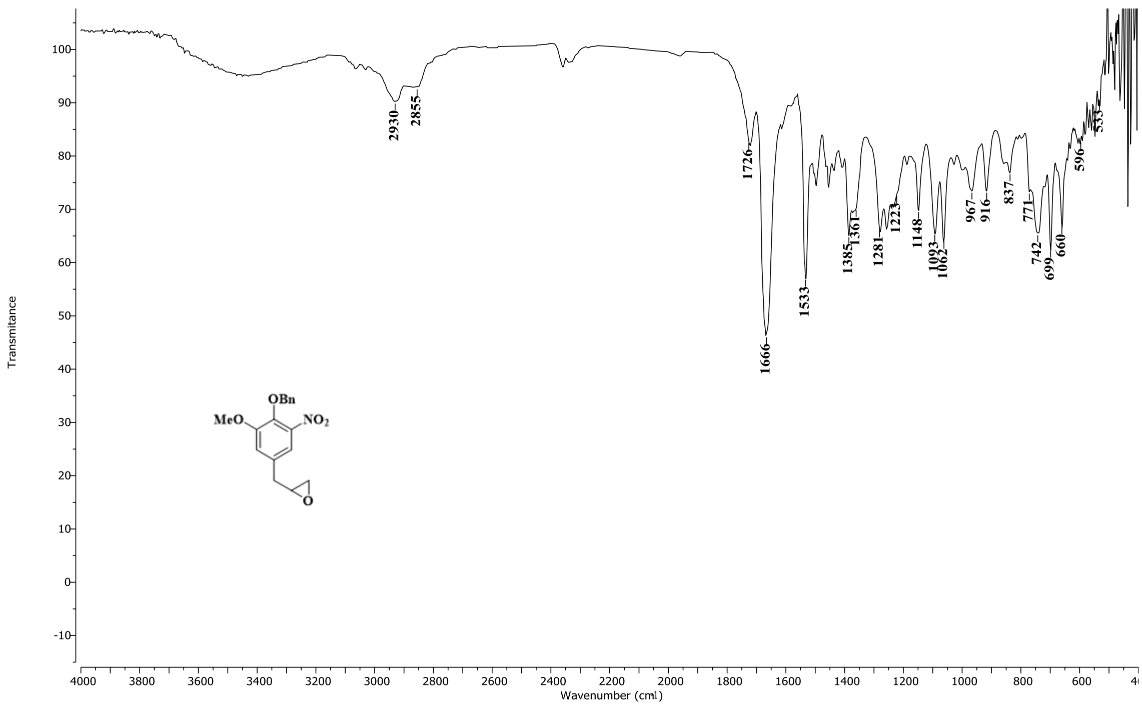

Figure 25: IR spectrum of compound **12**

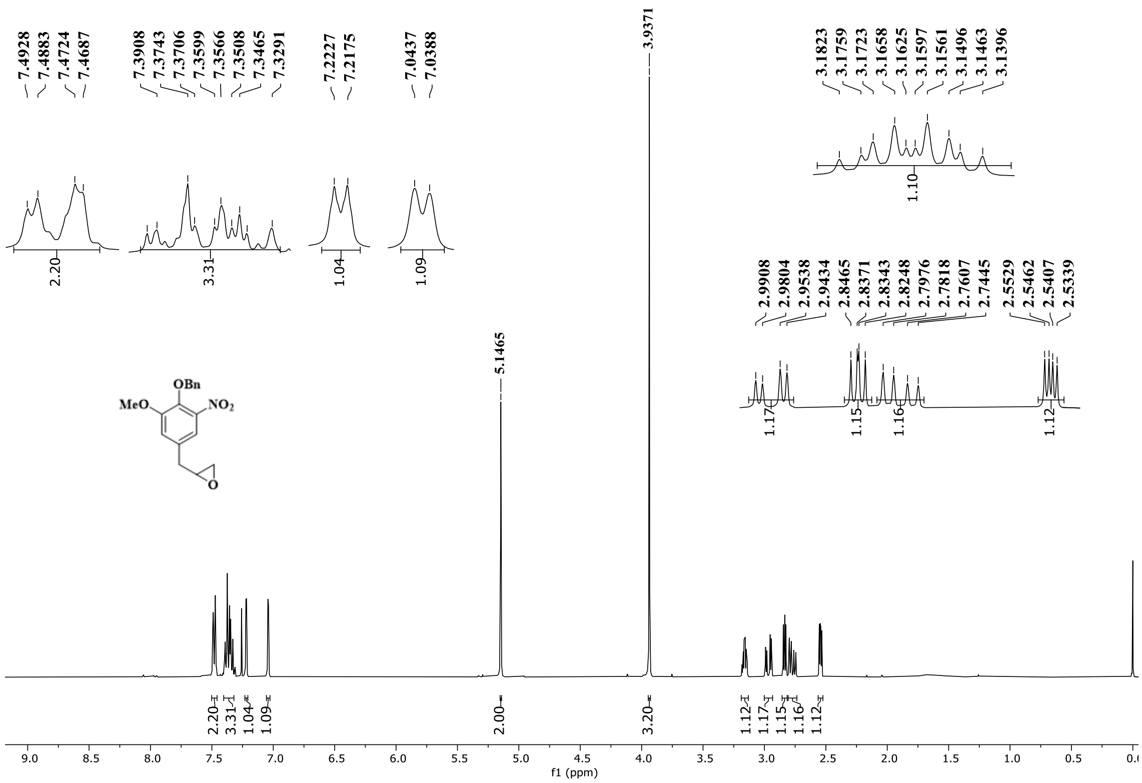

Figure 26: ^{1}H NMR spectrum of compound **12** ($CDCl_3$, 300Hz)

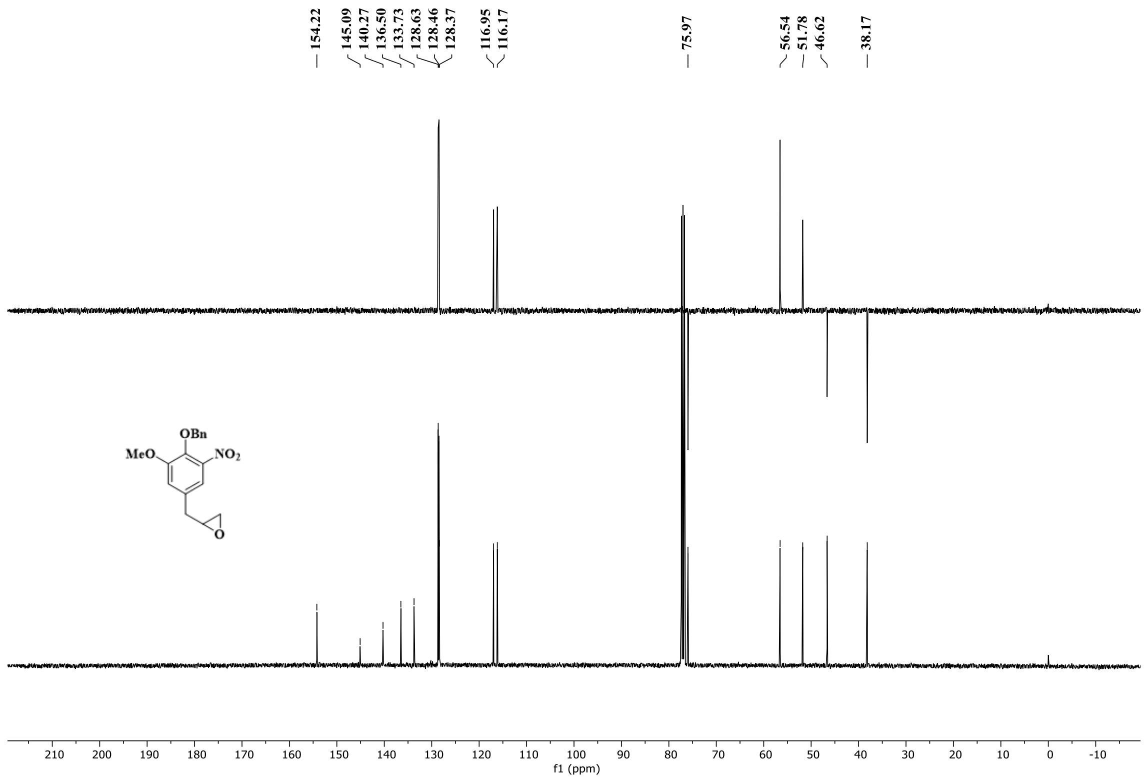

Figure 27: ^{13}C NMR spectrum and DEPT-135 of compound **12** ($CDCl_3$, 75Hz)

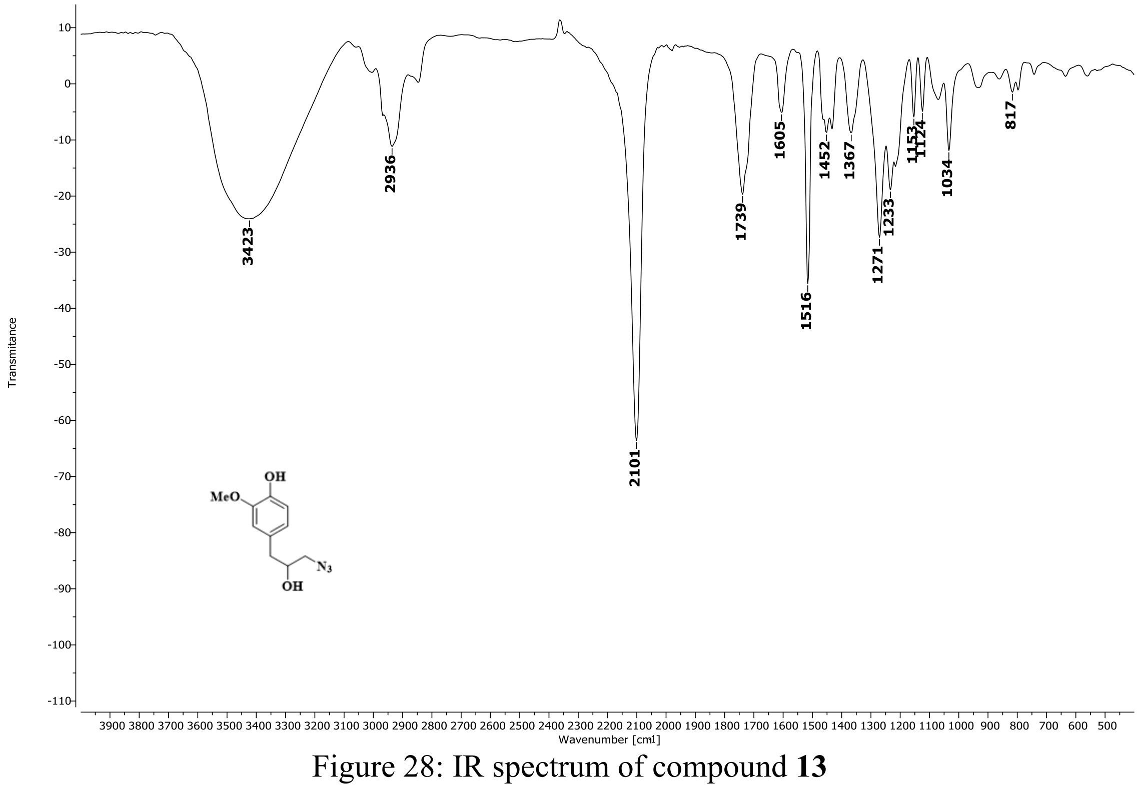

Figure 28: IR spectrum of compound **13**

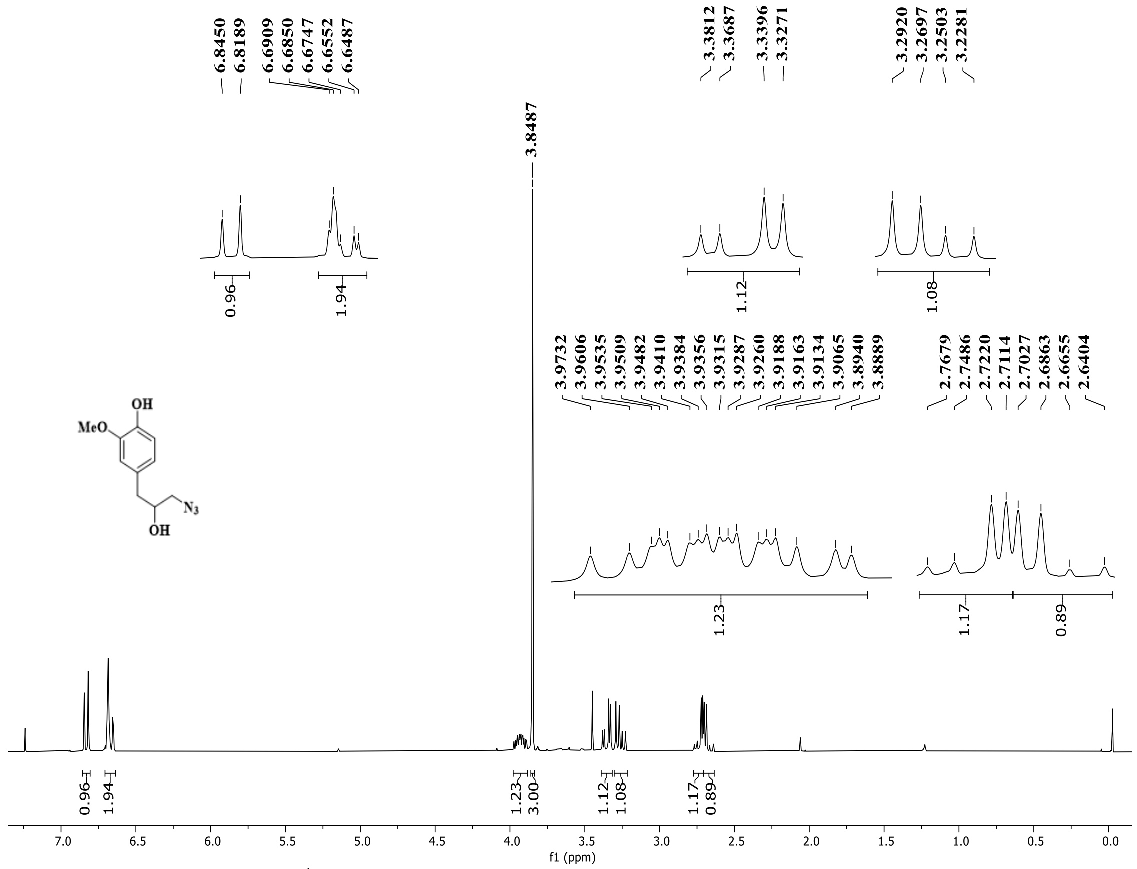

Figure 29: ^{1}H NMR spectrum of compound **13** ($CDCl_3$, 300Hz)

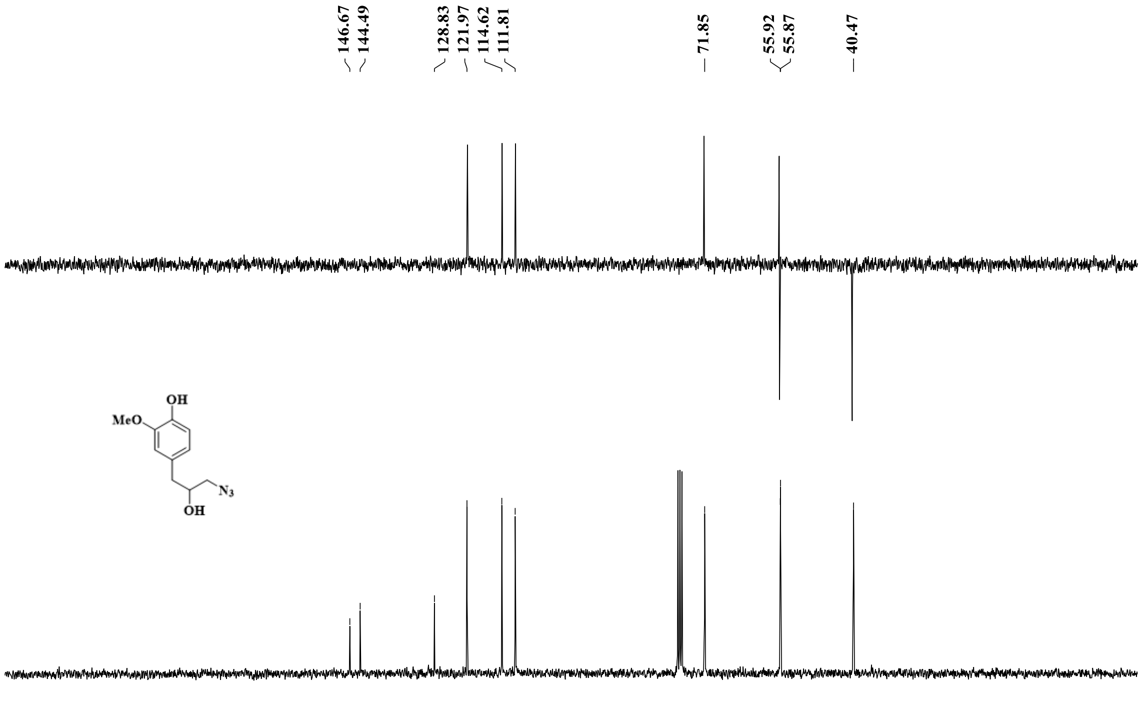

Figure 30: ^{13}C NMR spectrum and DEPT-135 of compound **13** ($CDCl_3$, 75Hz)

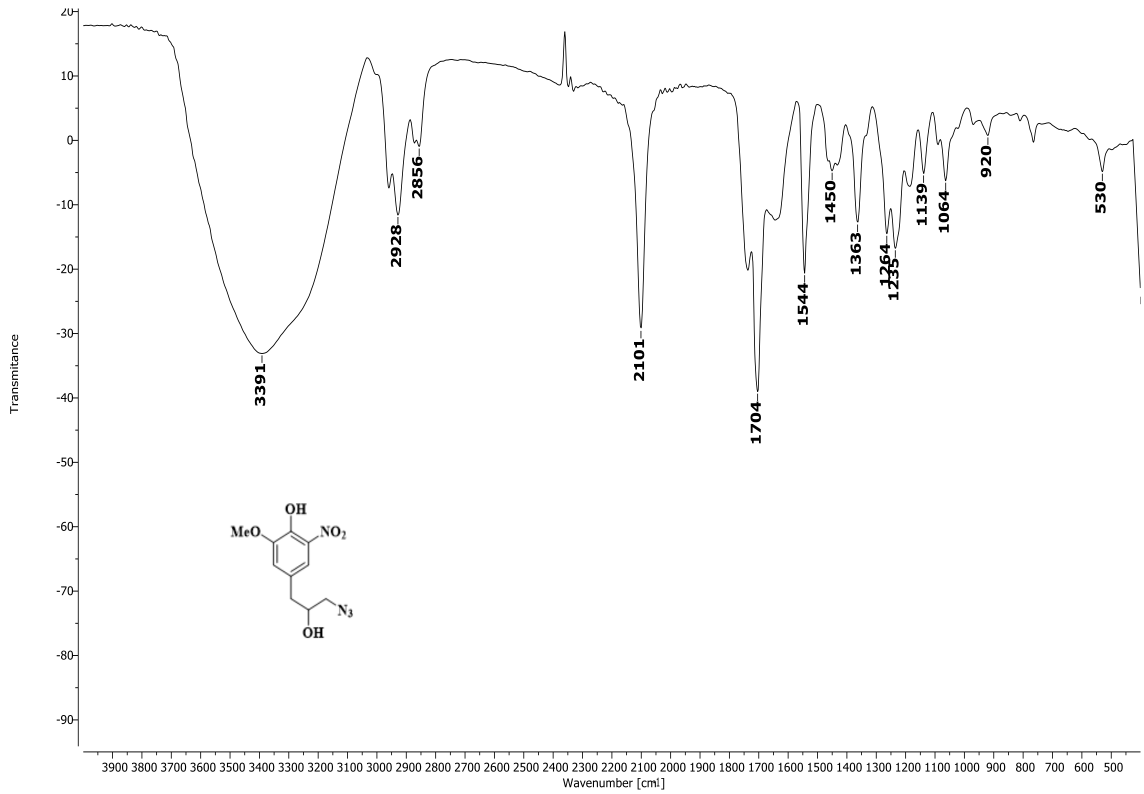

Figure 31: IR spectrum of compound **14**

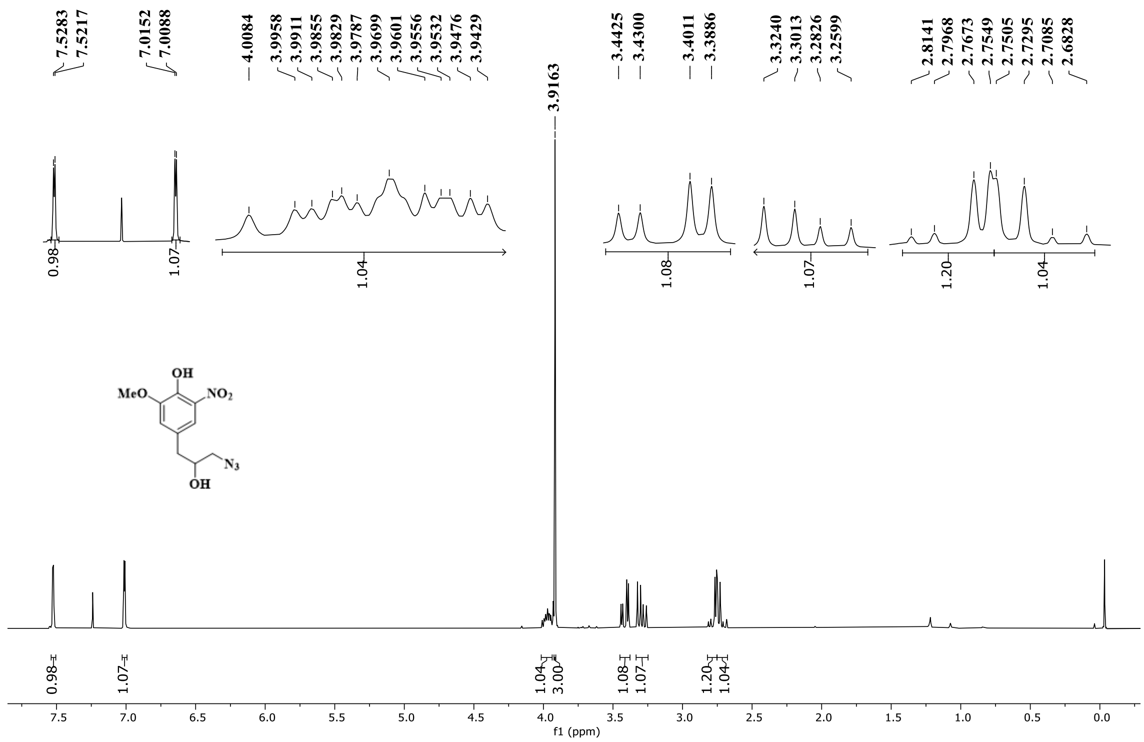

Figure 32: ^{1}H NMR spectrum of compound **14** ($CDCl_3$, 300Hz)

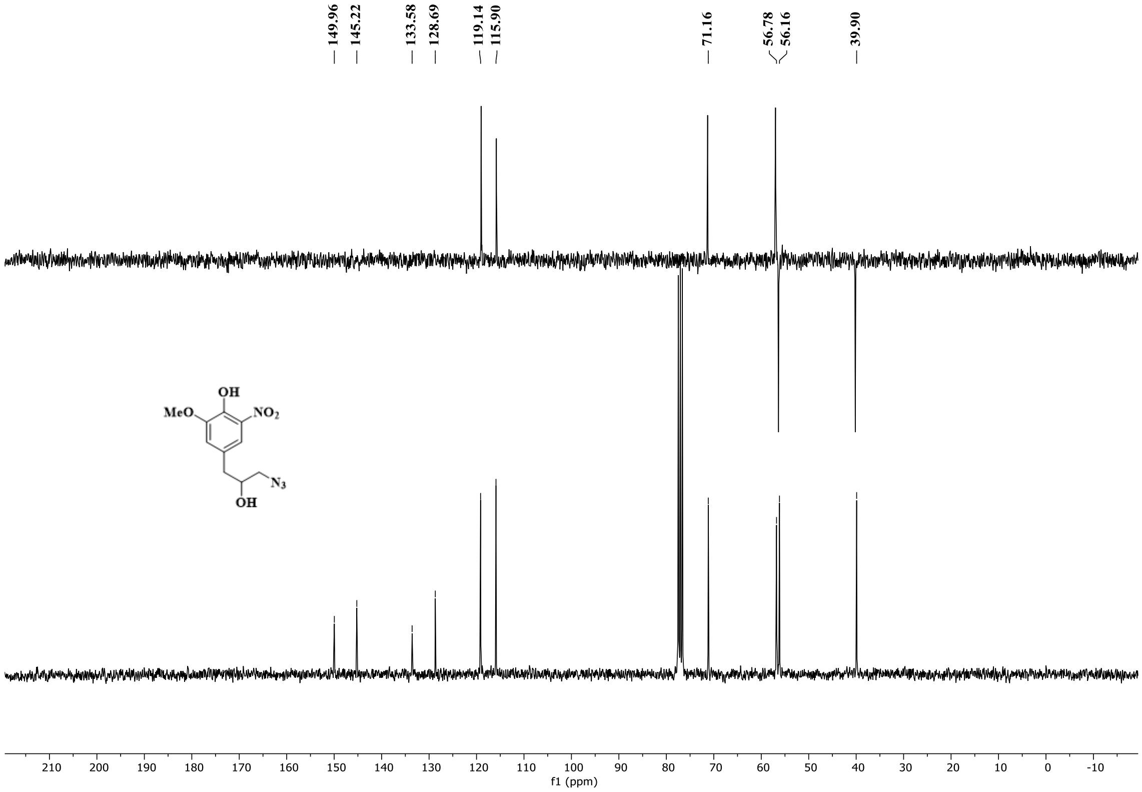

Figure 33: ^{13}C NMR spectrum and DEPT-135 of compound **14** ($CDCl_3$, 75Hz)

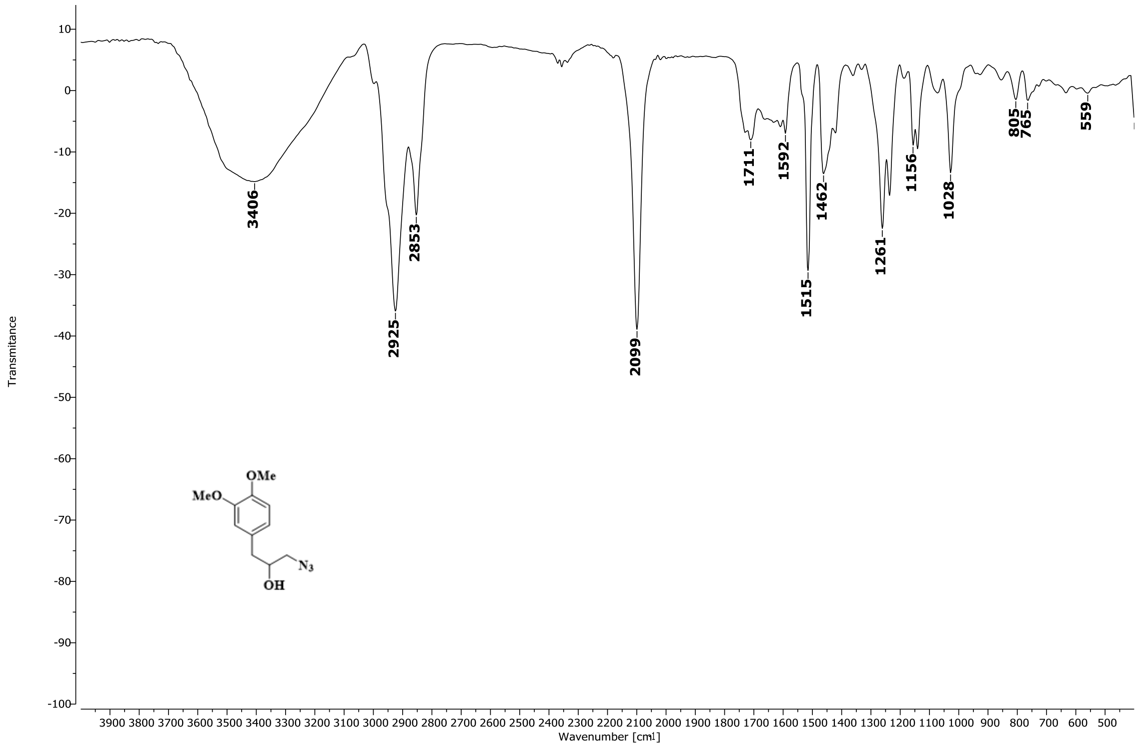

Figure 34: IR spectrum of compound **15**

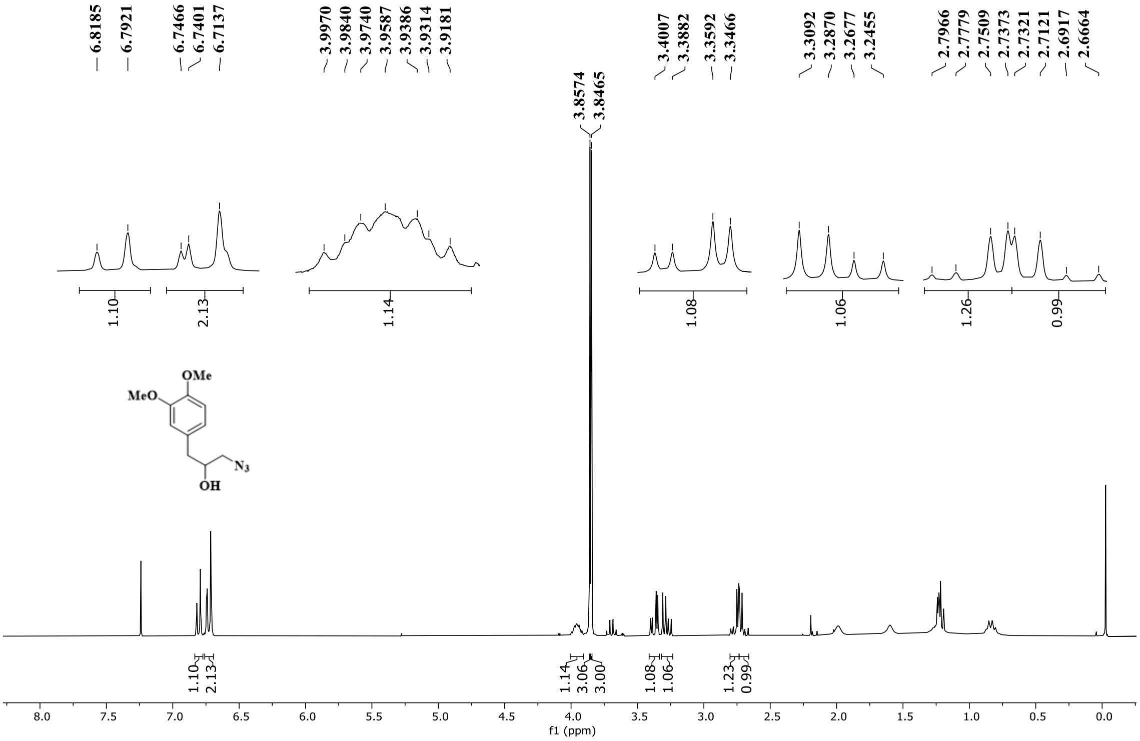

Figure 35: ^{1}H NMR spectrum of compound **15** ($CDCl_3$, 300Hz)

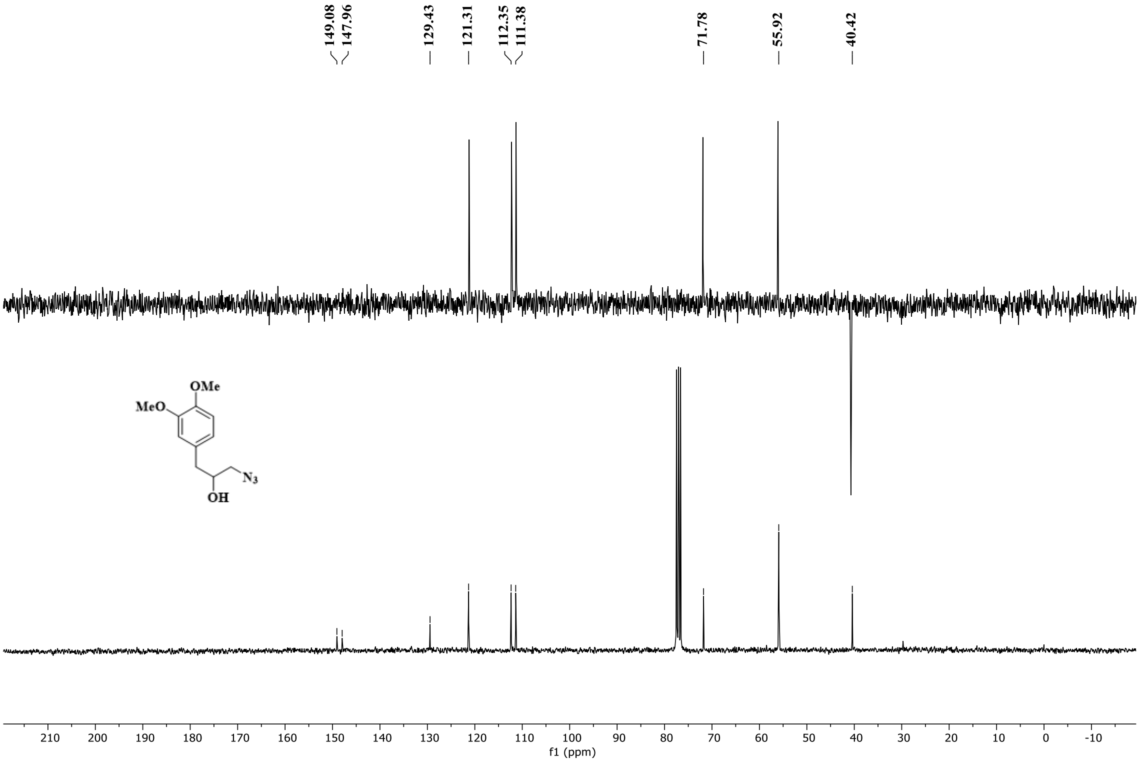

Figure 36: ^{13}C NMR spectrum and DEPT-135 of compound **15** ($CDCl_3$, 75Hz)

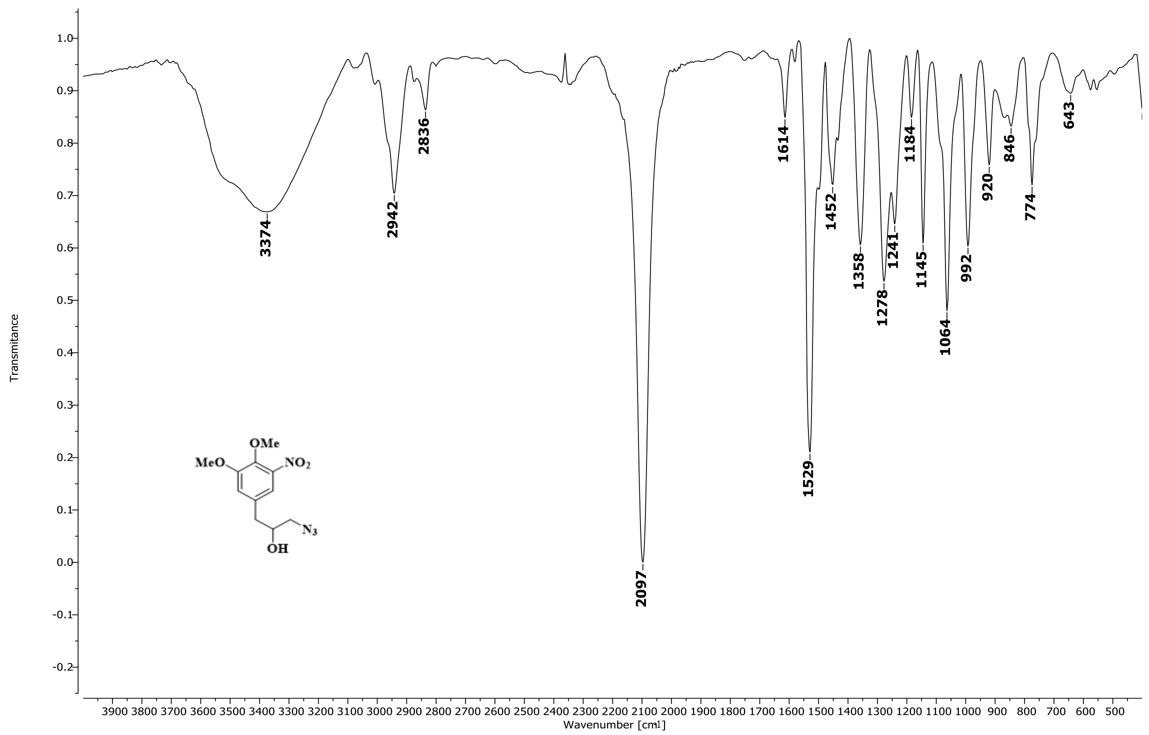

Figure 37: IR spectrum of compound **16**

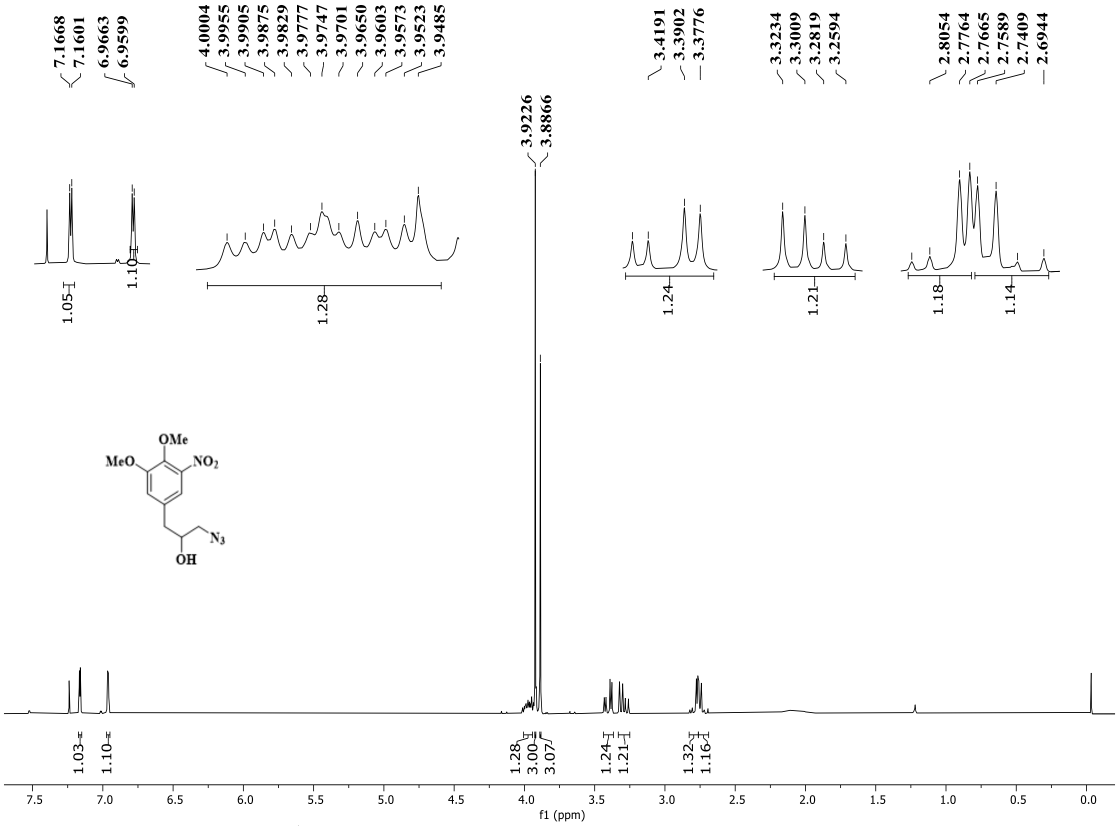

Figure 38: ^{1}H NMR spectrum of compound **16** ($CDCl_3$, 300Hz)

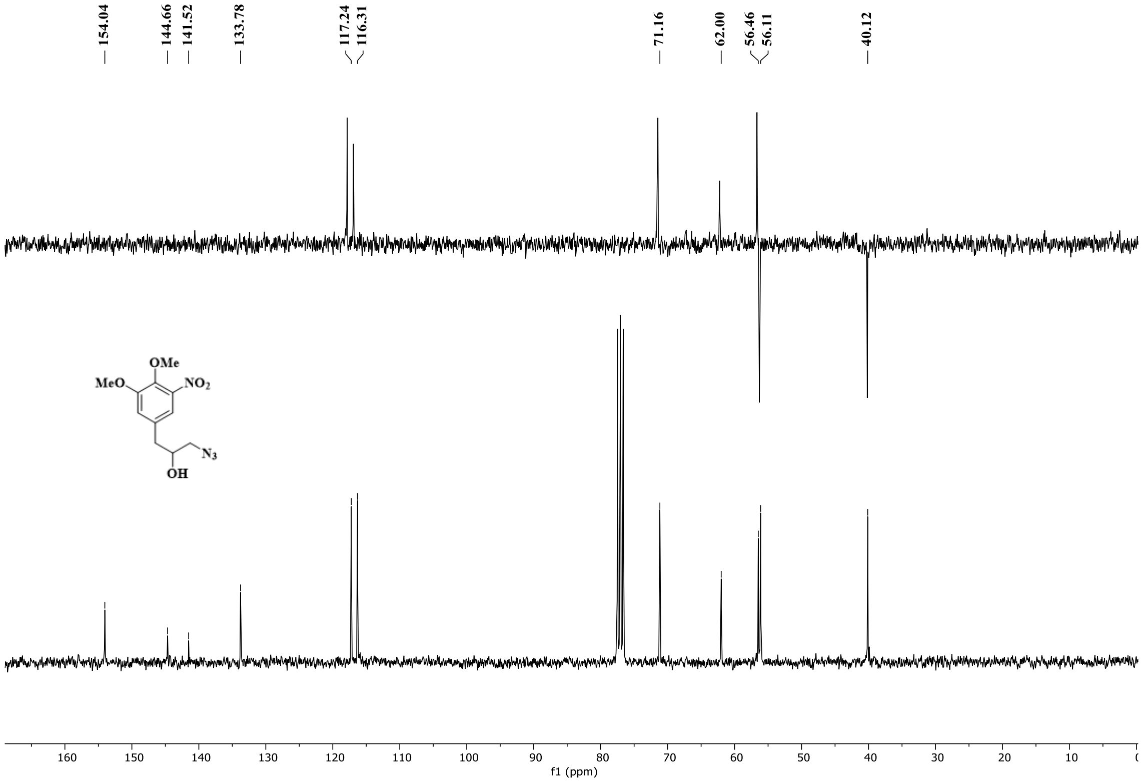

Figure 39: ^{13}C NMR spectrum and DEPT-135 of compound **16** ($CDCl_3$, 75Hz)

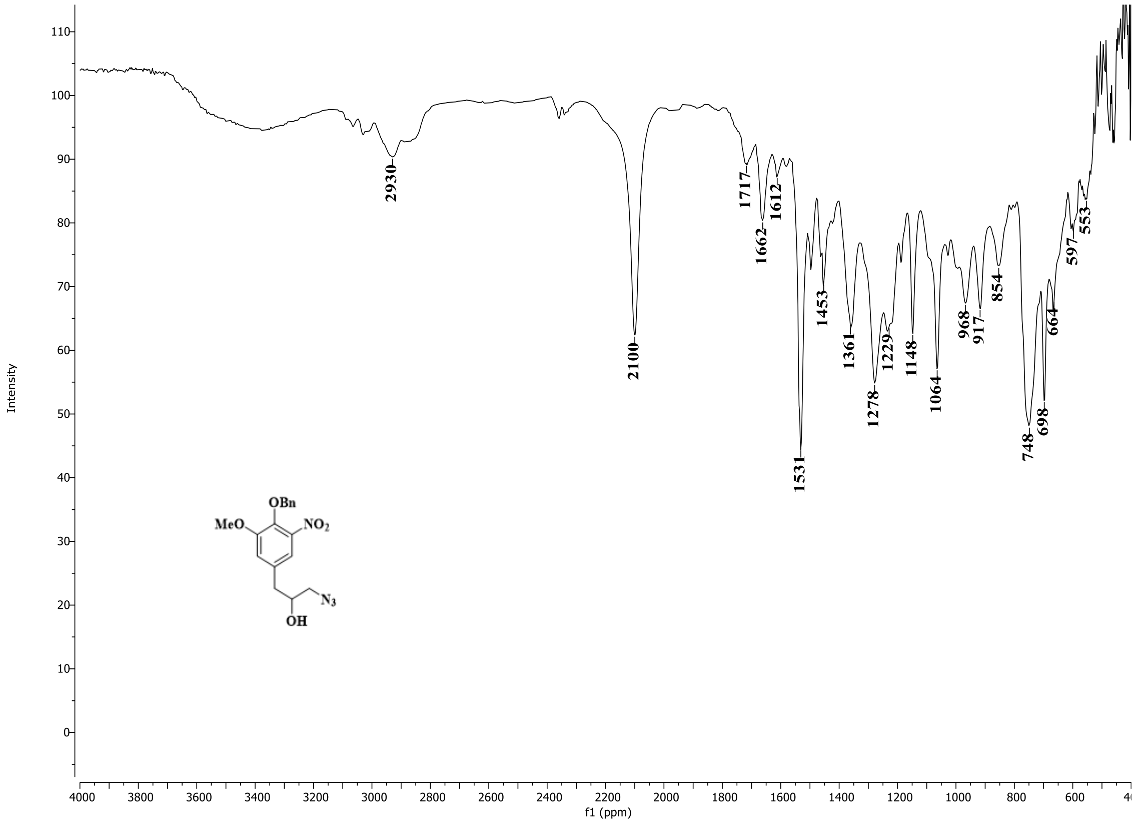

Figure 40: IR spectrum of compound **17**

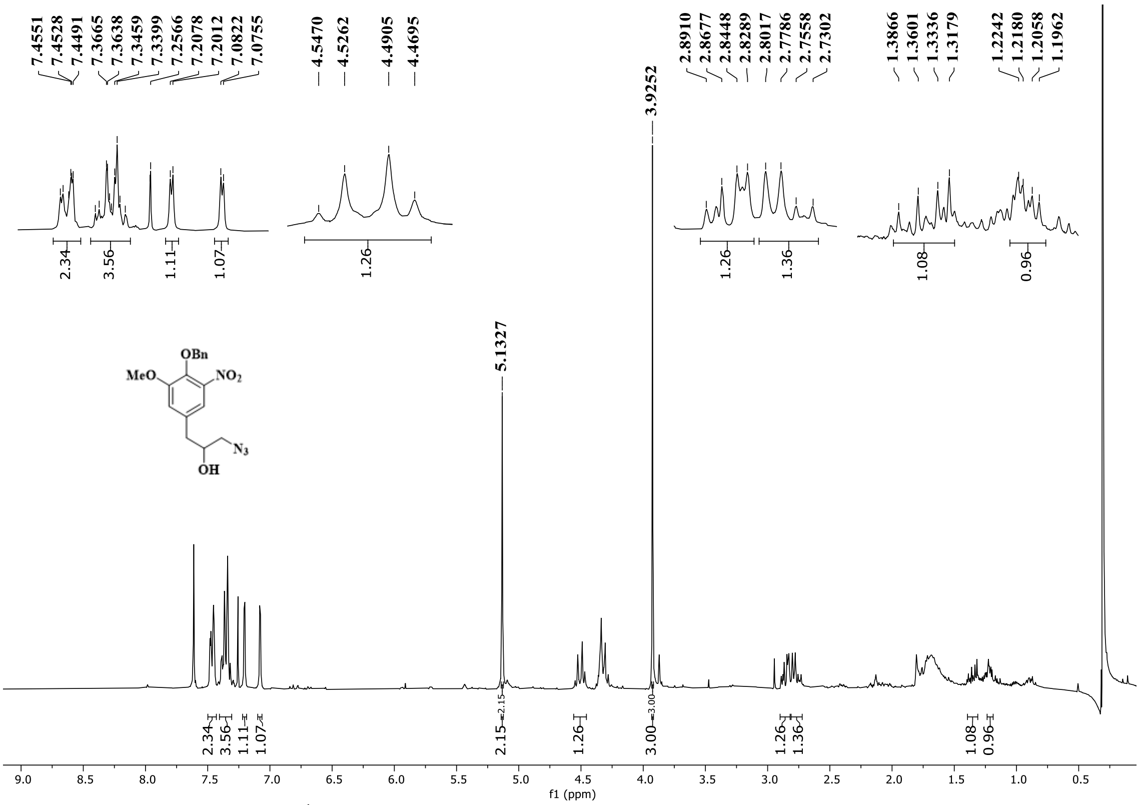

Figure 41: ^{1}H NMR spectrum of compound **17** ($CDCl_3$, 300Hz)

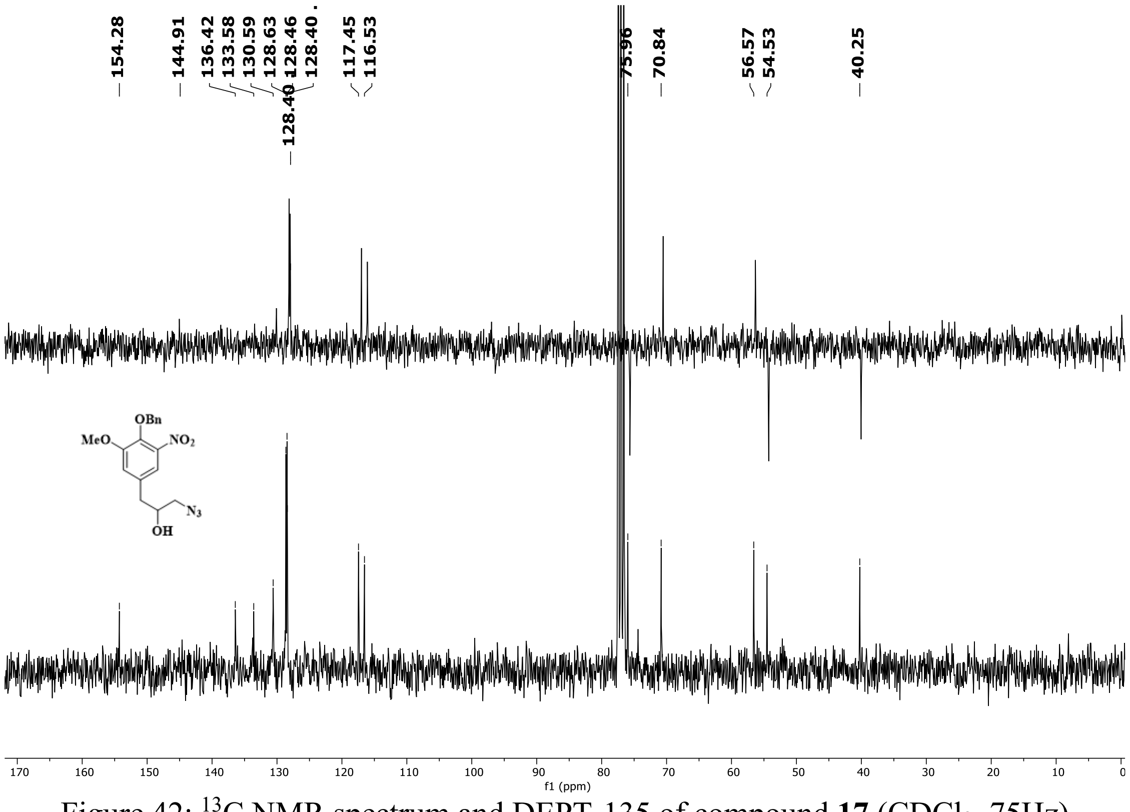

Figure 42: ^{13}C NMR spectrum and DEPT-135 of compound **17** ($CDCl_3$, 75Hz)

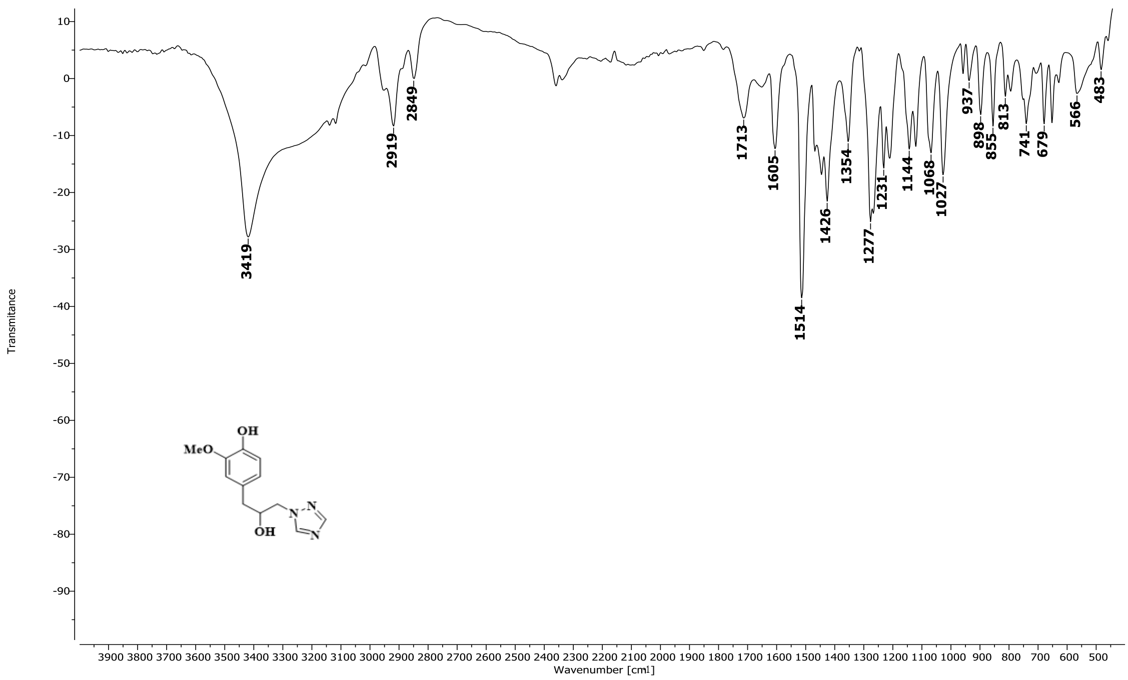

Figure 43: IR spectrum of compound **18**

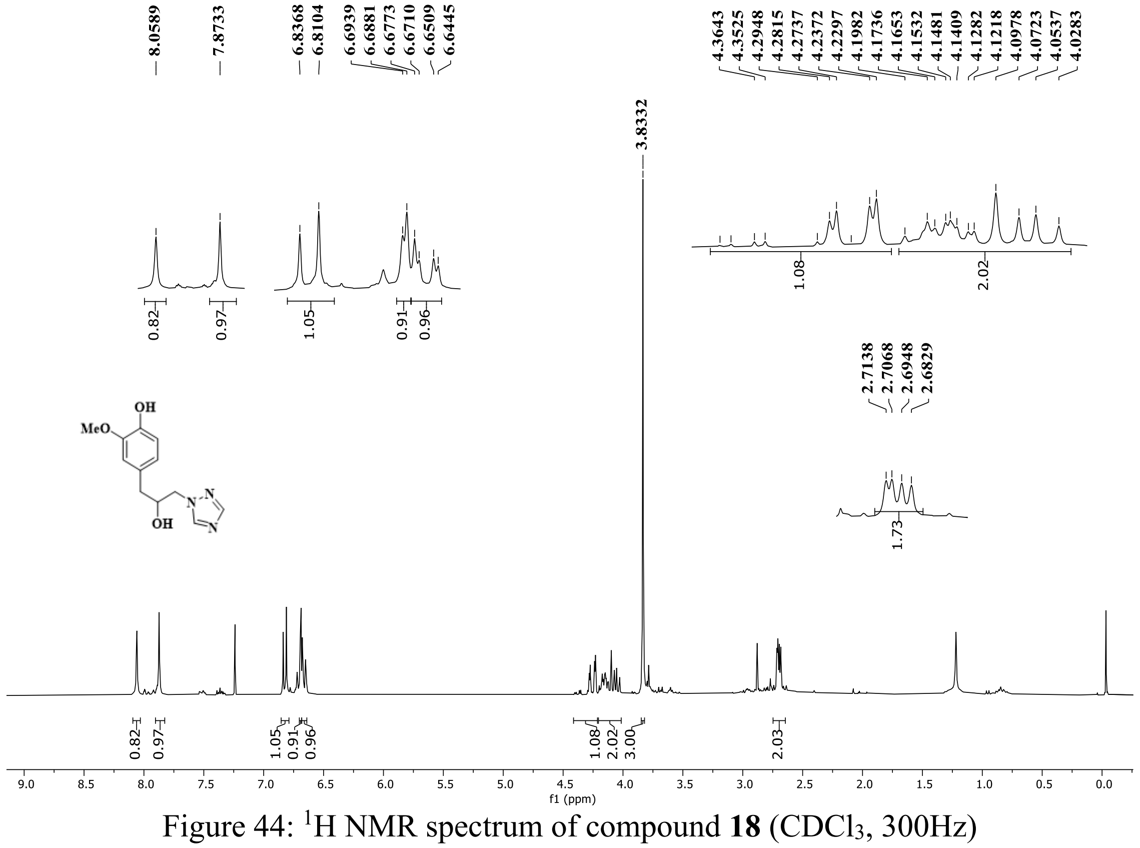

Figure 44: ^{1}H NMR spectrum of compound **18** ($CDCl_3$, 300Hz)

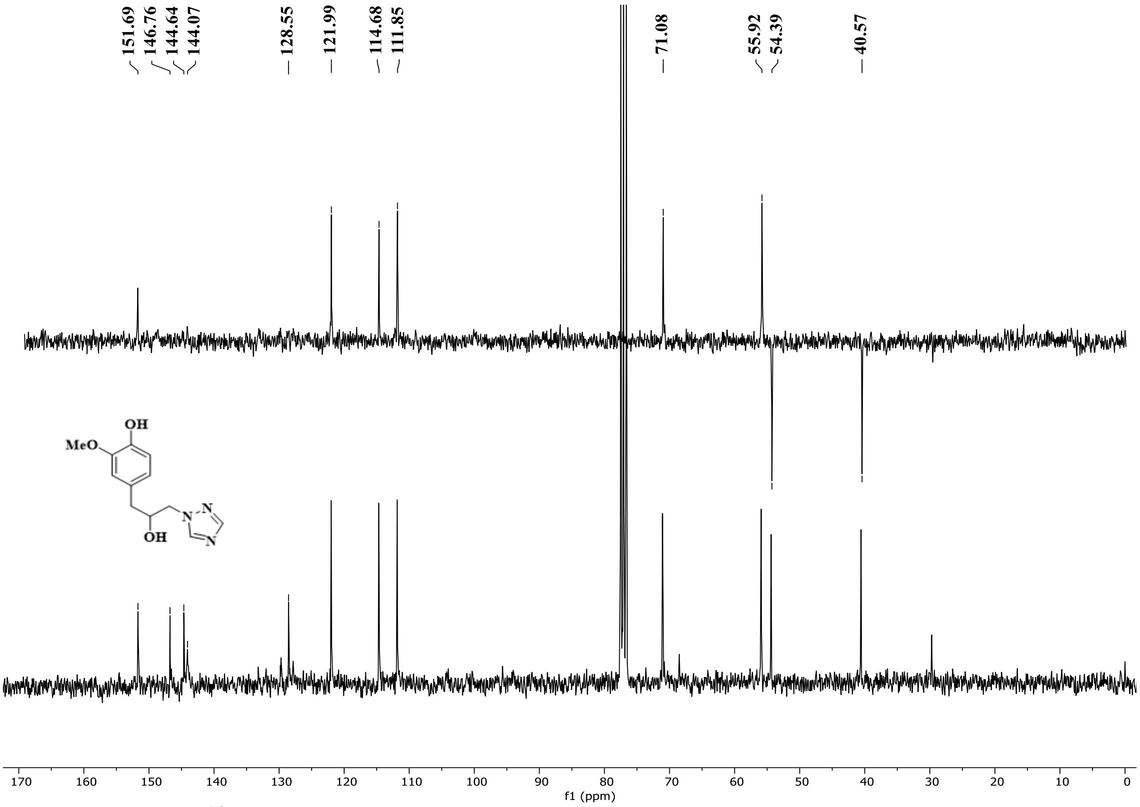

Figure 45: ^{13}C NMR spectrum and DEPT-135 of compound **18** ($CDCl_3$, 75Hz)

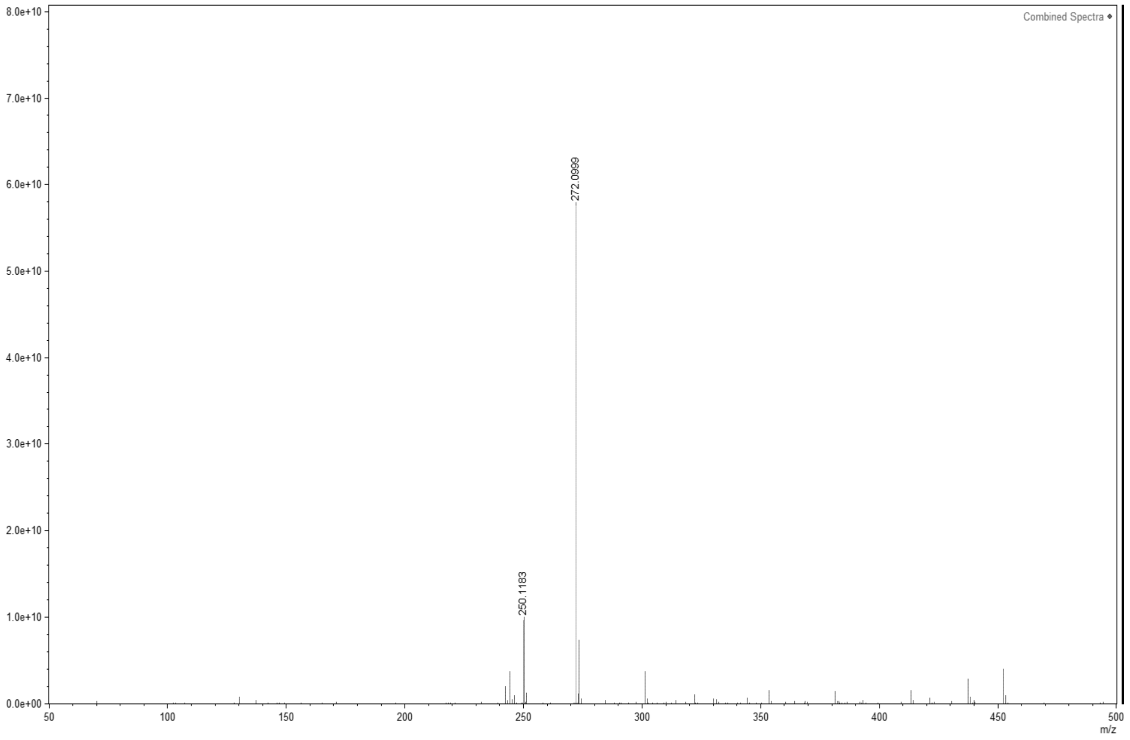

Figure 46: Mass spectrum of compound **18**

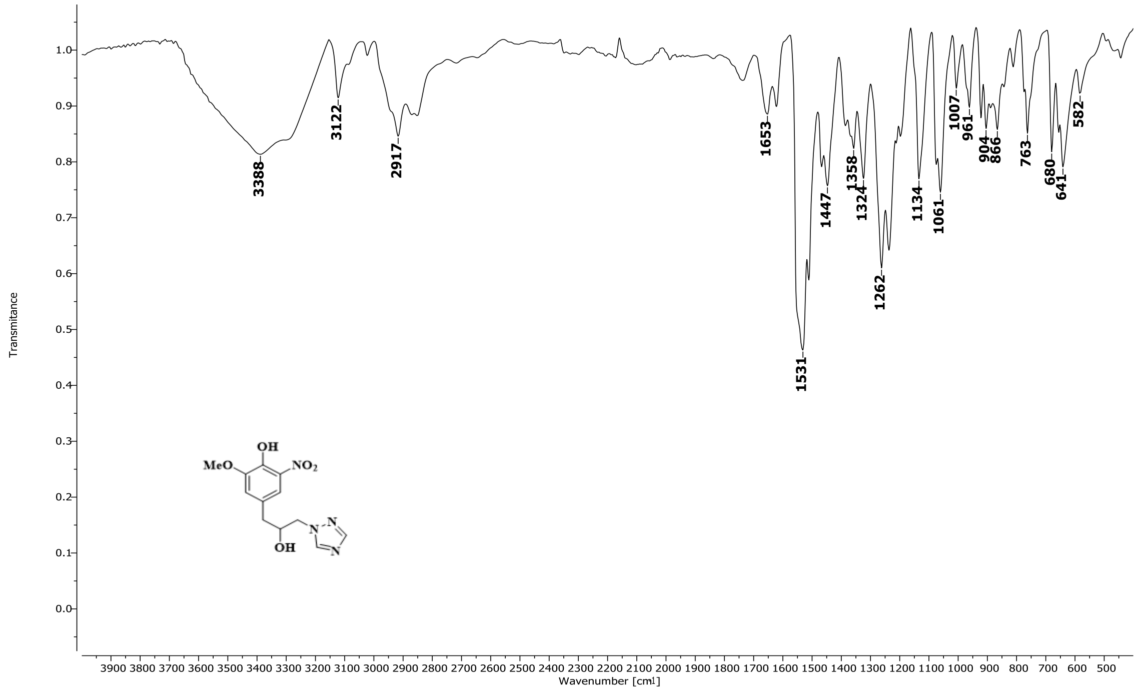

Figure 47: IR spectrum of compound **19**

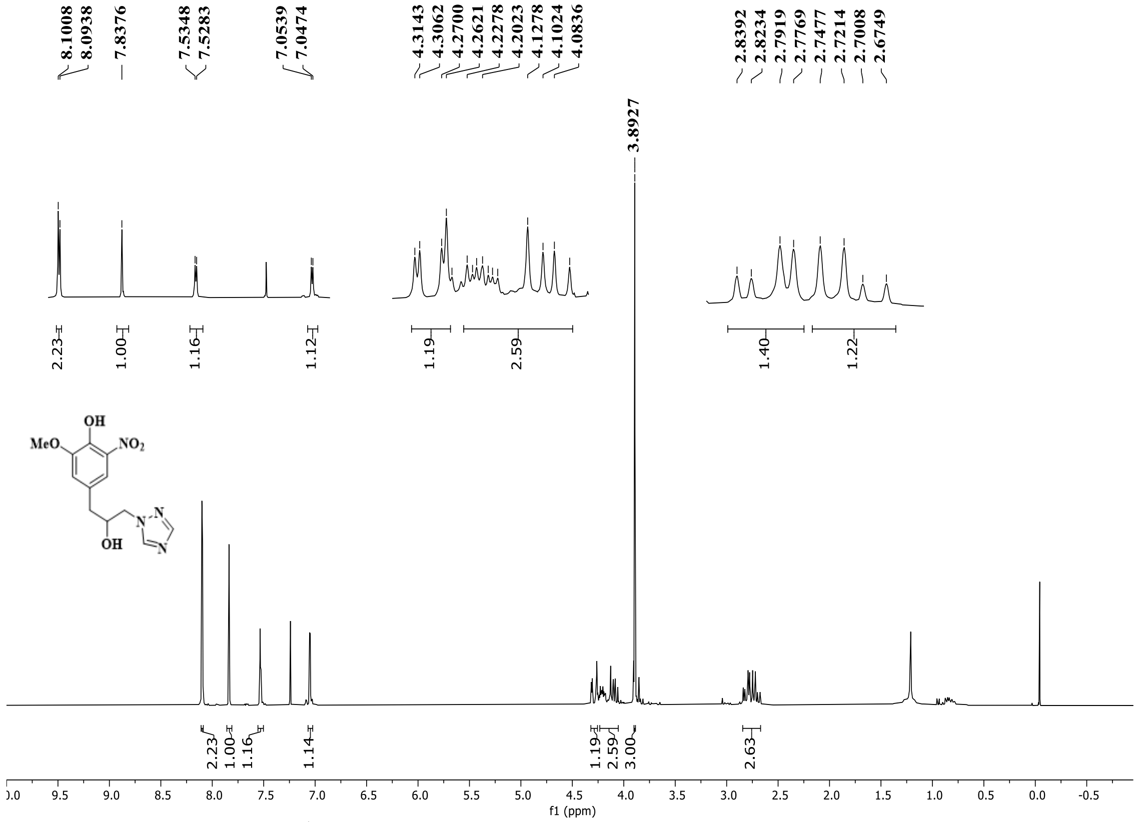

Figure 48: ^{1}H NMR spectrum of compound **19** ($CDCl_3$, 300Hz)

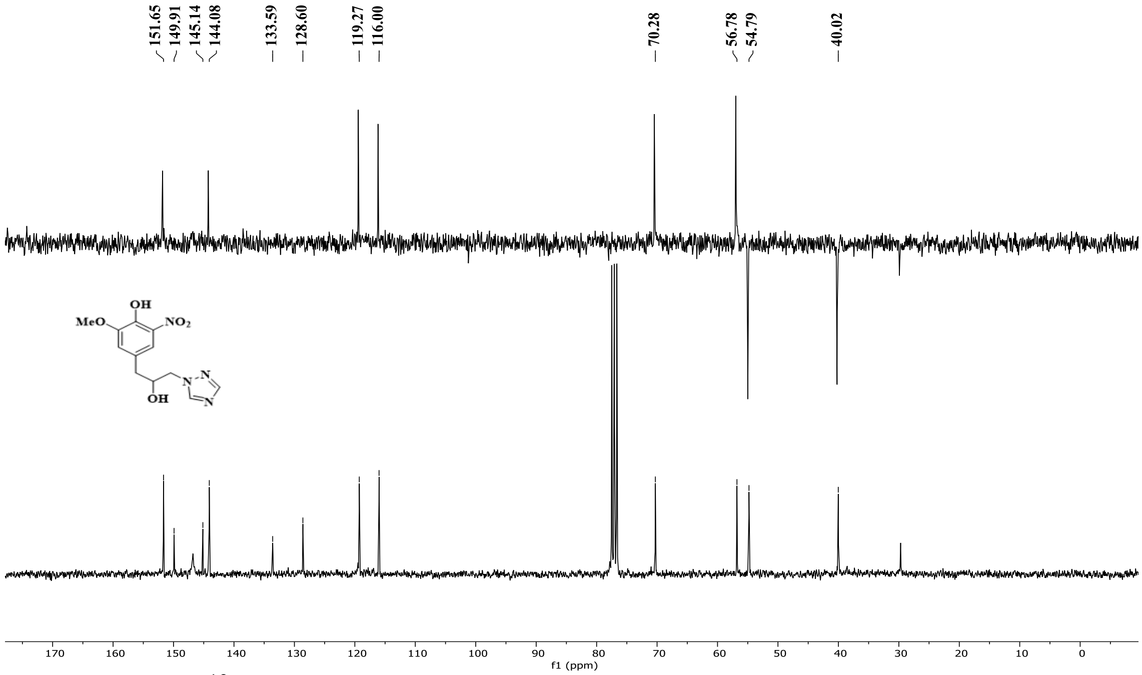

Figure 49: ^{13}C NMR spectrum and DEPT-135 of compound **19** ($CDCl_3$, 75Hz)

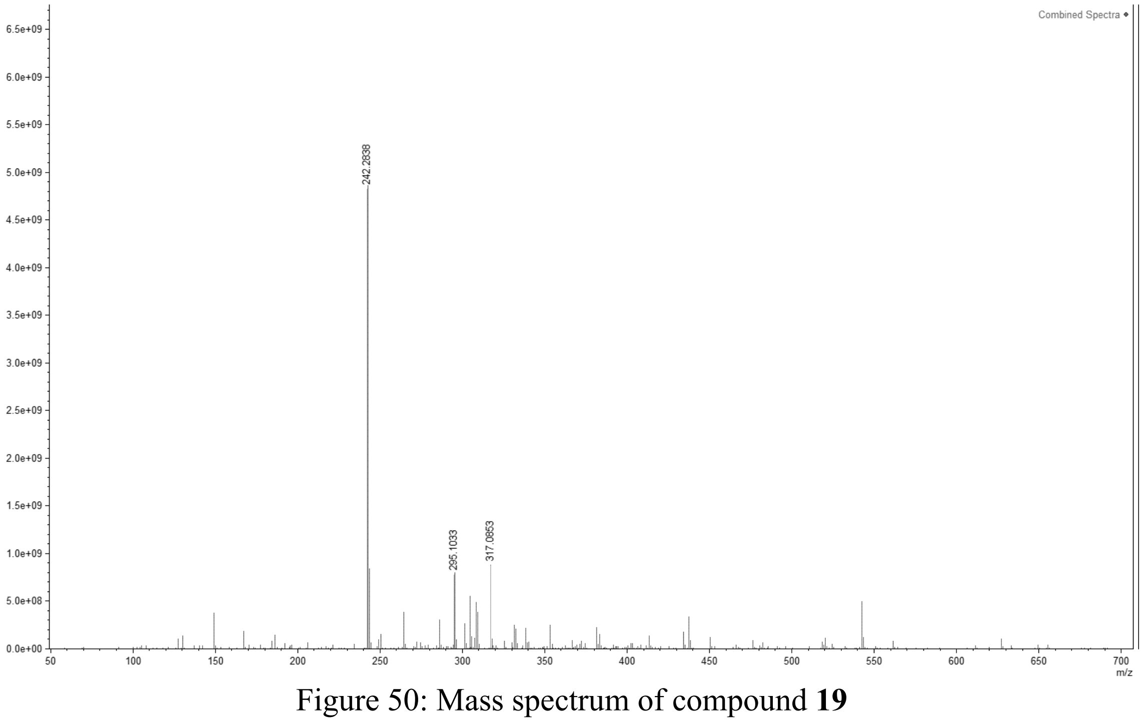

Figure 50: Mass spectrum of compound **19**

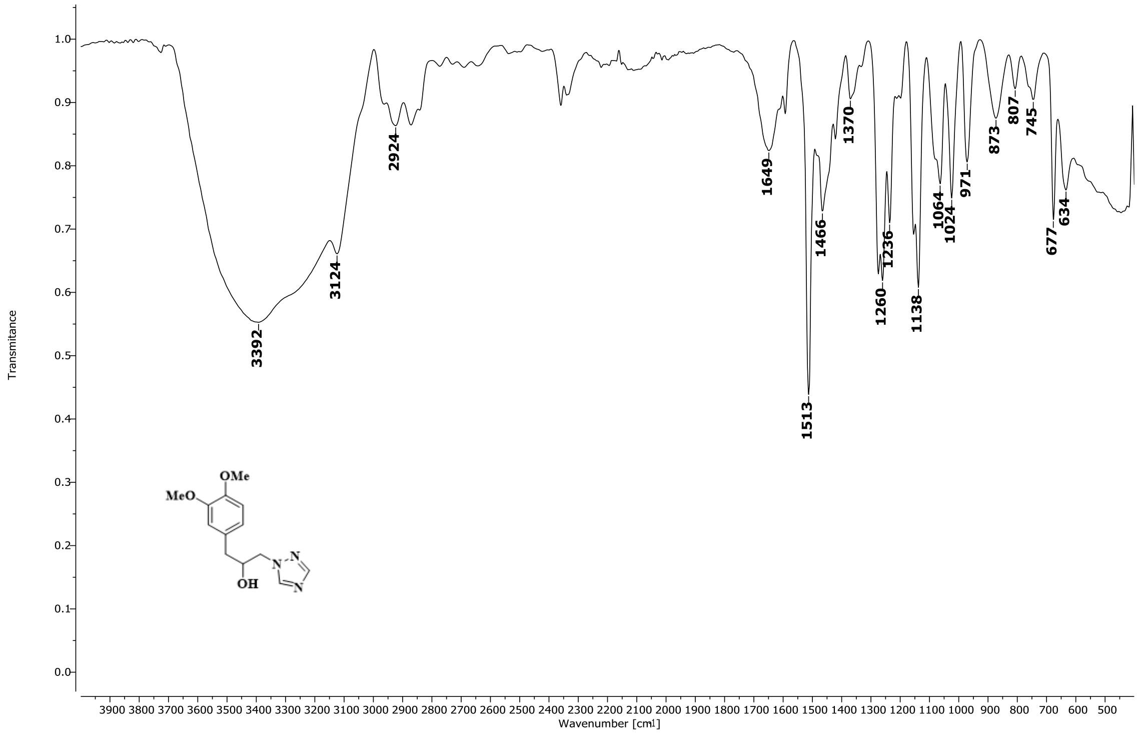

Figure 51: IR spectrum of compound **20**

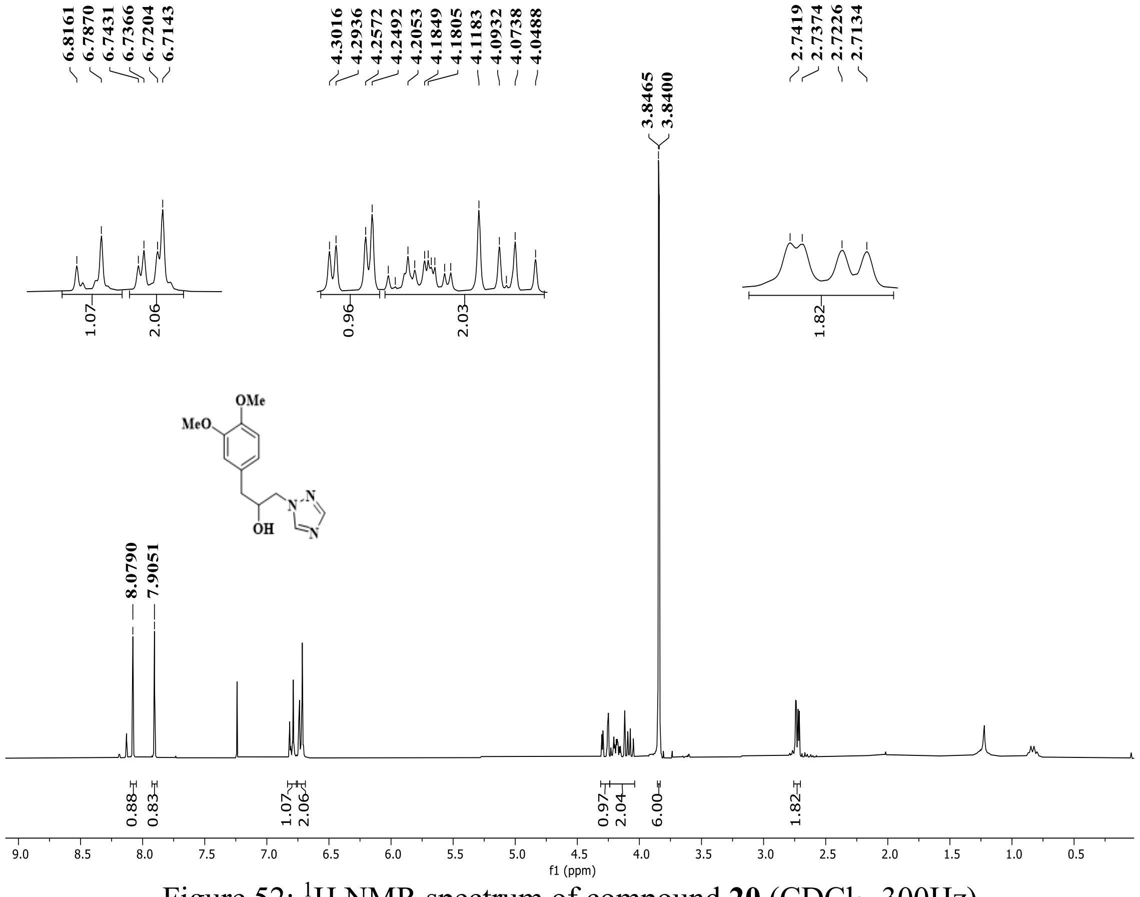

Figure 52: ^{1}H NMR spectrum of compound **20** ($CDCl_3$, 300Hz)

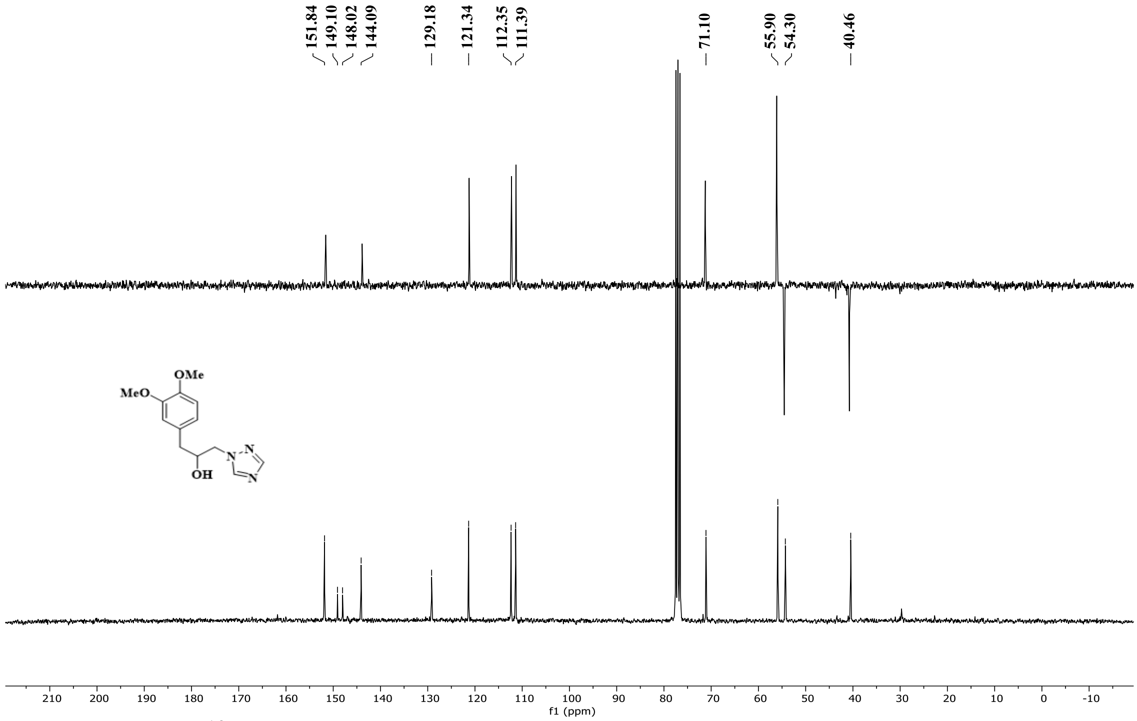

Figure 53: ^{13}C NMR spectrum and DEPT-135 of compound **20** ($CDCl_3$, 75Hz)

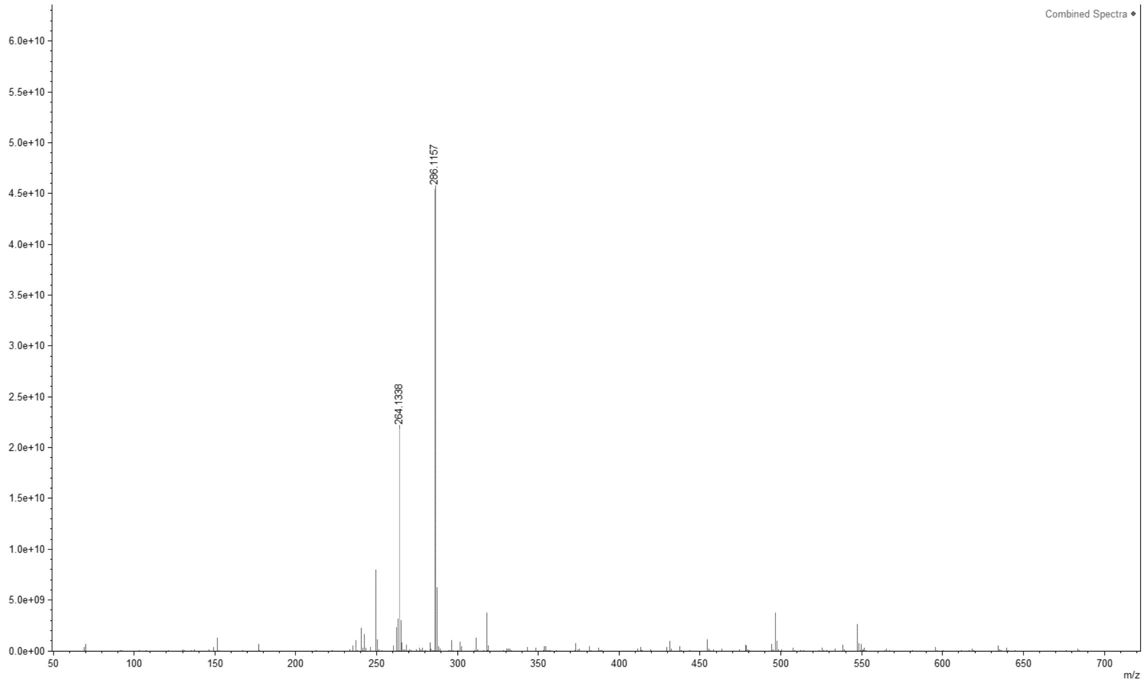

Figure 54: Mass spectrum of compound **20**

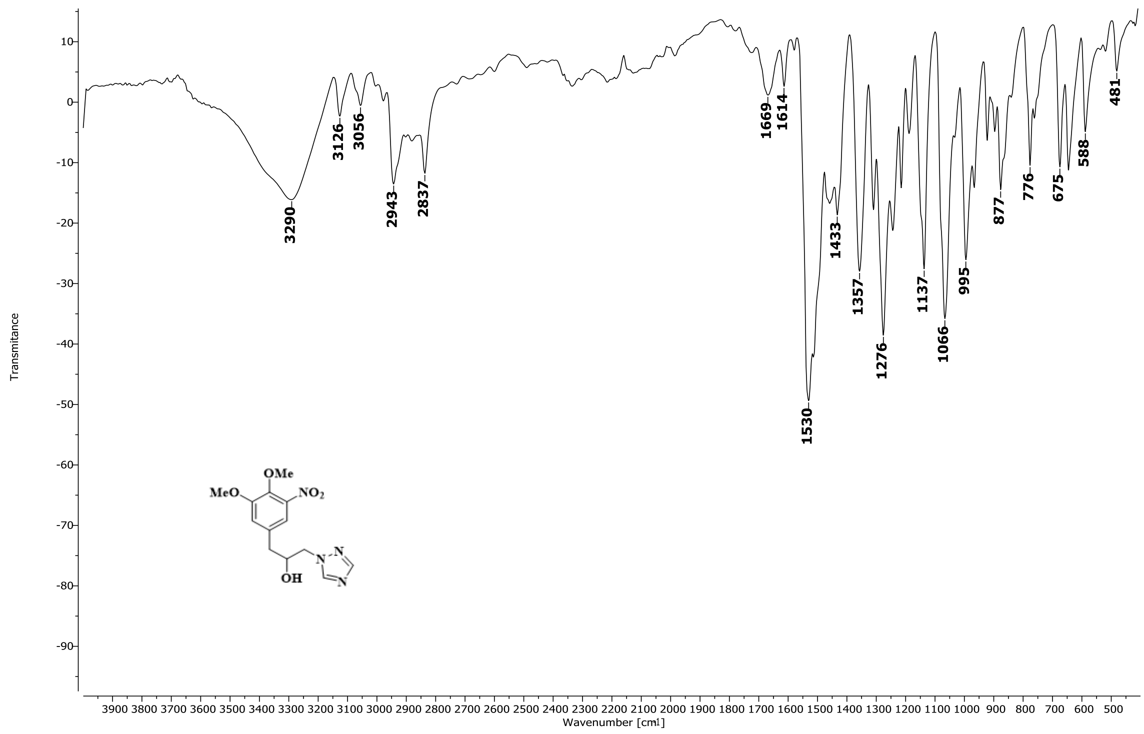

Figure 55: IR spectrum of compound **21**

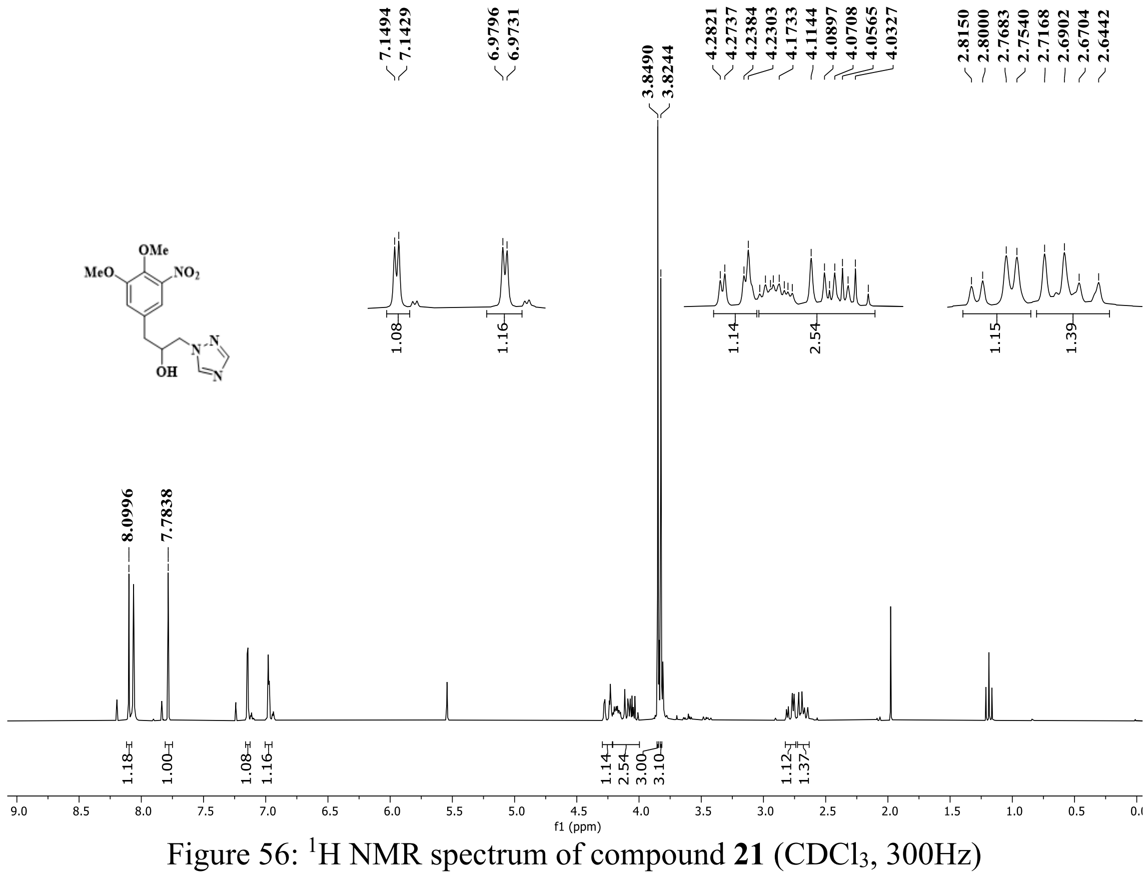

Figure 56: ^{1}H NMR spectrum of compound **21** ($CDCl_3$, 300Hz)

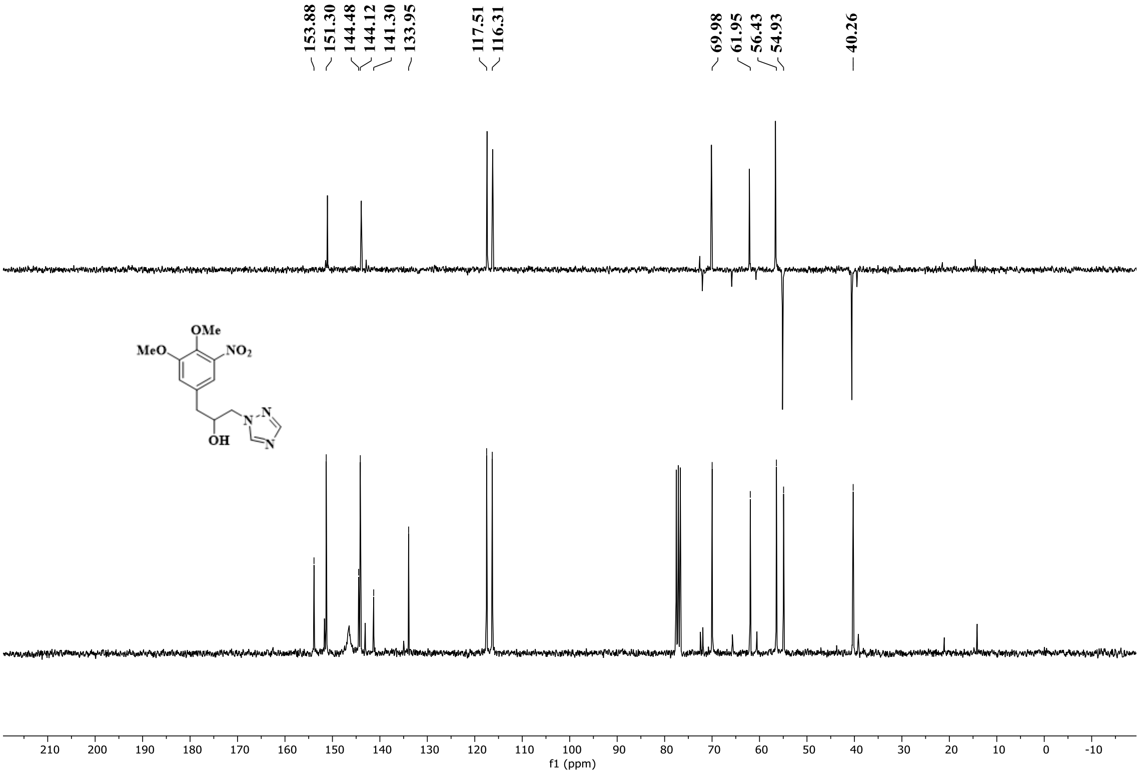

Figure 57: ^{13}C NMR spectrum and DEPT-135 of compound **21** ($CDCl_3$, 75Hz)

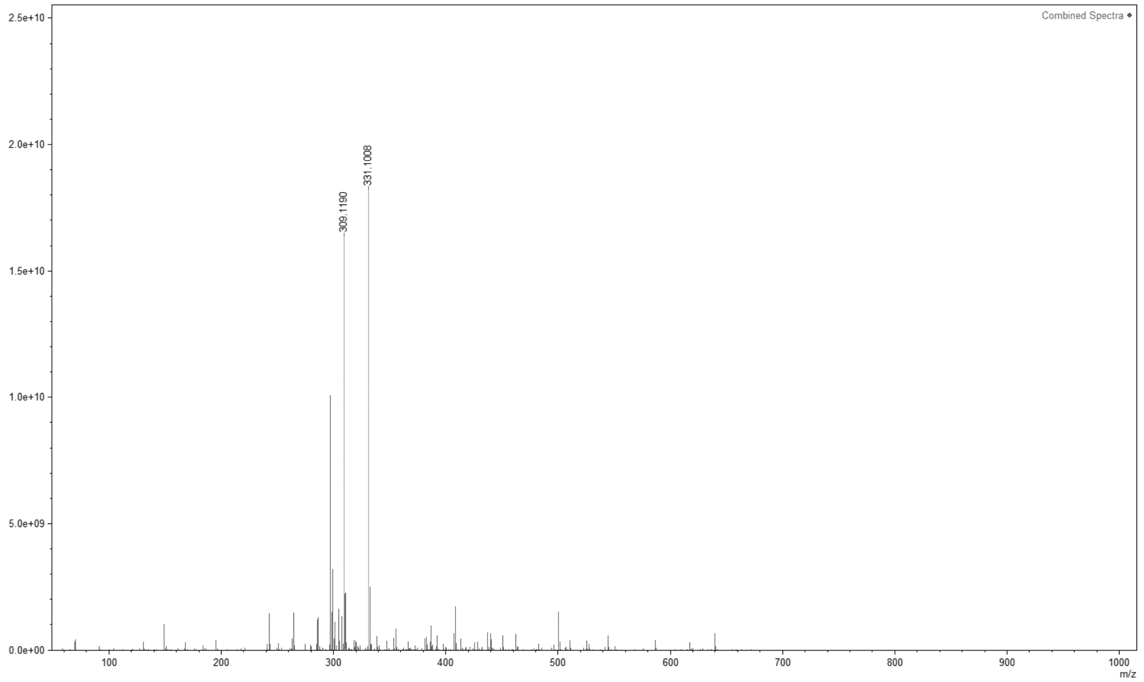

Figure 58: Mass spectrum of compound **21**

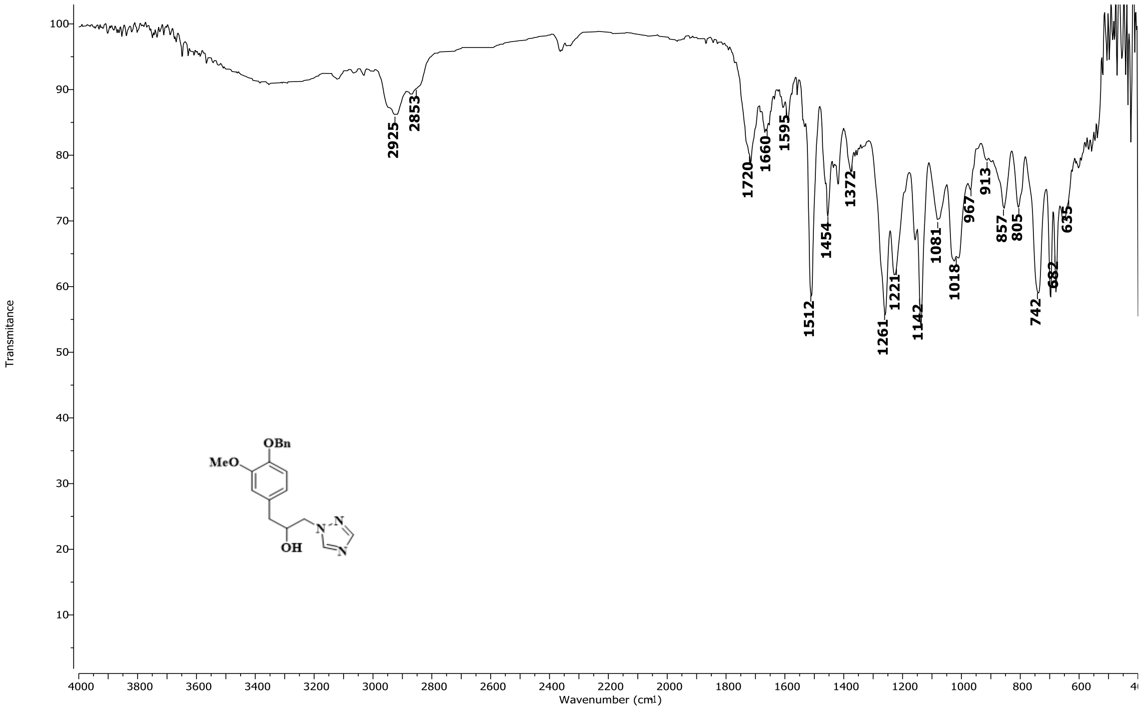

Figure 59: IR spectrum of compound **22**

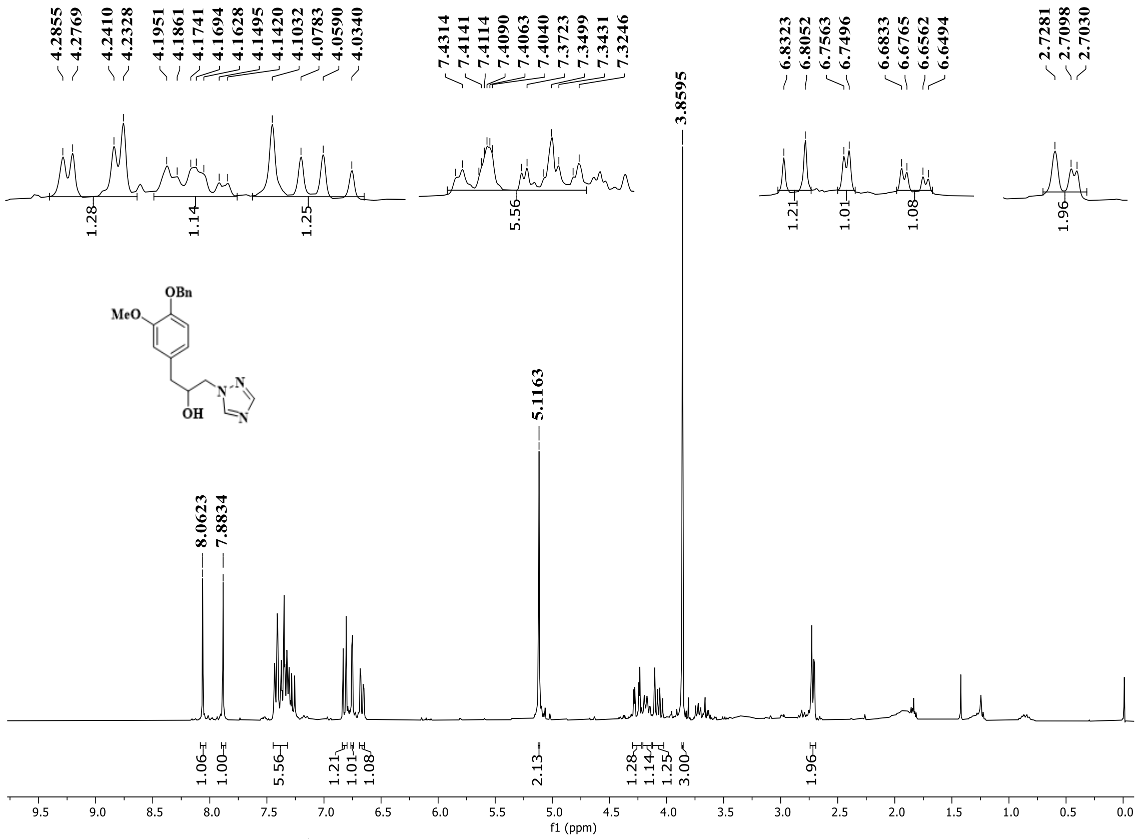

Figure 60: ^{1}H NMR spectrum of compound **22** ($CDCl_3$, 300Hz)

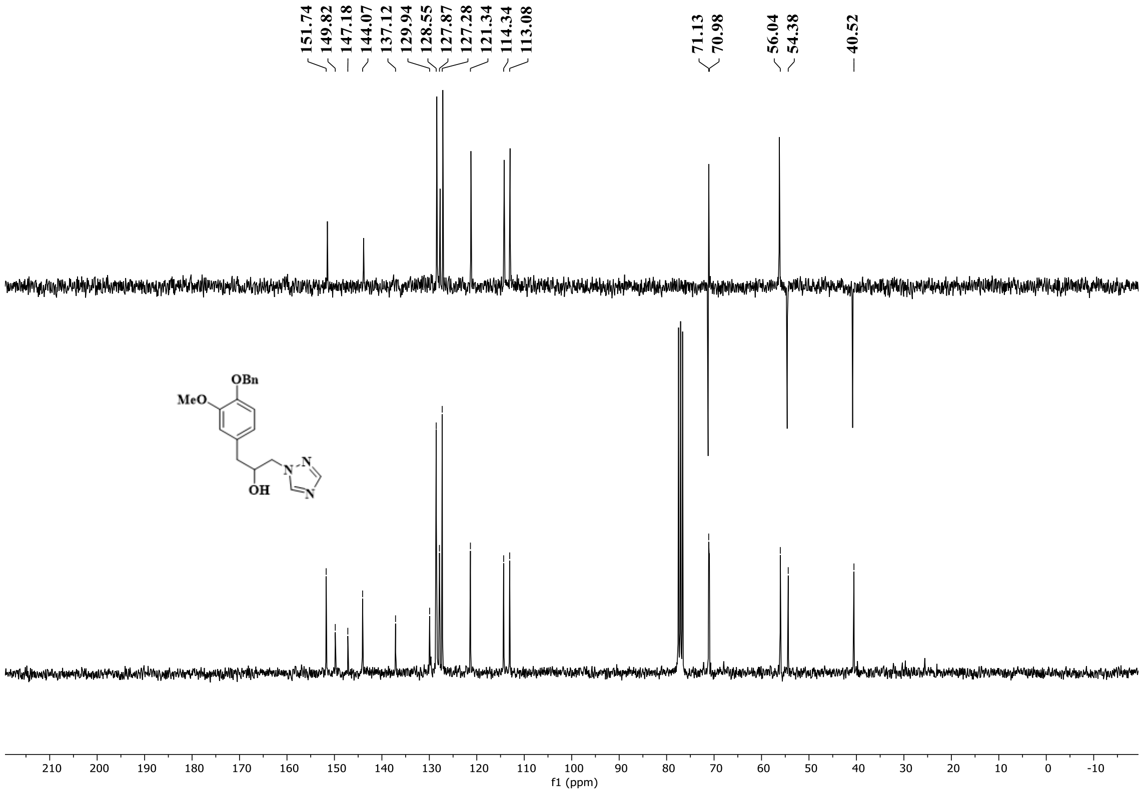

Figure 61: ^{13}C NMR spectrum and DEPT-135 of compound **22** ($CDCl_3$, 75Hz)

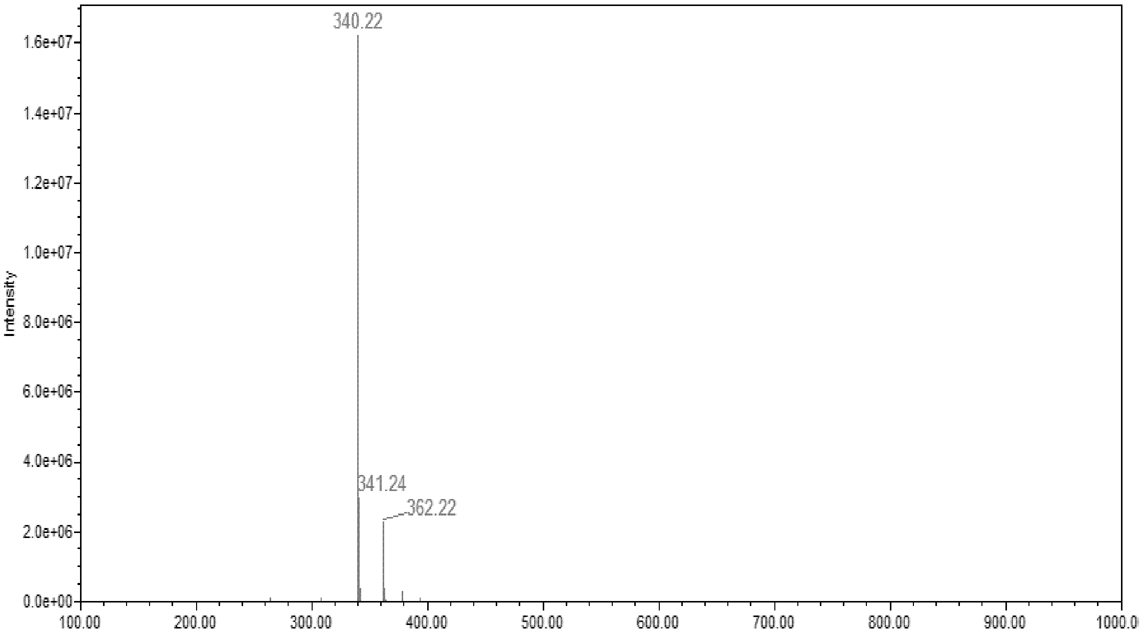

Figure 62: Mass spectrum of compound **22**

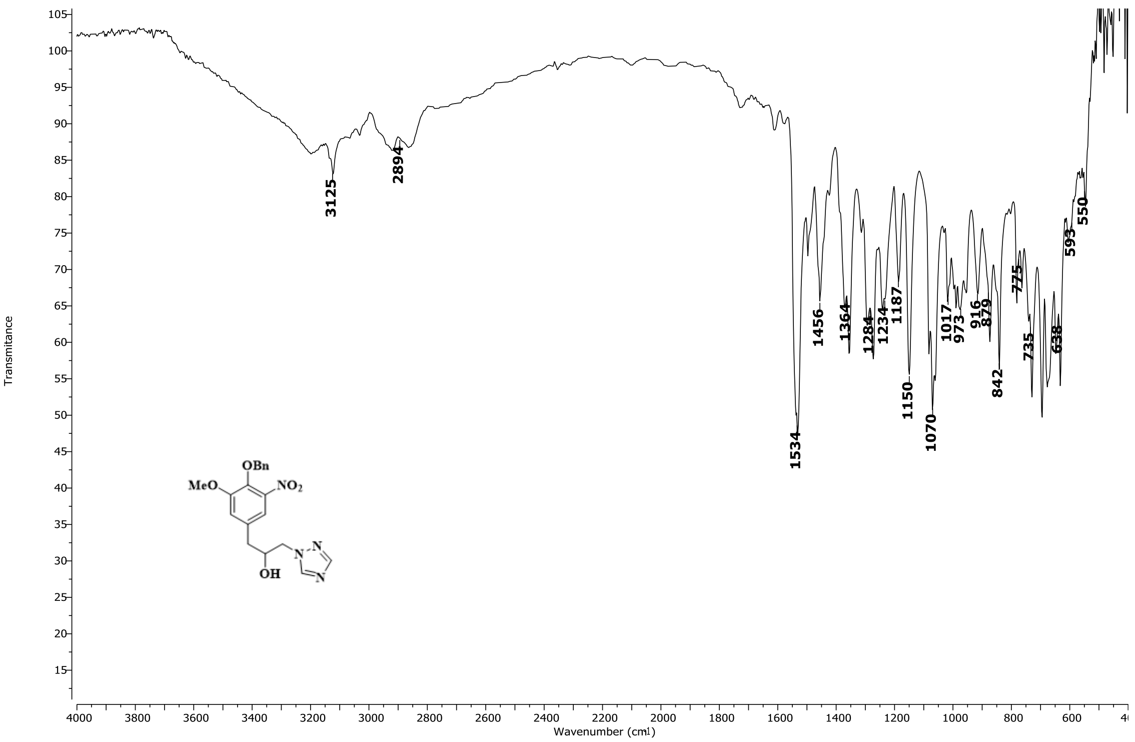

Figure 63: IR spectrum of compound **23**

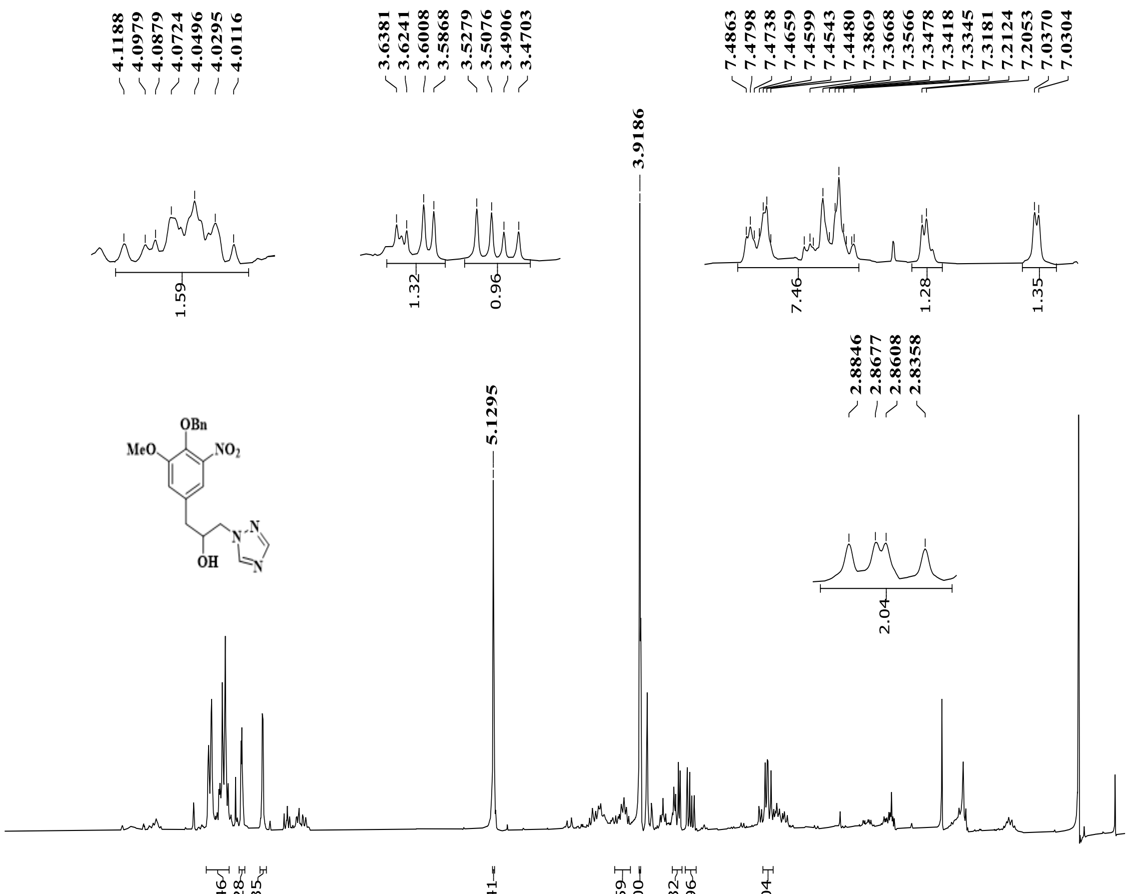

Figure 64: ^{1}H NMR spectrum of compound **23** ($CDCl_3$, 300Hz)

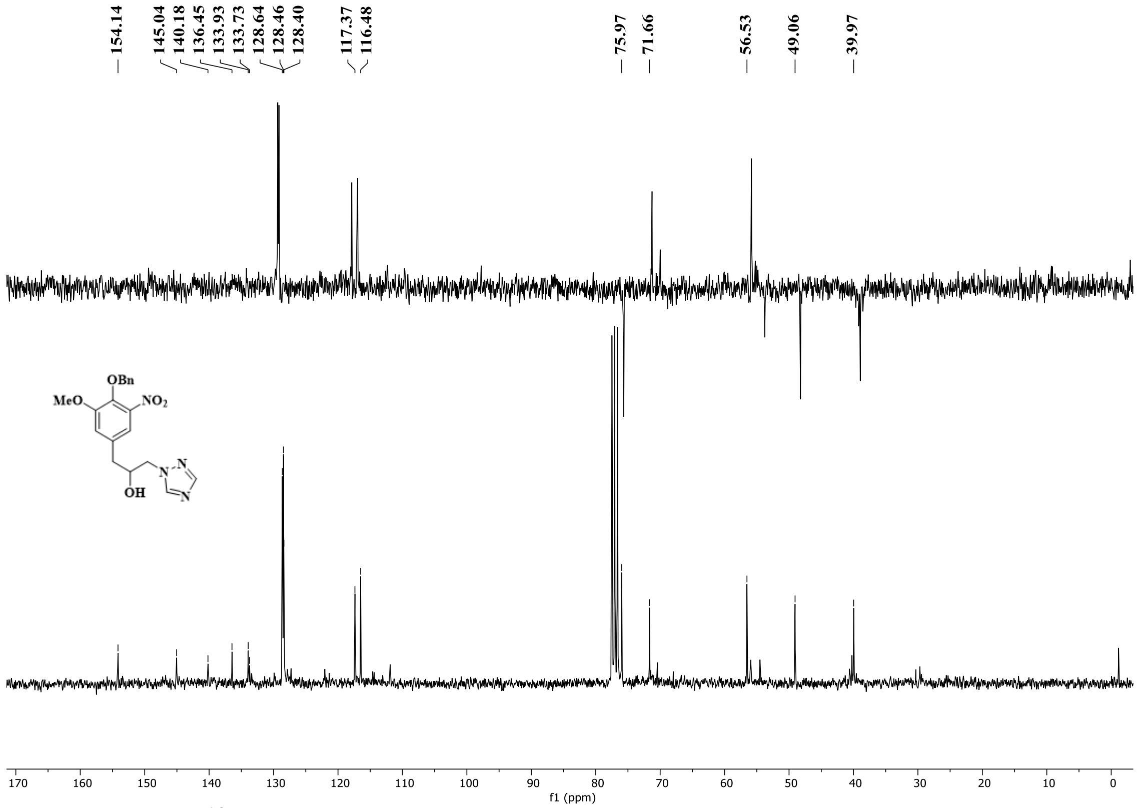

Figure 65: ^{13}C NMR spectrum and DEPT-135 of compound **23** ($CDCl_3$, 75Hz)

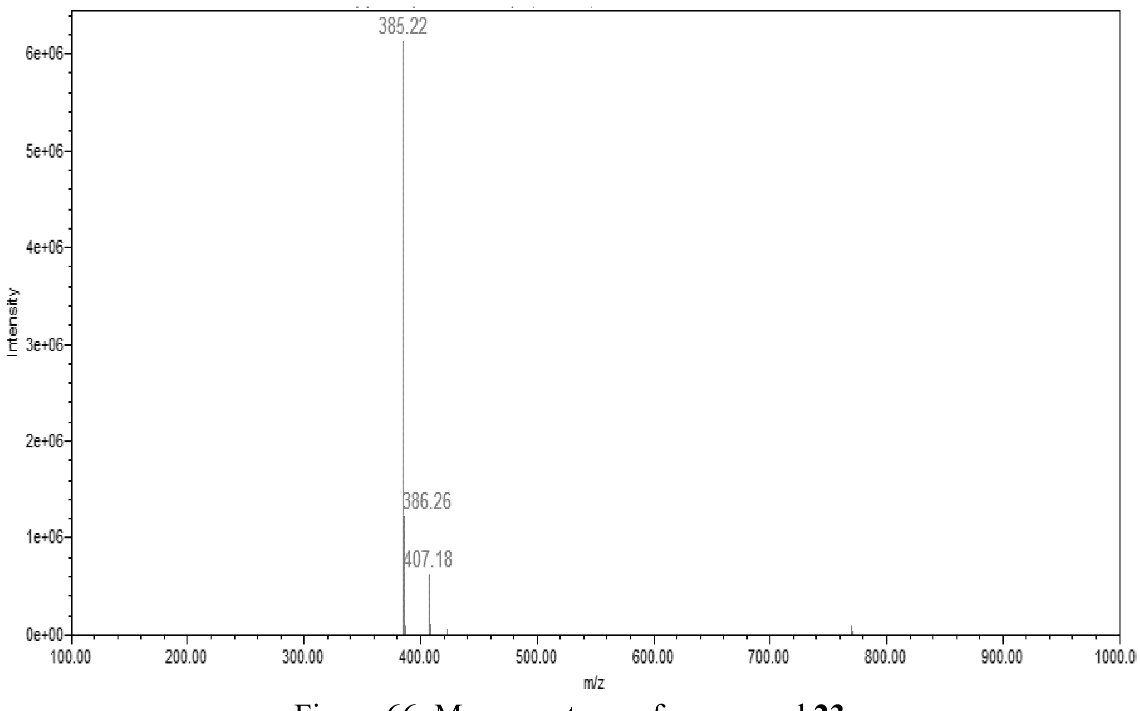

Figure 66: Mass spectrum of compound **23**

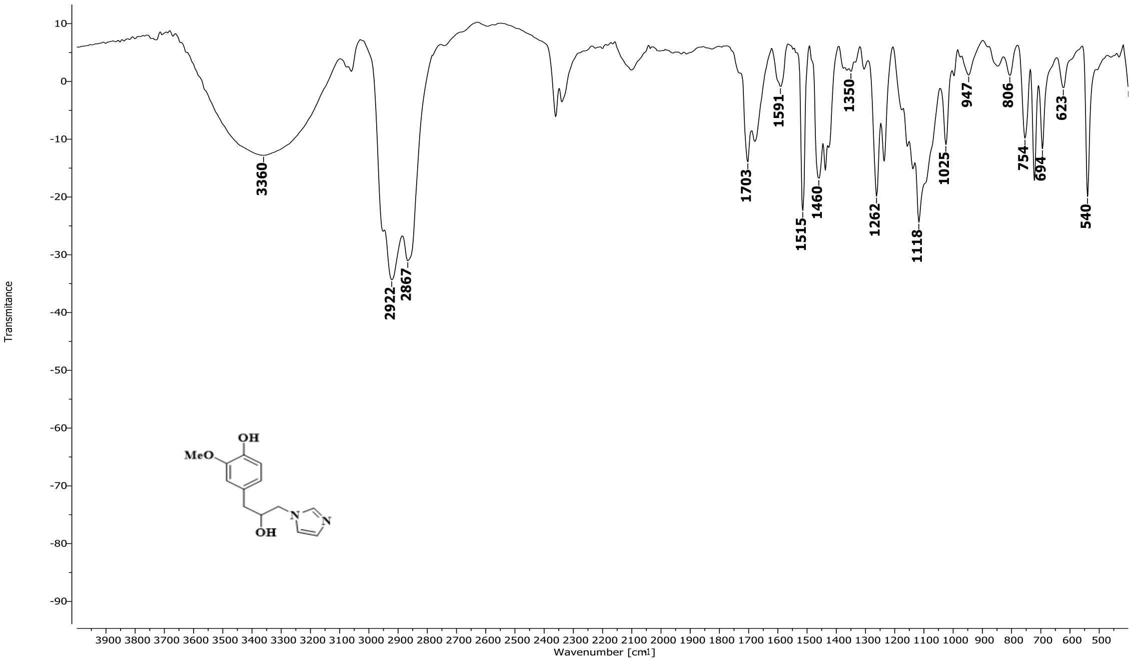

Figure 67: IR spectrum of compound **24**

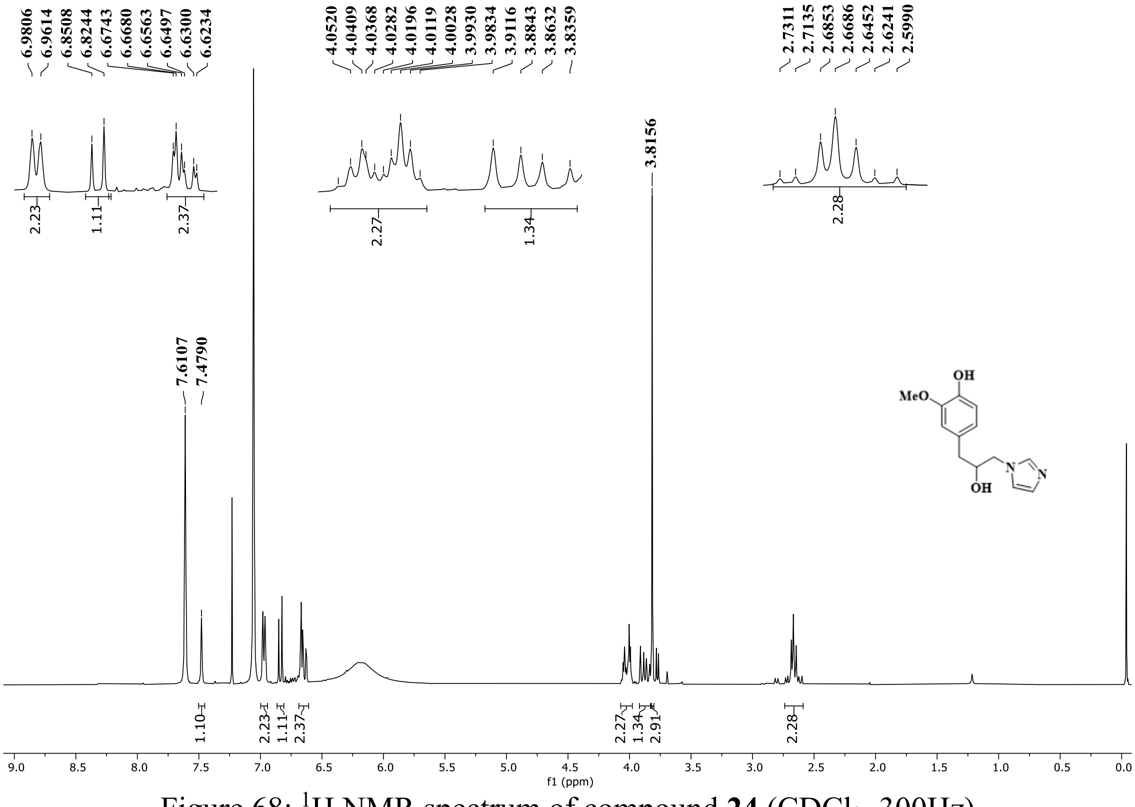

Figure 68: ^{1}H NMR spectrum of compound **24** ($CDCl_3$, 300Hz)

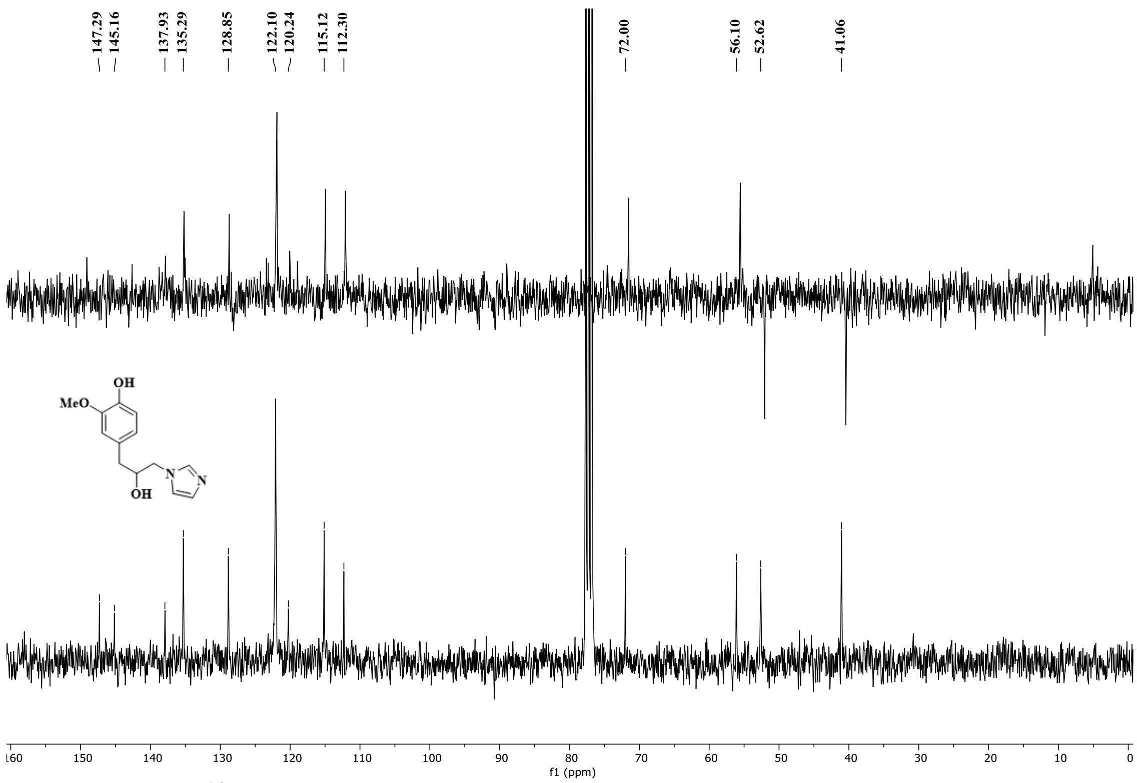

Figure 69: ^{13}C NMR spectrum and DEPT-135 of compound **24** ($CDCl_3$, 75Hz)

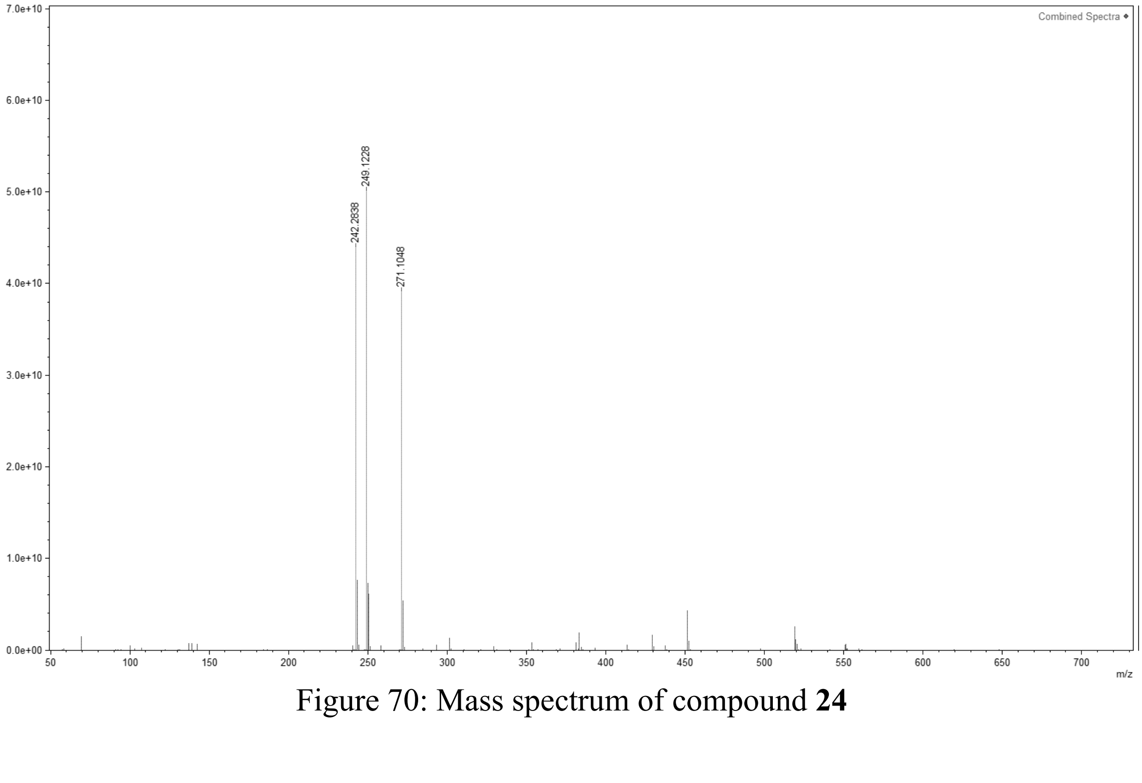

Figure 70: Mass spectrum of compound **24**

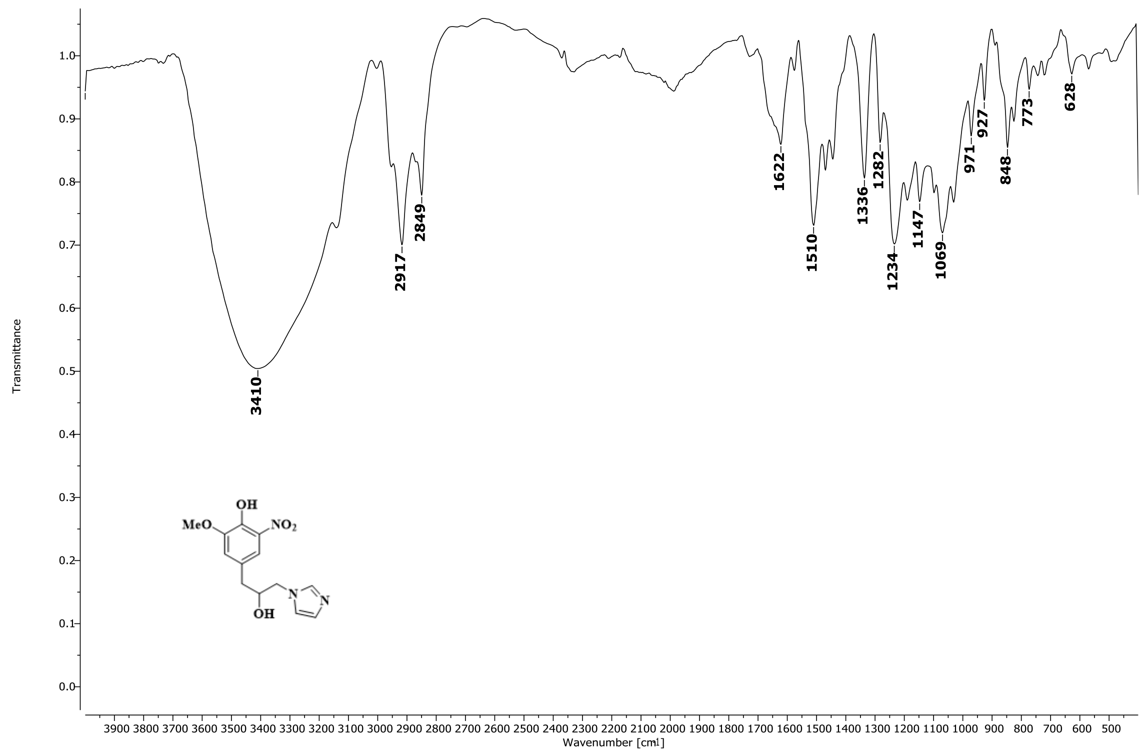

Figure 71: IR spectrum of compound **25**

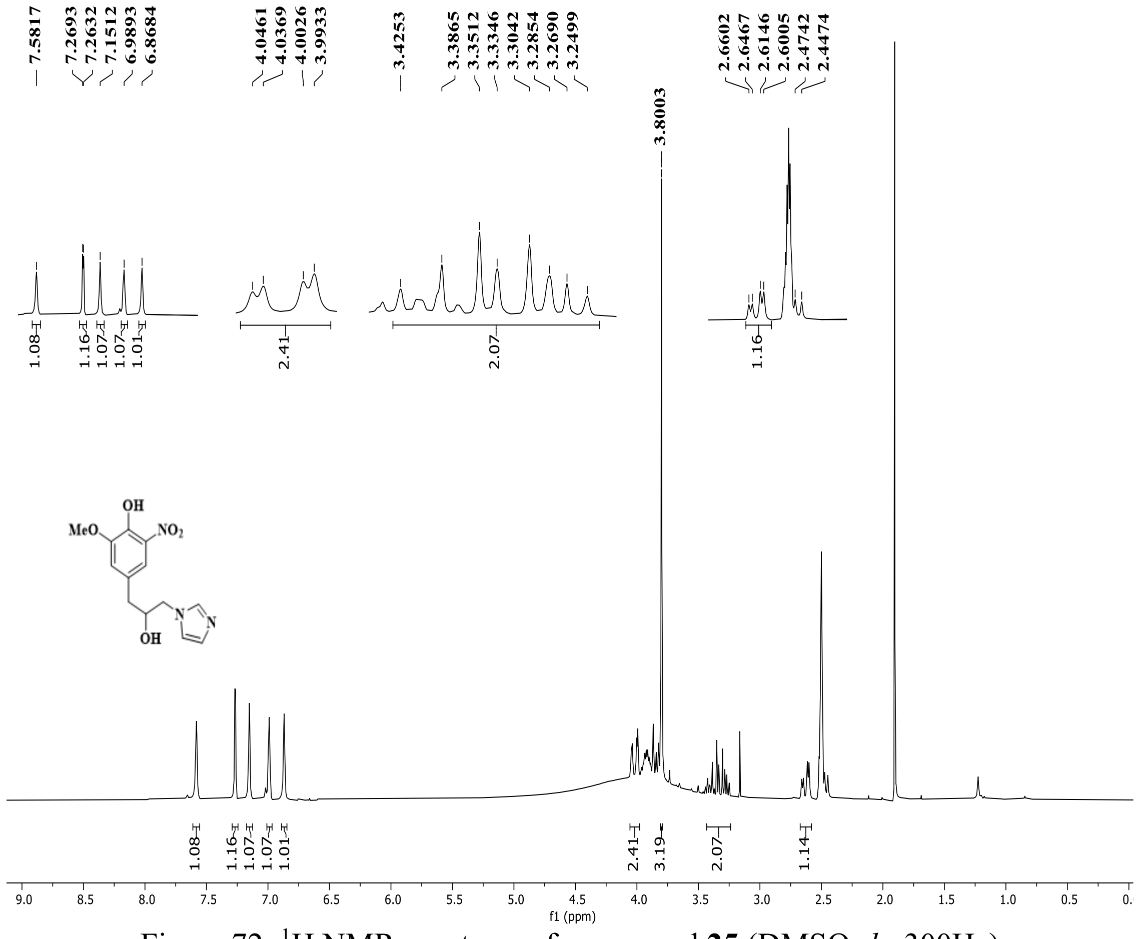

Figure 72: ^{1}H NMR spectrum of compound **25** (DMSO-d_6, 300Hz)

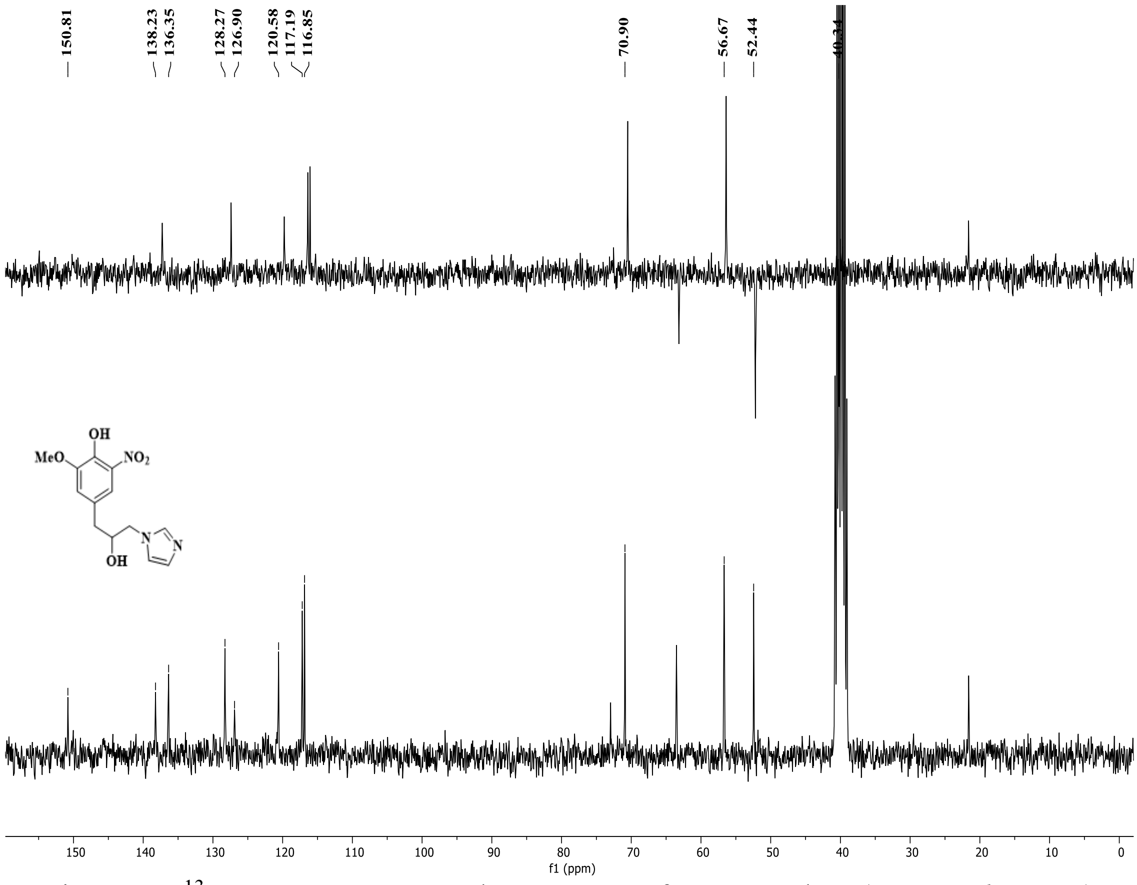

Figure 73: ^{13}C NMR spectrum and DEPT-135 of compound **25** (DMSO-d_6, 75Hz)

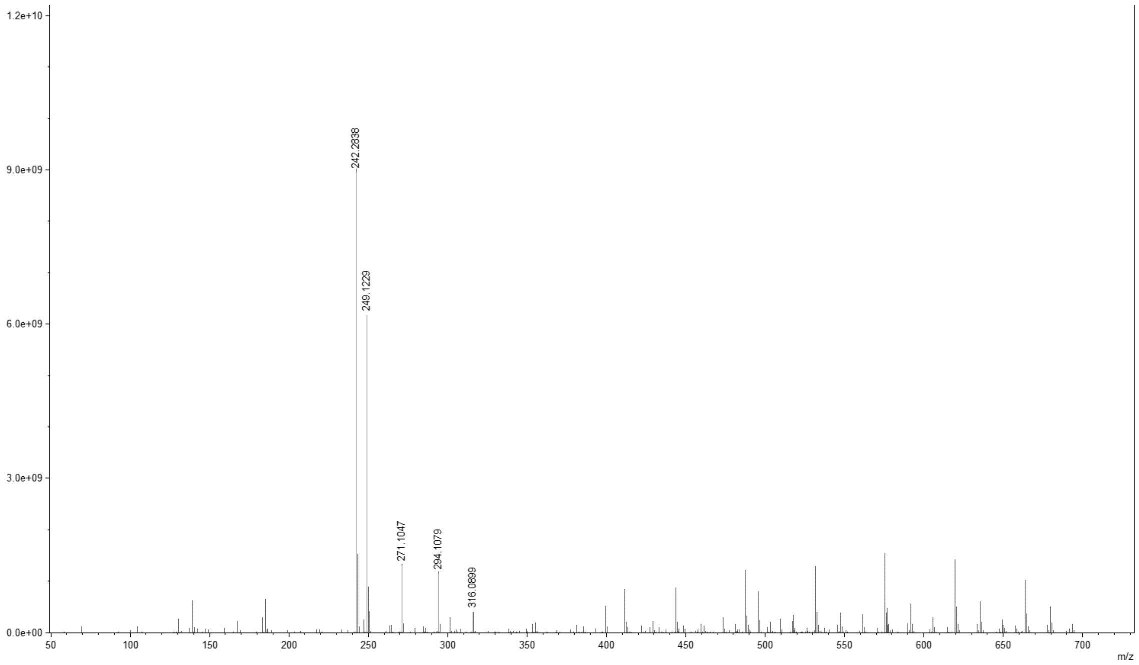

Figure 74: Mass spectrum of compound **25**

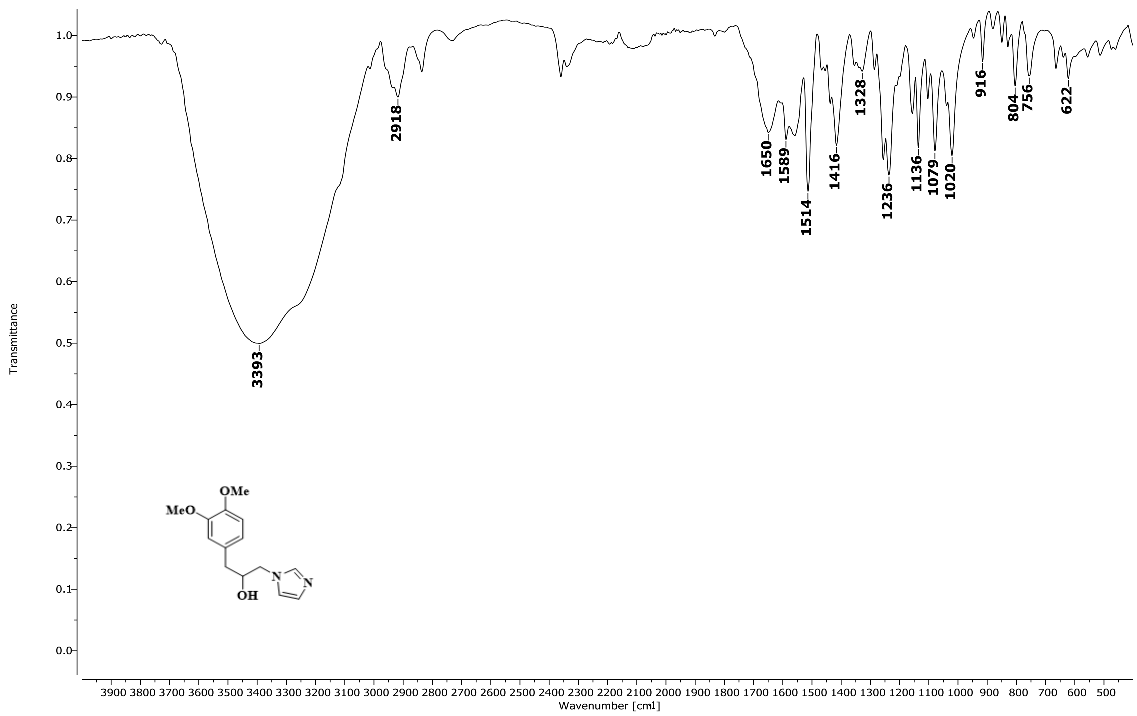

Figure 75: IR spectrum of compound **26**

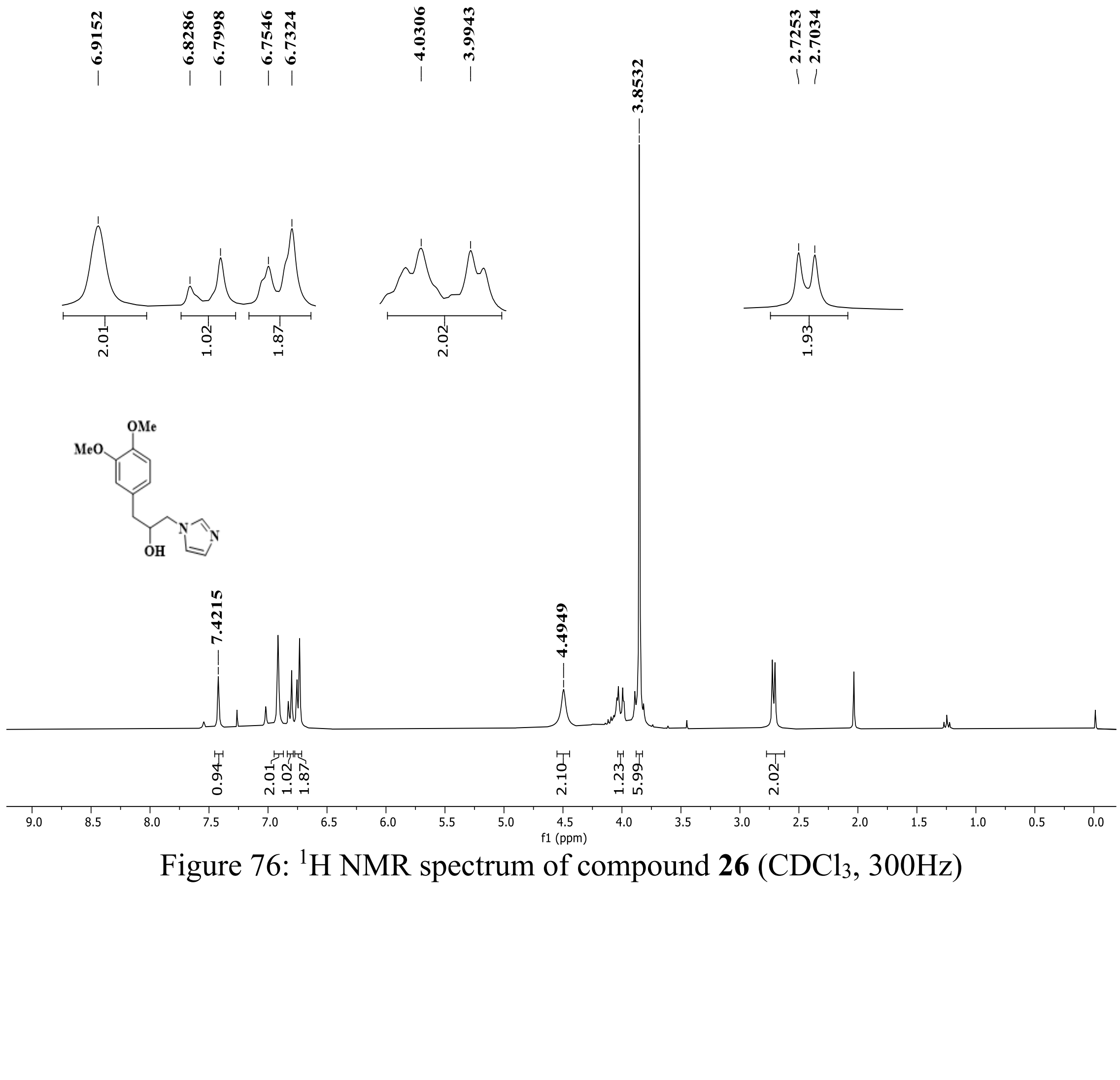

Figure 76: ^{1}H NMR spectrum of compound **26** ($CDCl_3$, 300Hz)

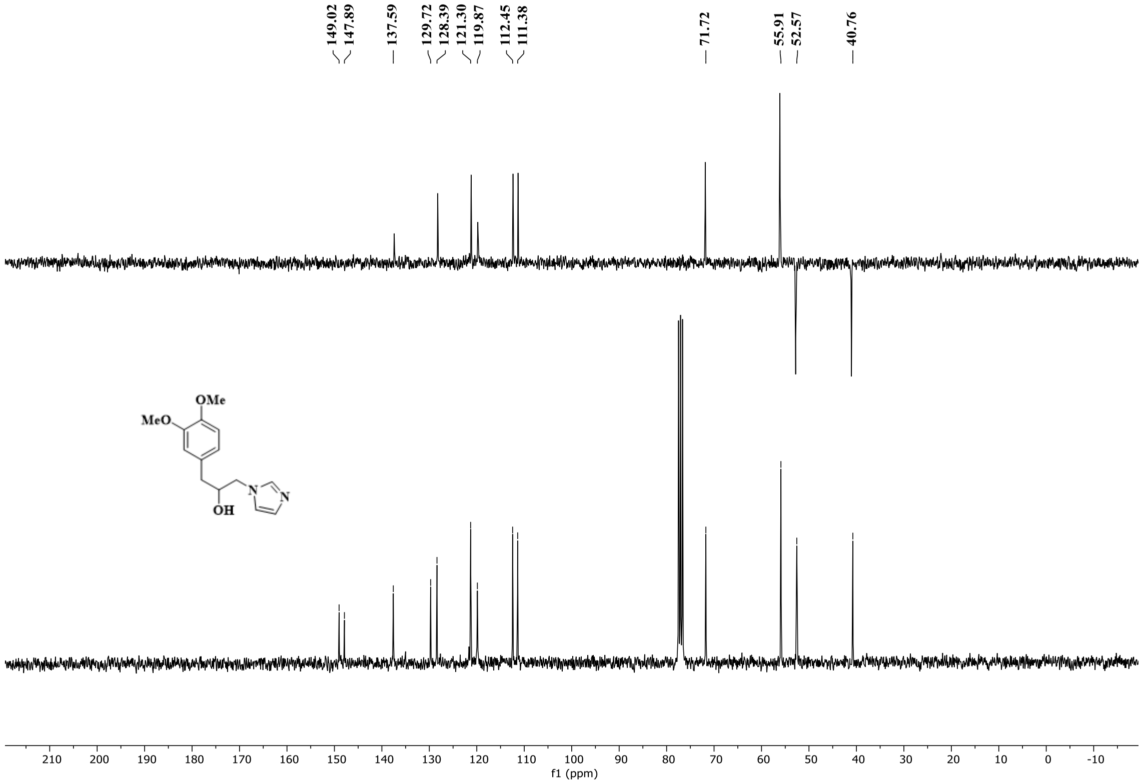

Figure 77: ^{13}C NMR spectrum and DEPT-135 of compound **26** ($CDCl_3$, 75Hz)

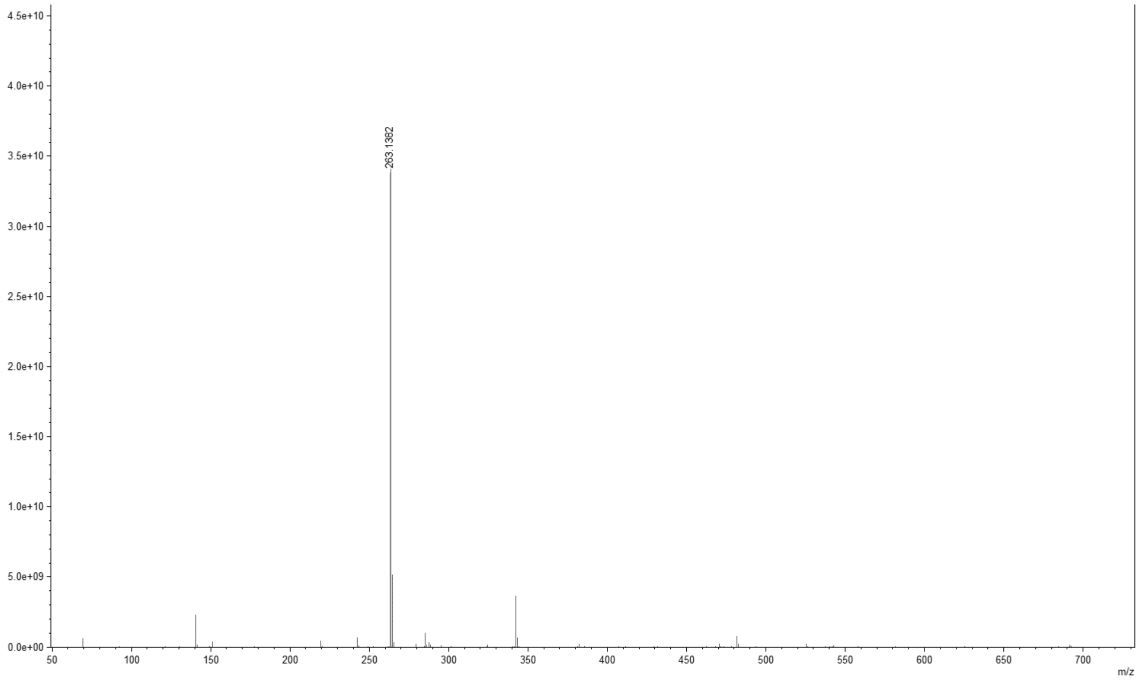

Figure 78: Mass spectrum of compound **26**

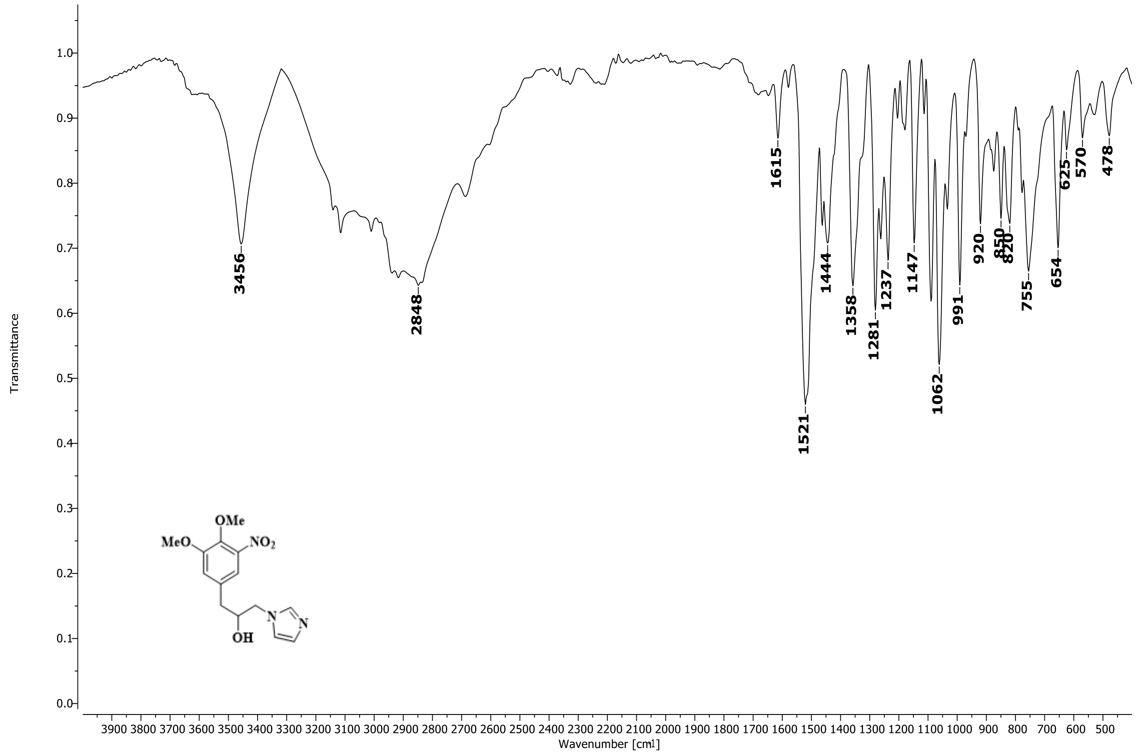

Figure 79: IR spectrum of compound **27**

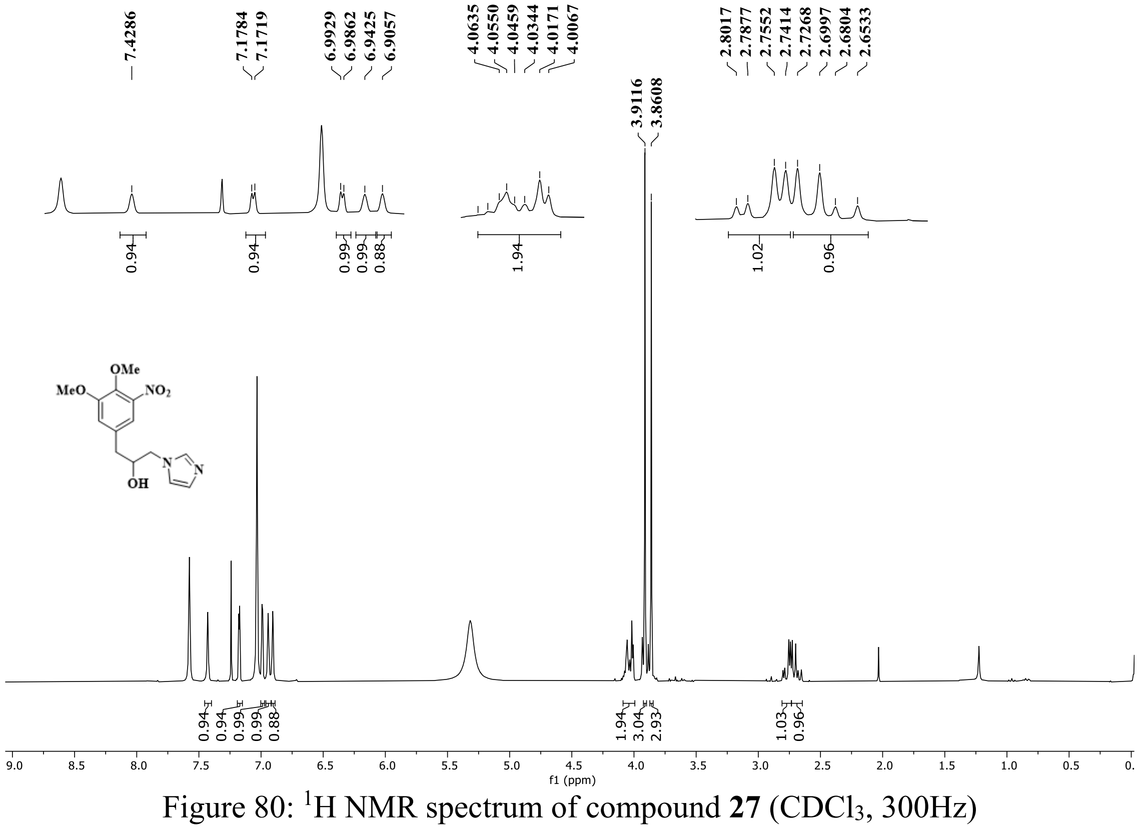

Figure 80: ^{1}H NMR spectrum of compound **27** ($CDCl_3$, 300Hz)

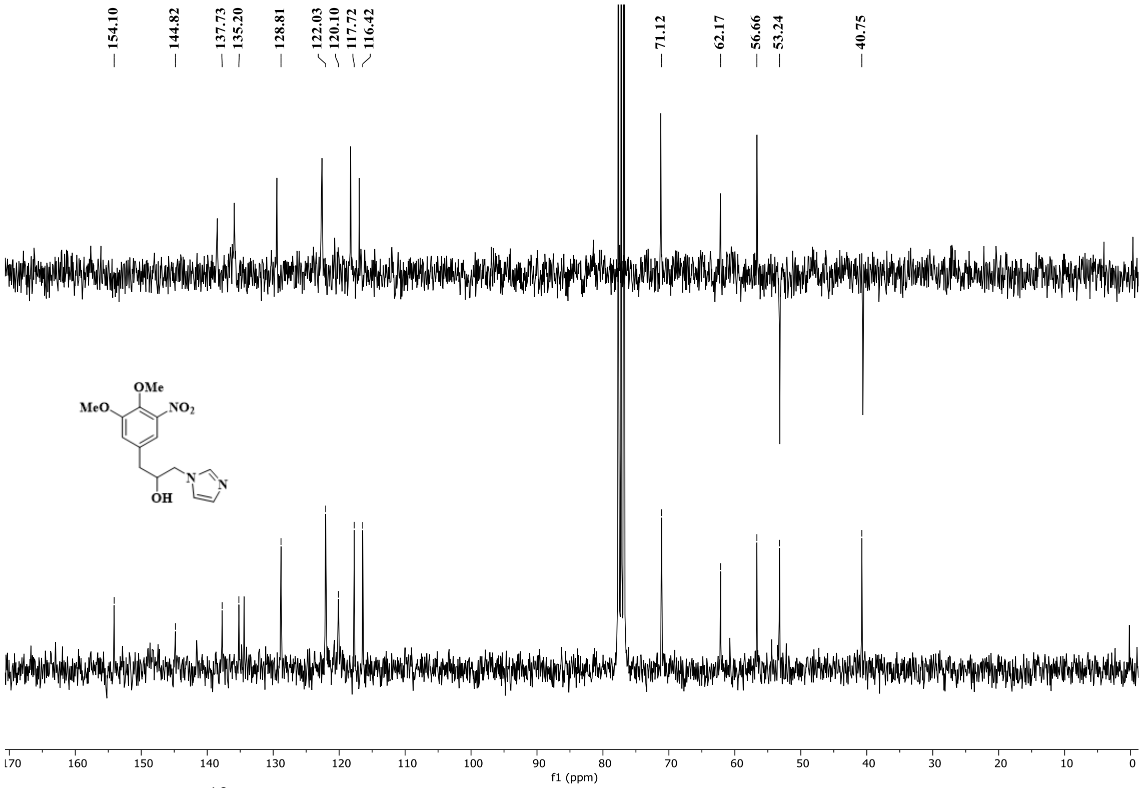

Figure 81: ^{13}C NMR spectrum and DEPT-135 of compound **27** ($CDCl_3$, 75Hz)

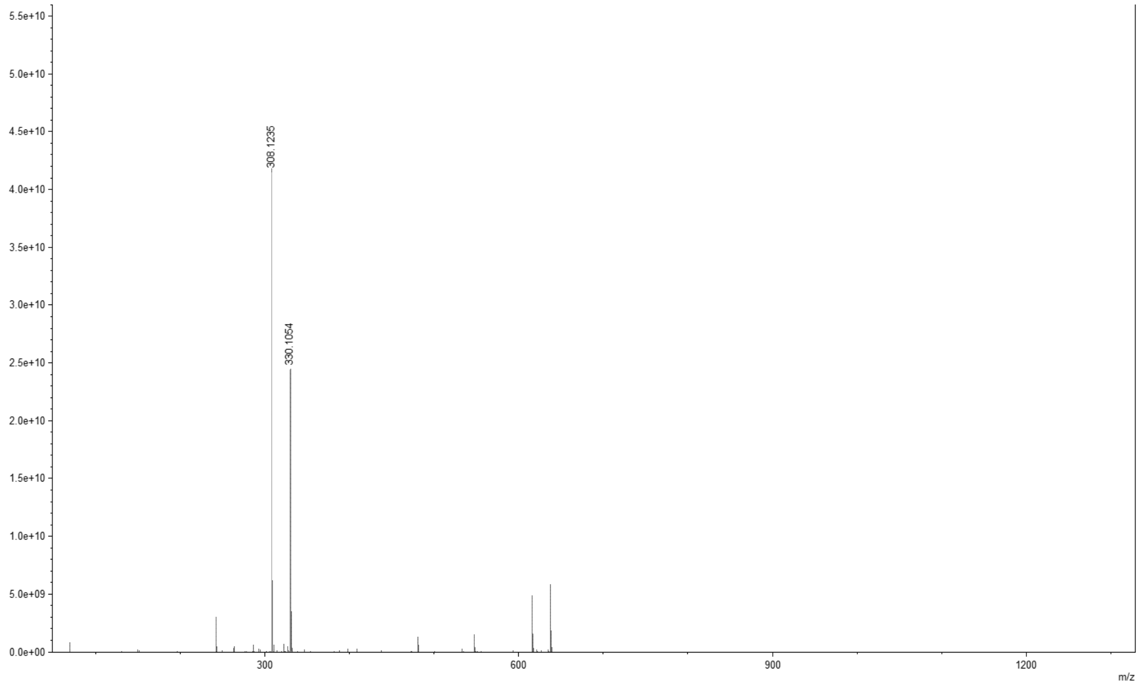

Figure 82: Mass spectrum of compound **27**

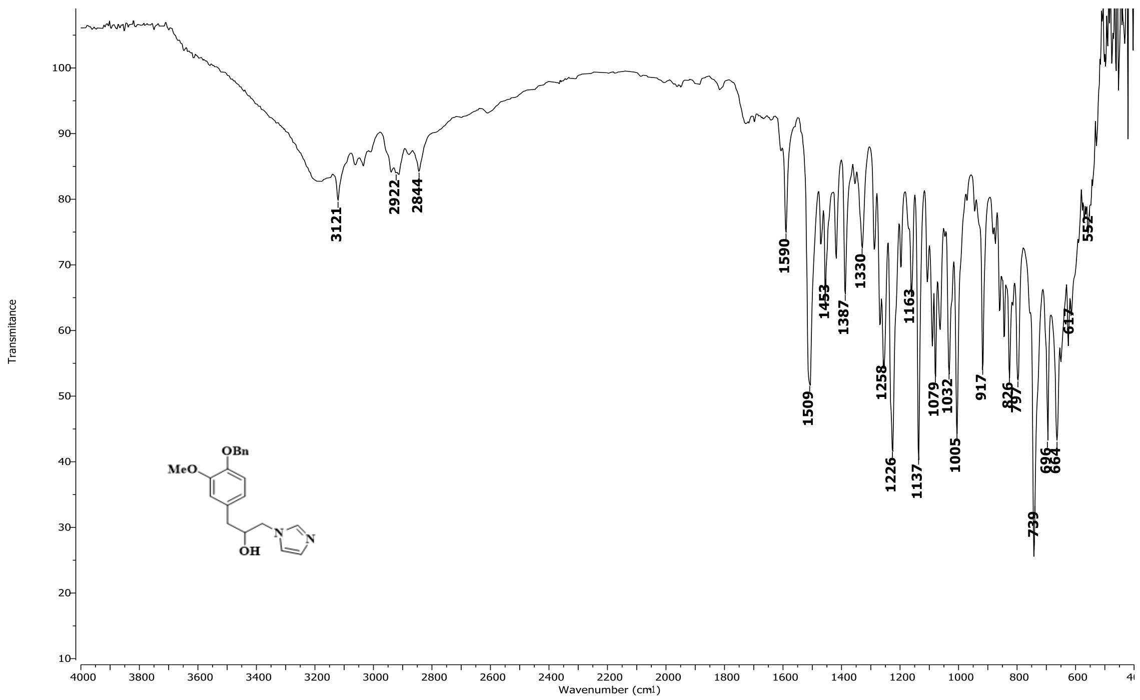

Figure 83: IR spectrum of compound **28**

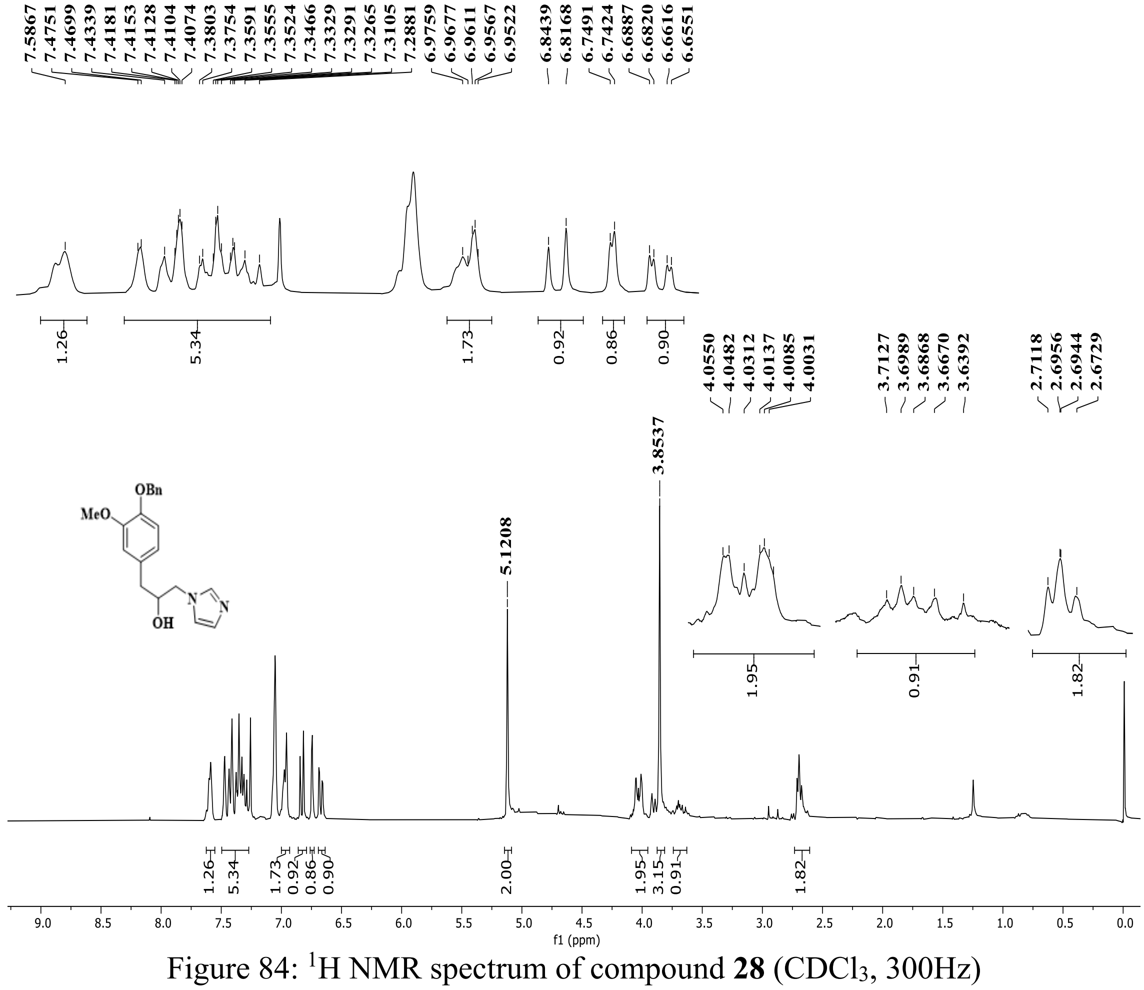

Figure 84: ^{1}H NMR spectrum of compound **28** ($CDCl_3$, 300Hz)

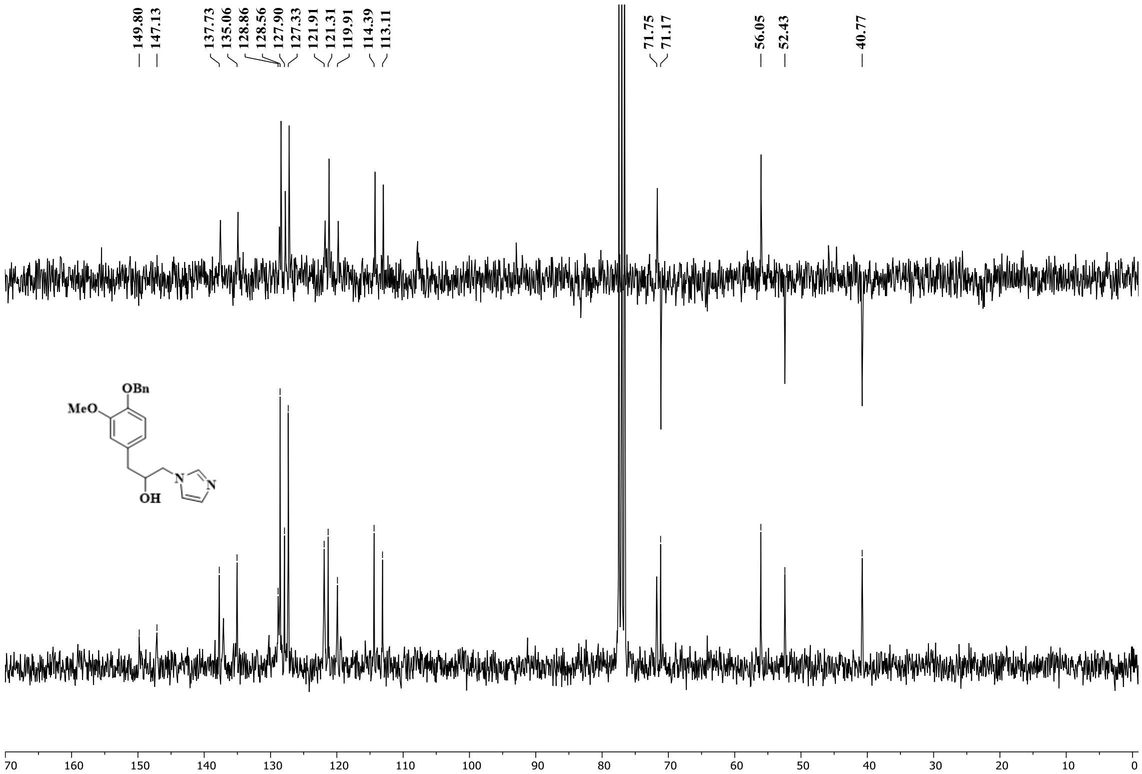

Figure 85: ^{13}C NMR spectrum and DEPT-135 of compound **28** ($CDCl_3$, 75Hz)

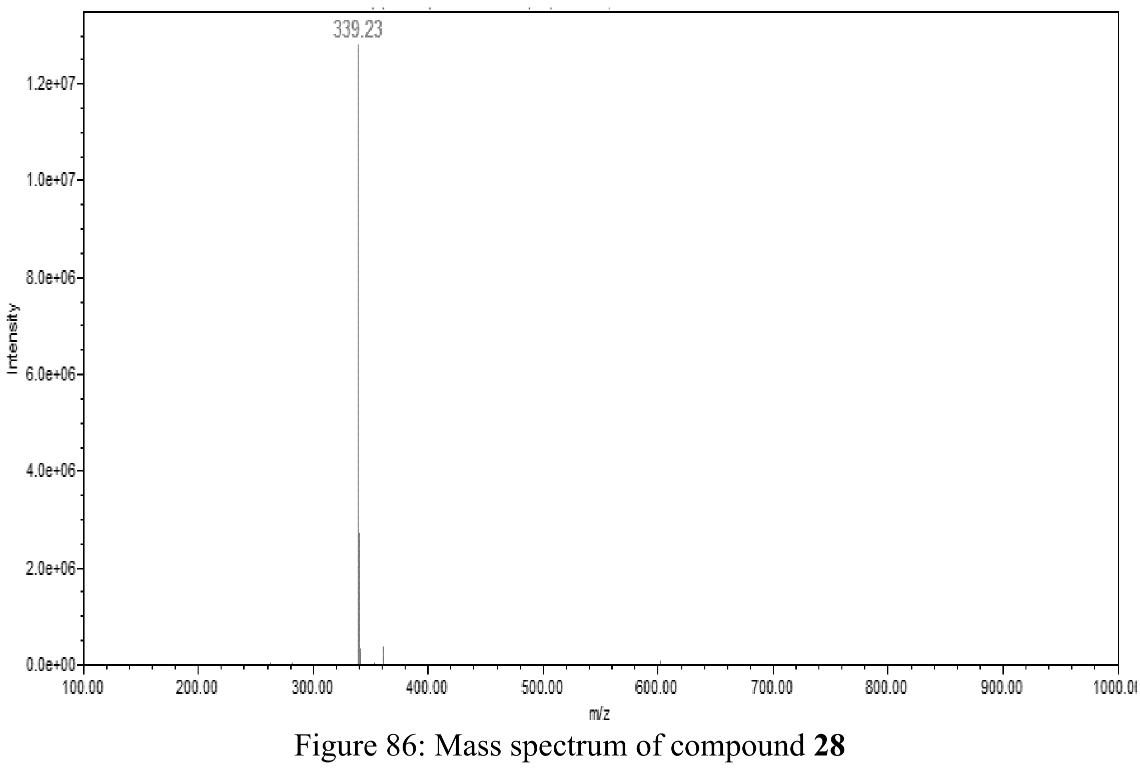

Figure 86: Mass spectrum of compound **28**

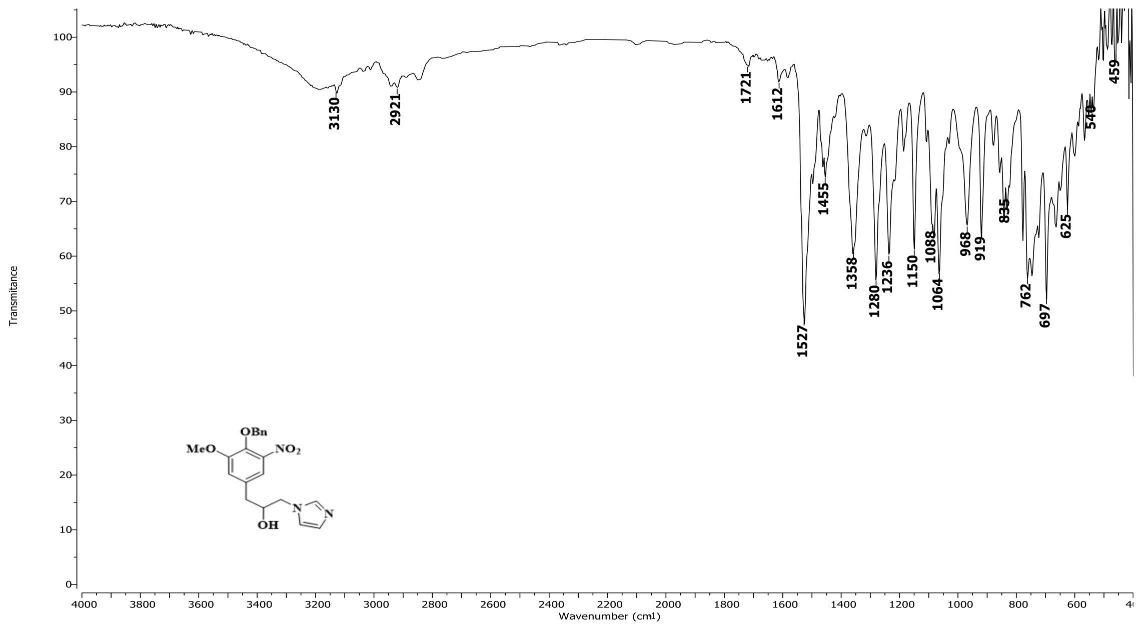

Figure 87: IR spectrum of compound **29**

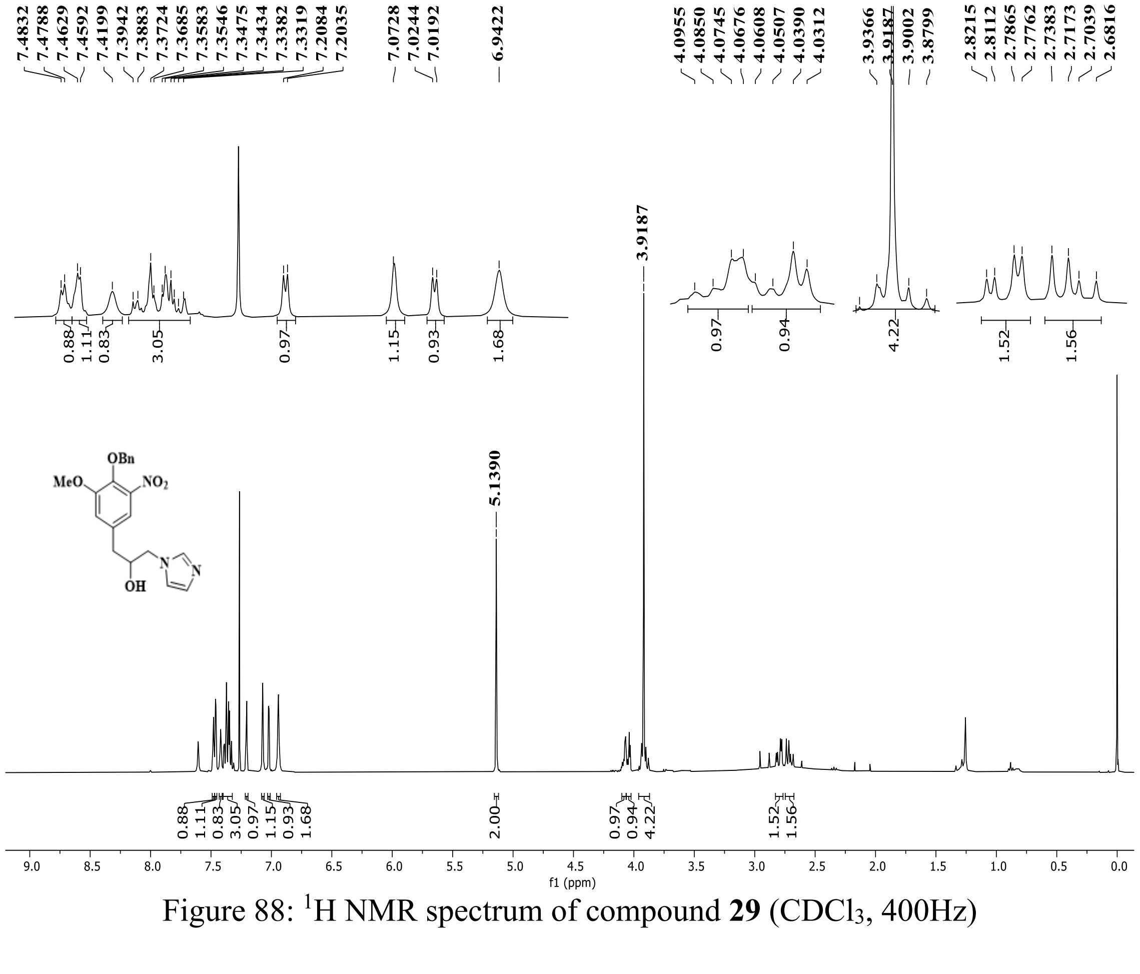

Figure 88: ^{1}H NMR spectrum of compound **29** ($CDCl_3$, 400Hz)

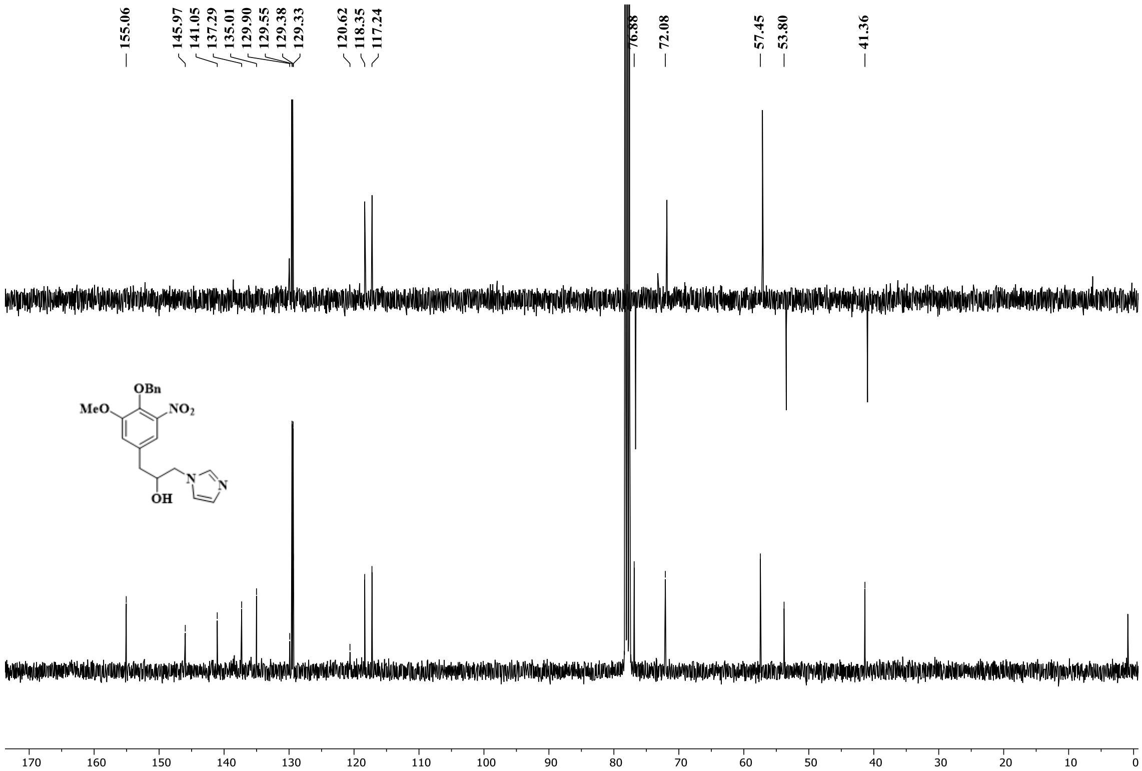

Figure 89: ^{13}C NMR spectrum and DEPT-135 of compound **29** ($CDCl_3$, 100Hz)

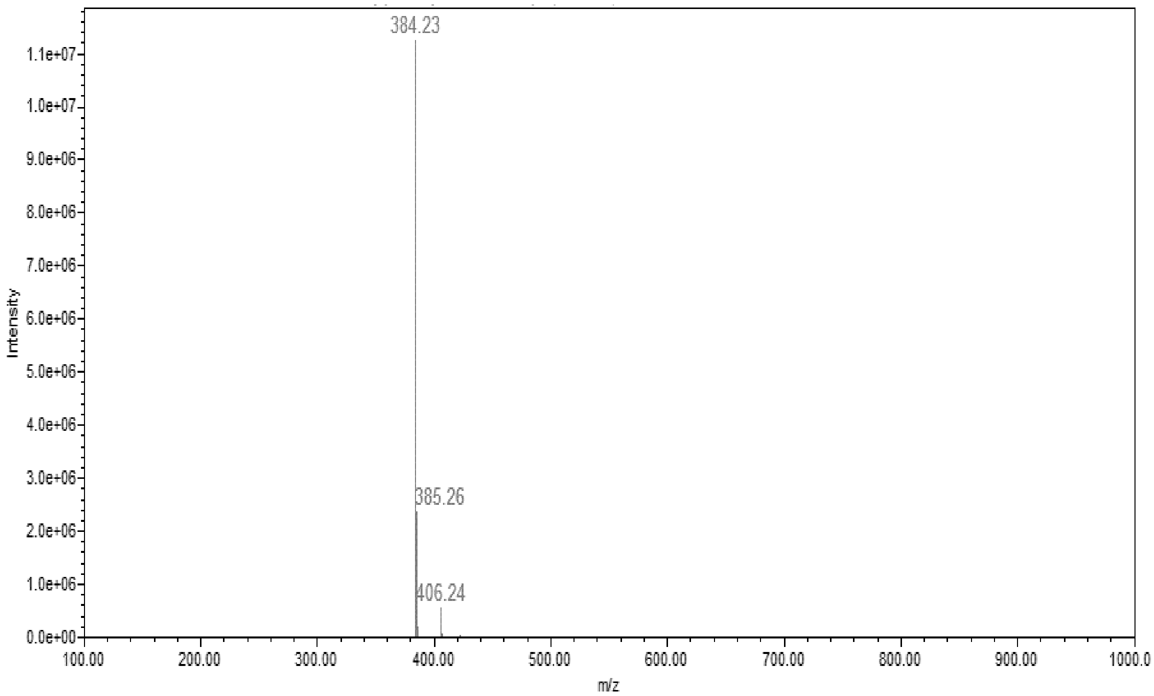

Figure 90: Mass spectrum of compound **29**

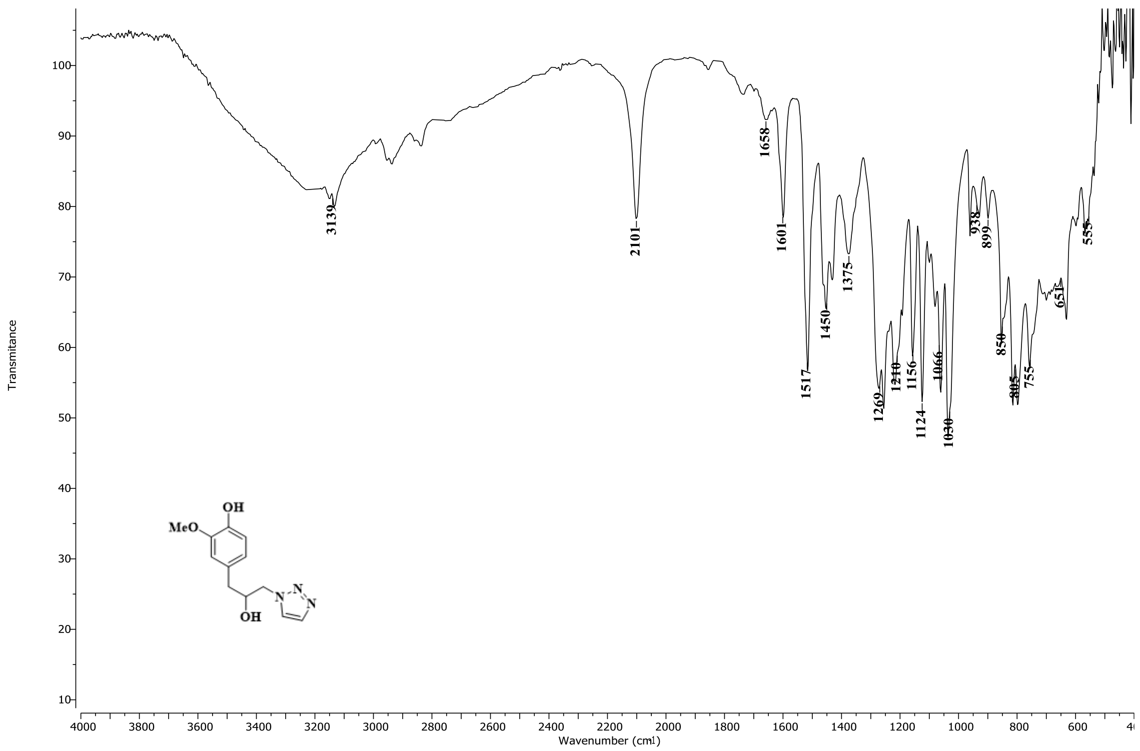

Figure 91: IR spectrum of compound **30**

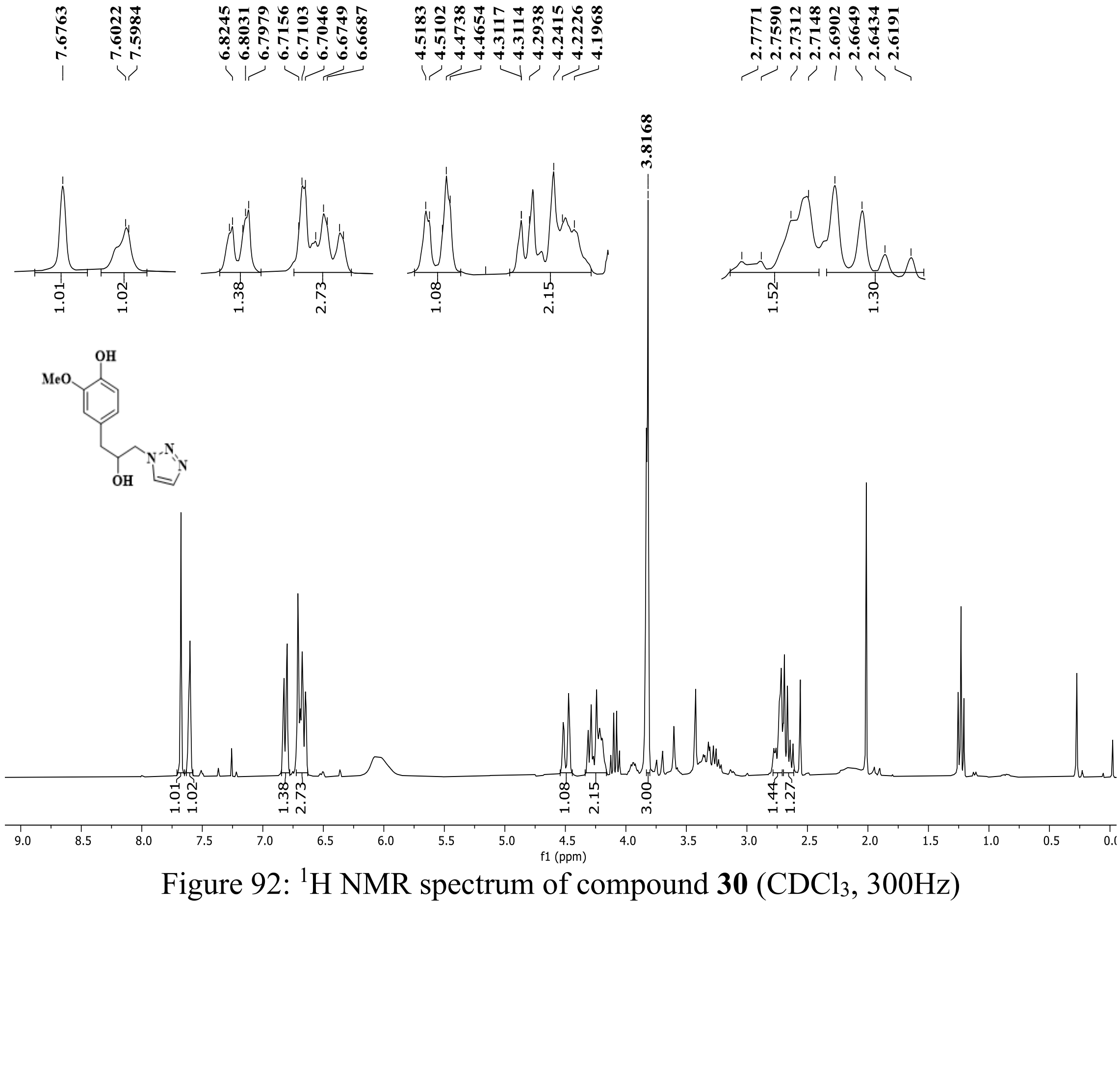

Figure 92: ^{1}H NMR spectrum of compound **30** ($CDCl_3$, 300Hz)

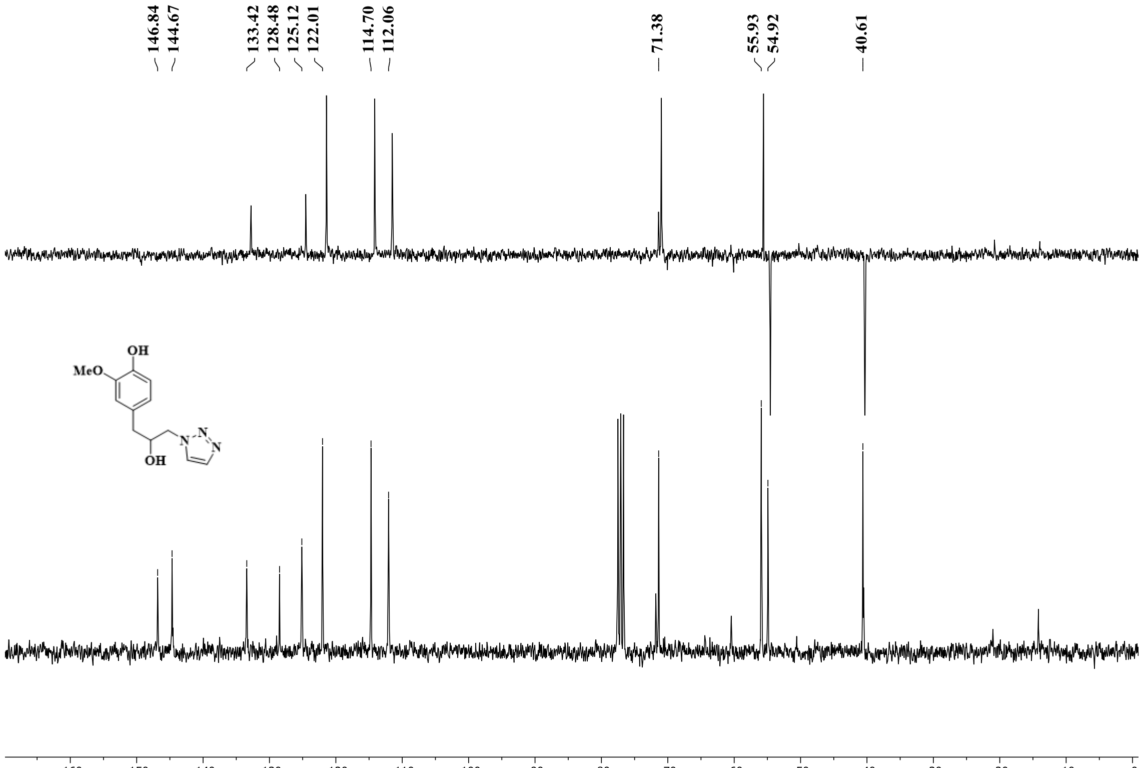

Figure 93: ^{13}C NMR spectrum and DEPT-135 of compound **30** ($CDCl_3$, 75Hz)

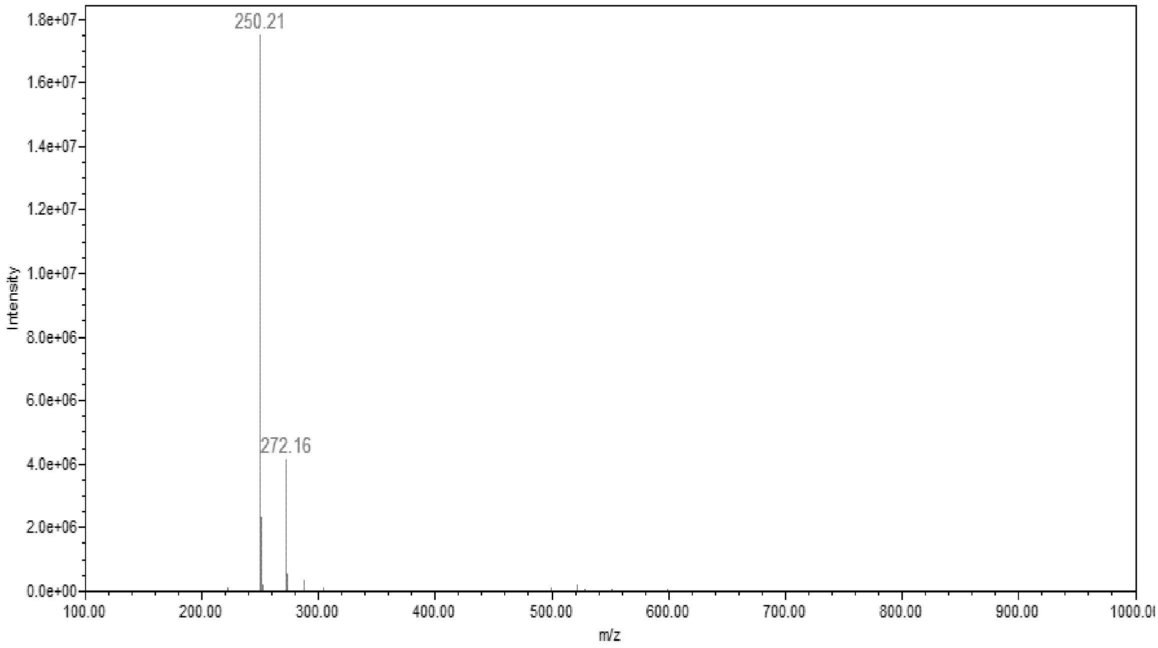

Figure 94: Mass spectrum of compound **30**

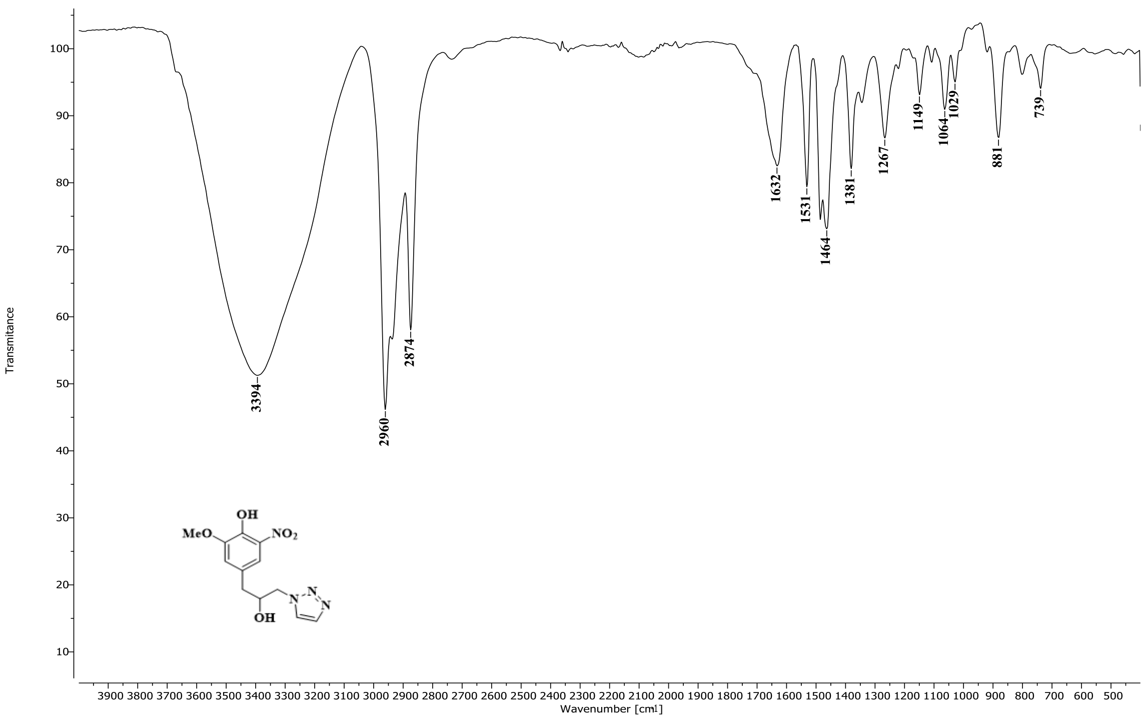

Figure 95: IR spectrum of compound **31**

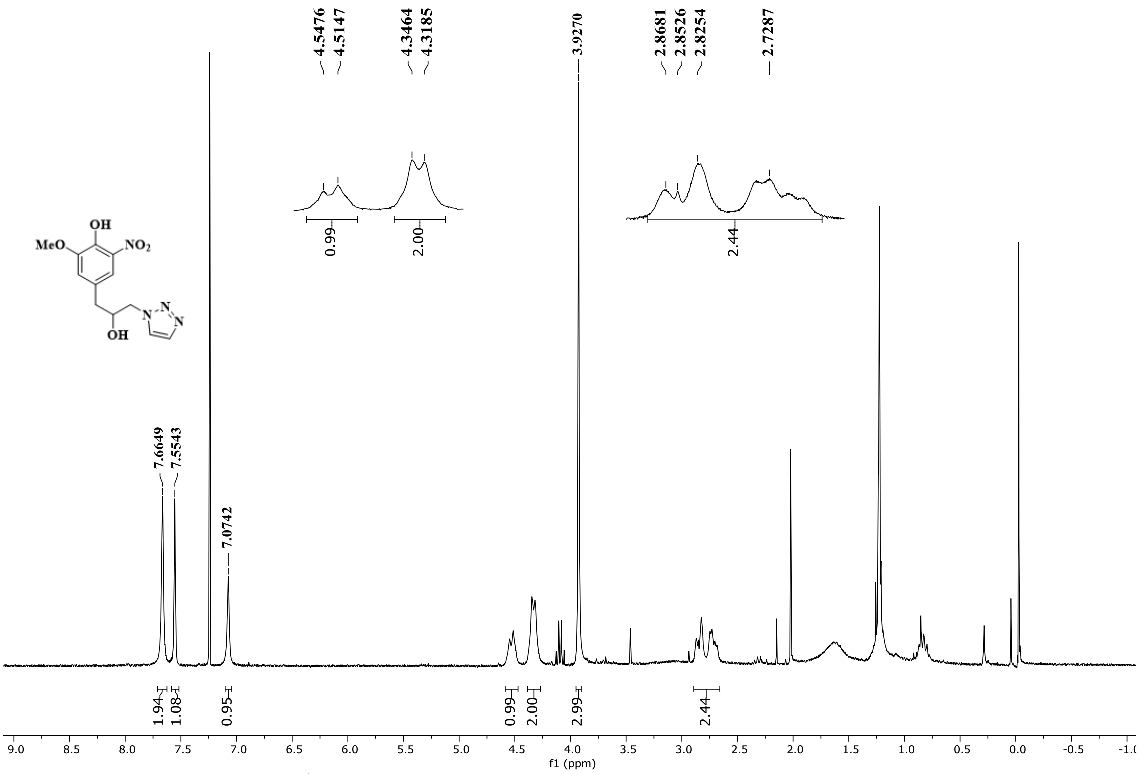

Figure 96: ^{1}H NMR spectrum of compound **31** ($CDCl_3$, 300Hz)

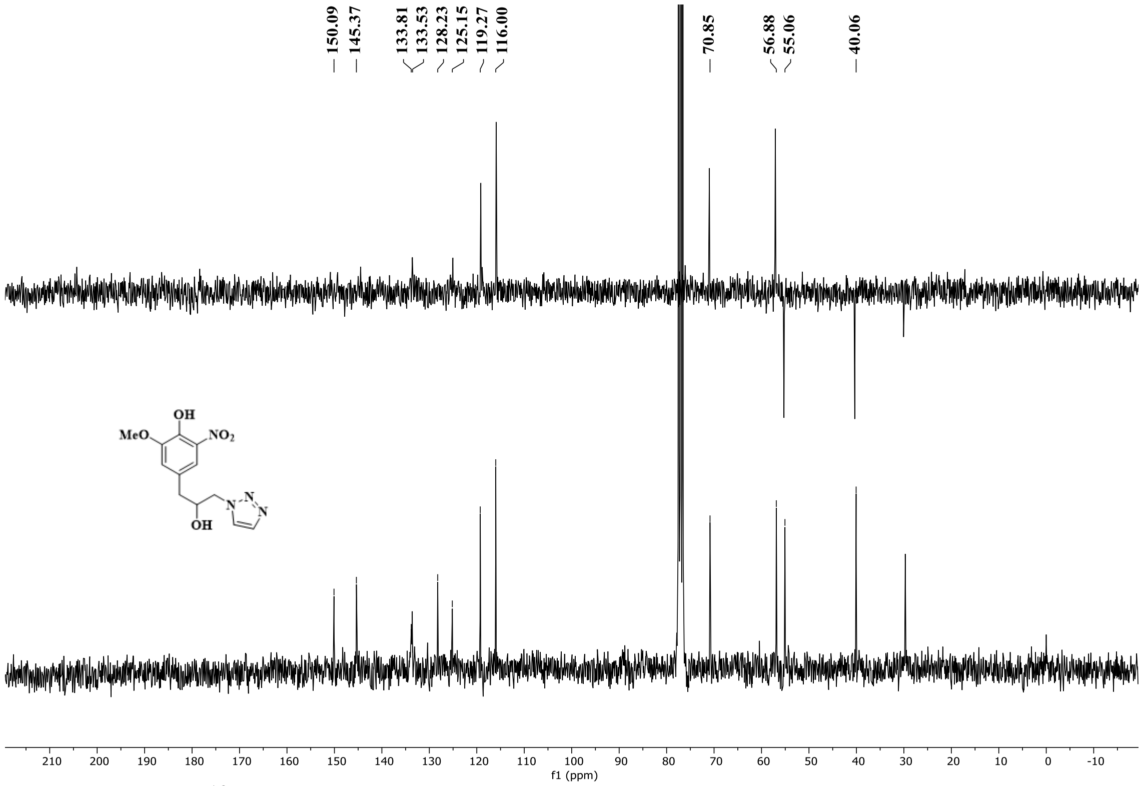

Figure 97: ^{13}C NMR spectrum and DEPT-135 of compound **31** ($CDCl_3$, 75Hz)

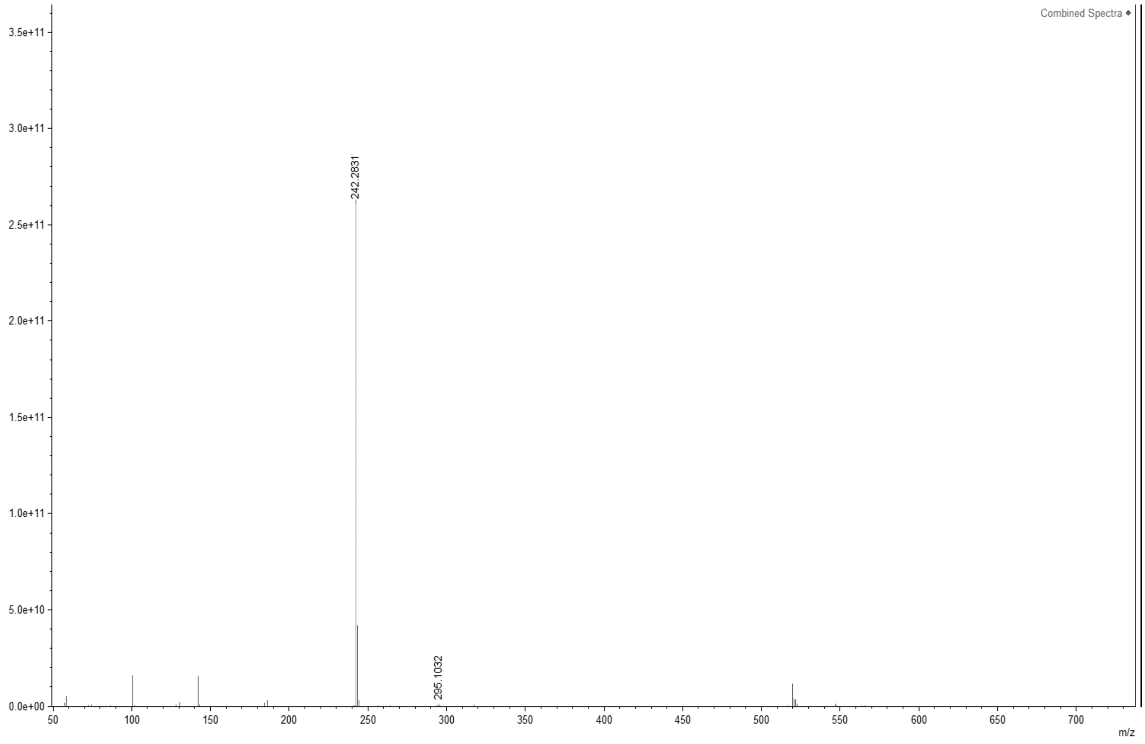

Figure 98: Mass spectrum of compound **31**

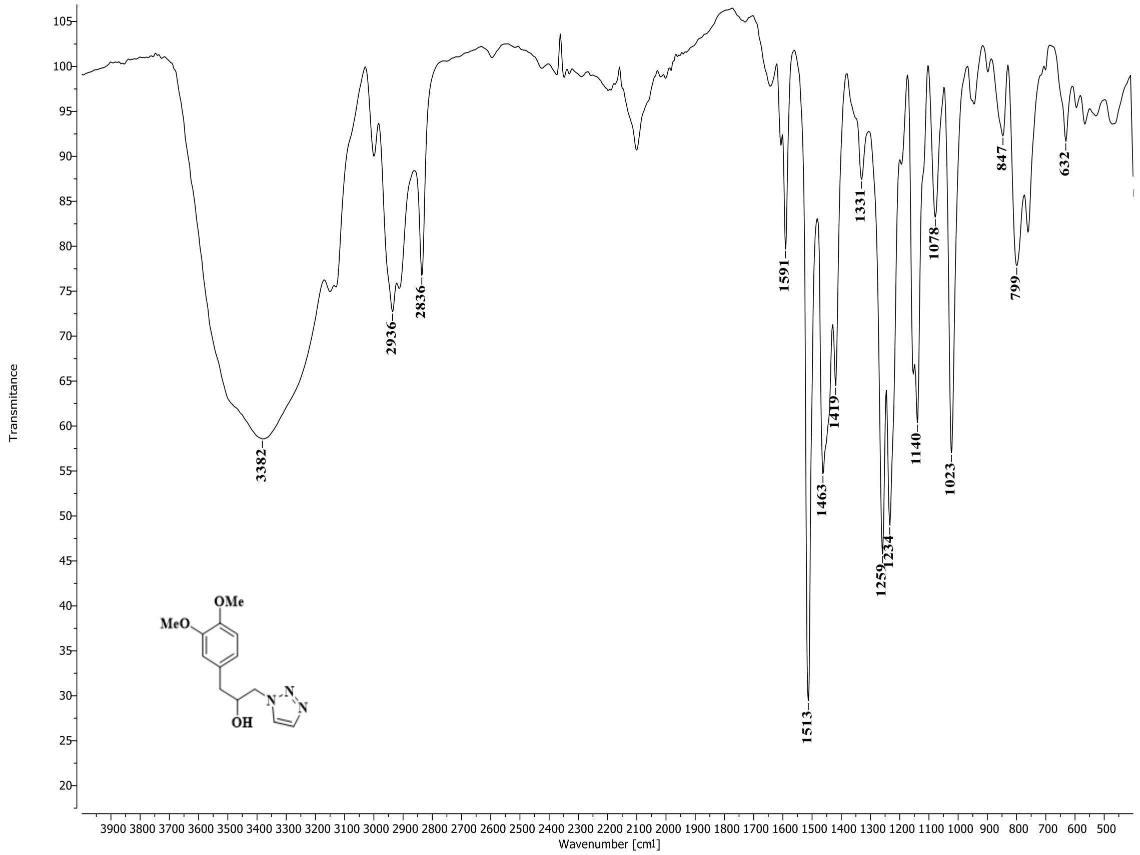

Figure 99: IR spectrum of compound **32**

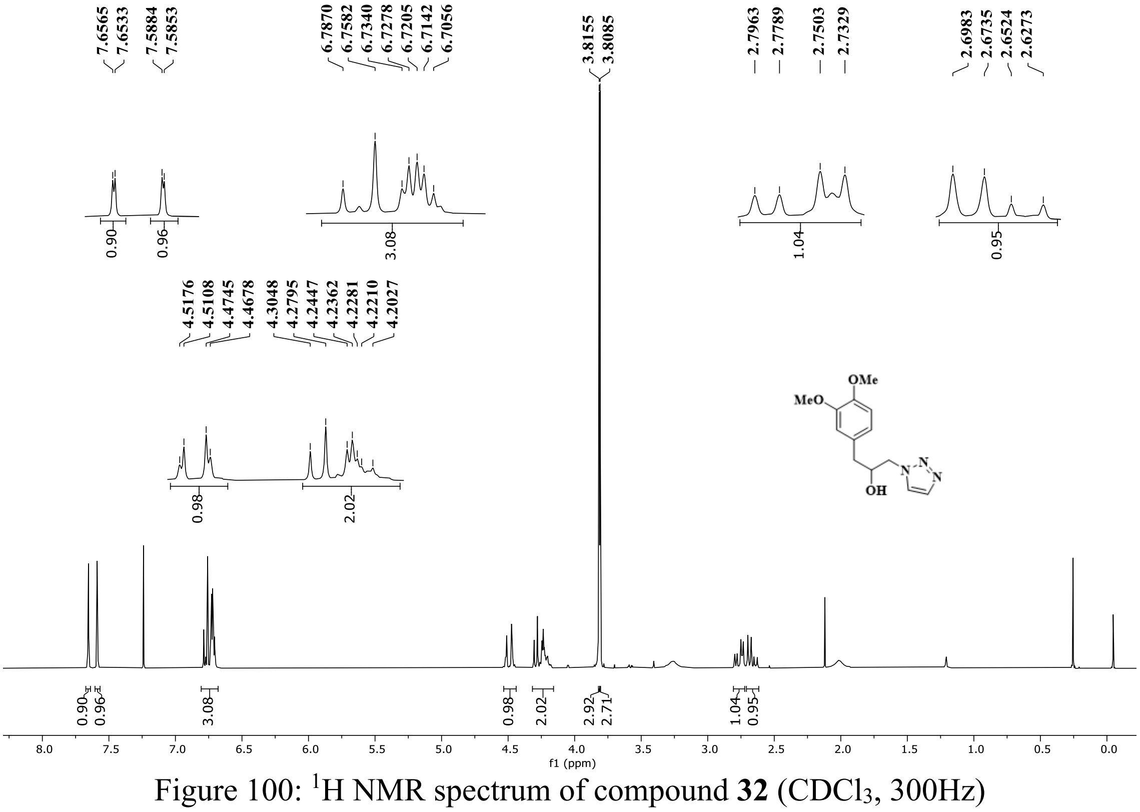

Figure 100: ^{1}H NMR spectrum of compound **32** ($CDCl_3$, 300Hz)

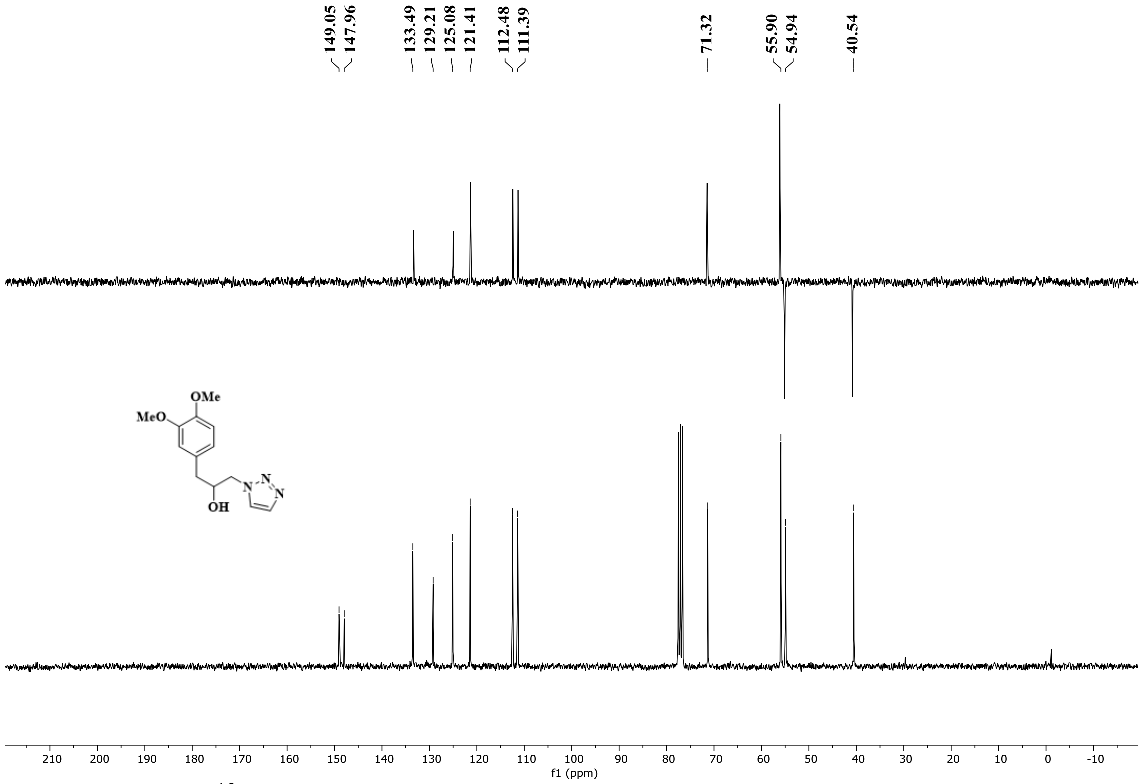

Figure 101: ^{13}C NMR spectrum and DEPT-135 of compound **32** ($CDCl_3$, 75Hz)

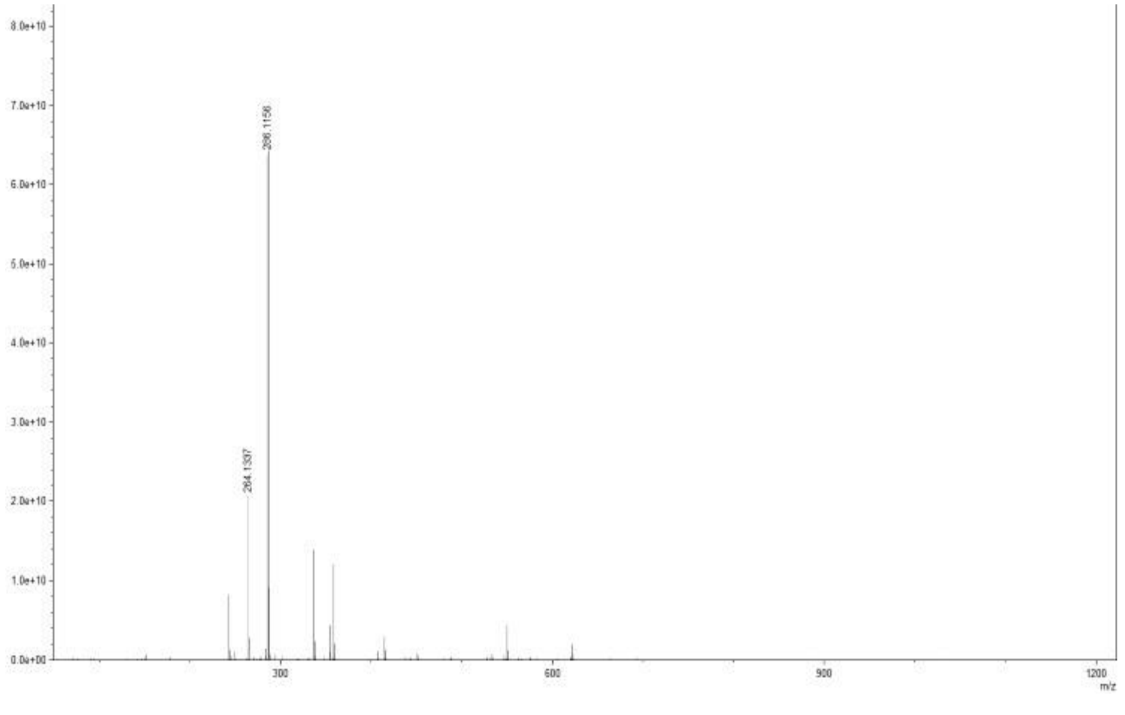

Figure 102: Mass spectrum of compound **32**

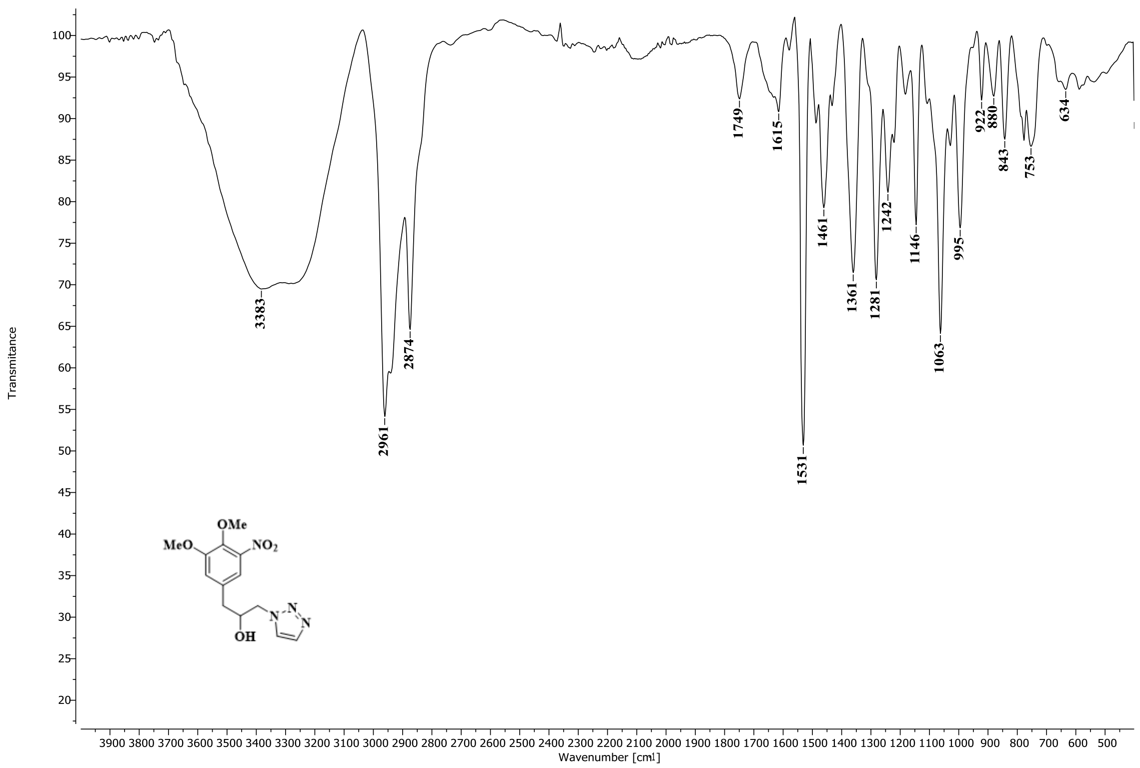

Figure 103: IR spectrum of compound **33**

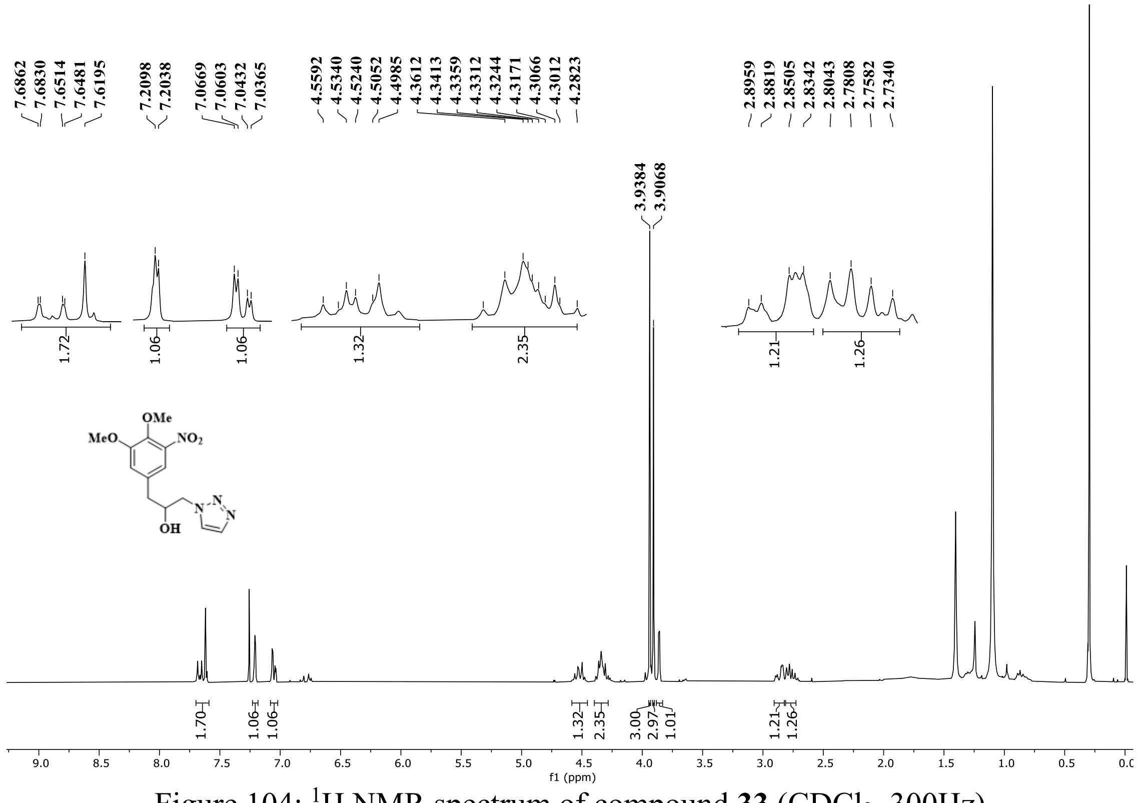

Figure 104: ^{1}H NMR spectrum of compound **33** ($CDCl_3$, 300Hz)

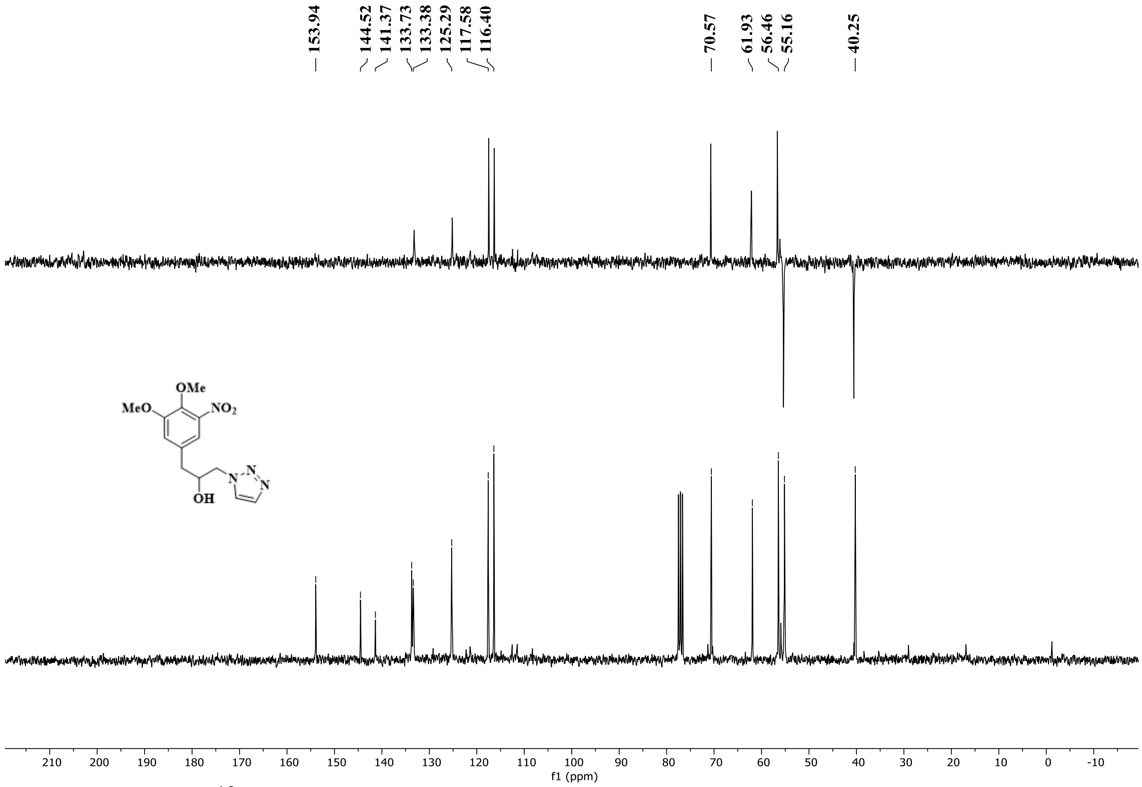

Figure 105: ^{13}C NMR spectrum and DEPT-135 of compound **33** ($CDCl_3$, 75Hz)

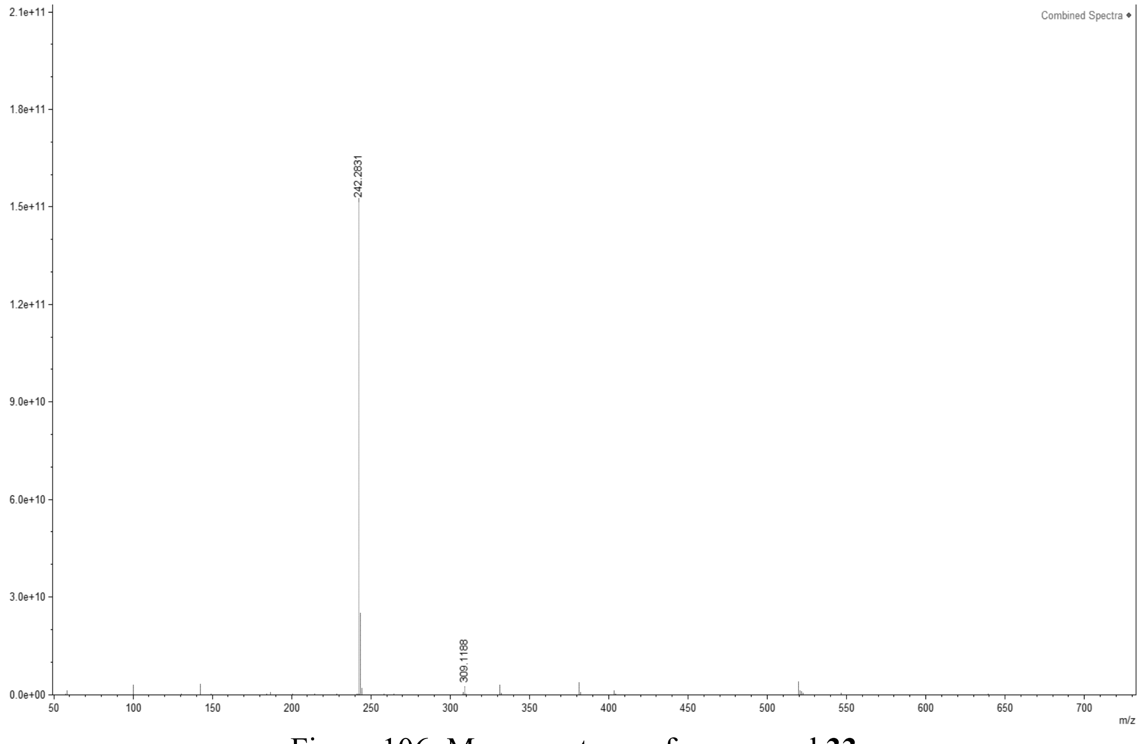

Figure 106: Mass spectrum of compound **33**

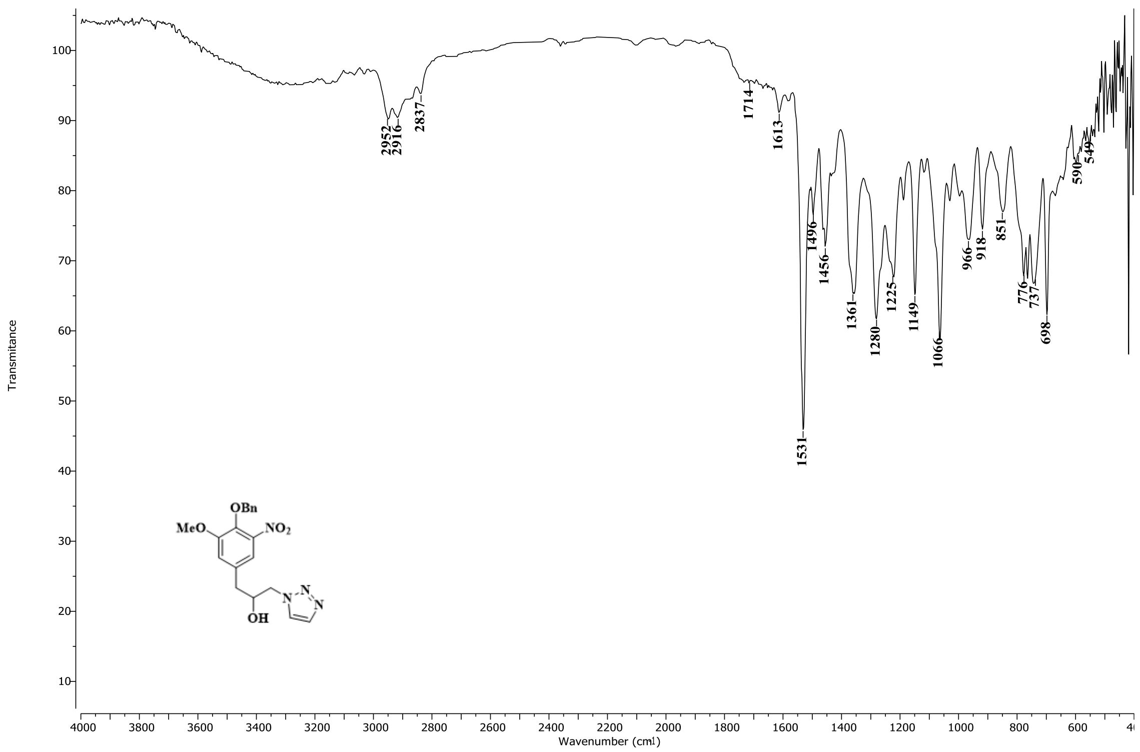

Figure 107: IR spectrum of compound **34**

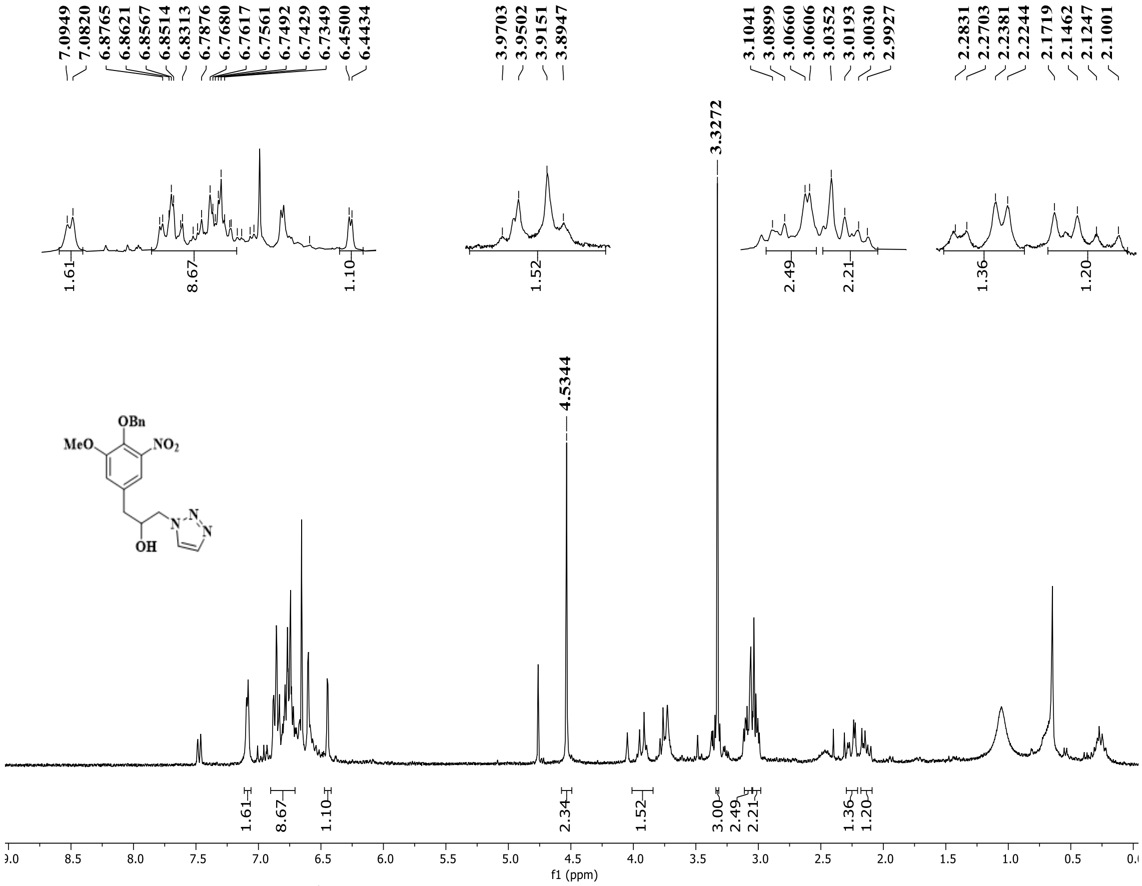

Figure 108: ^{1}H NMR spectrum of compound **34** ($CDCl_3$, 300Hz)

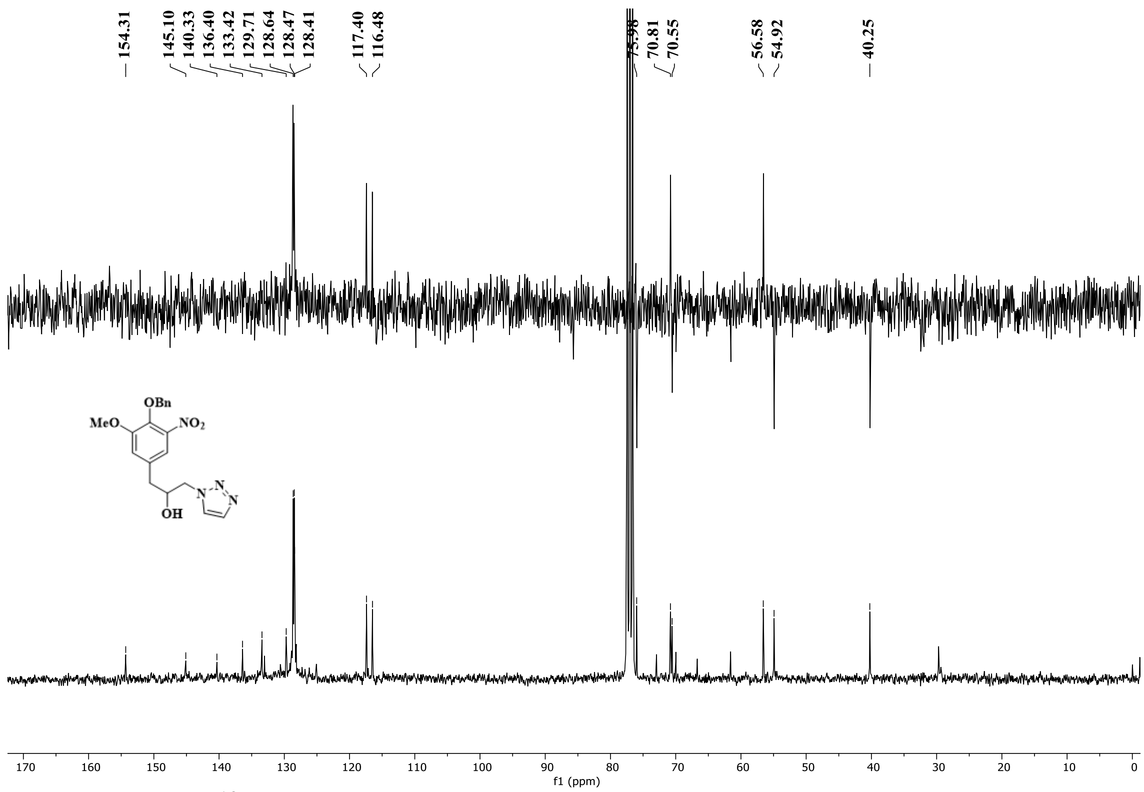

Figure 109: ^{13}C NMR spectrum and DEPT-135 of compound **34** ($CDCl_3$, 75Hz)

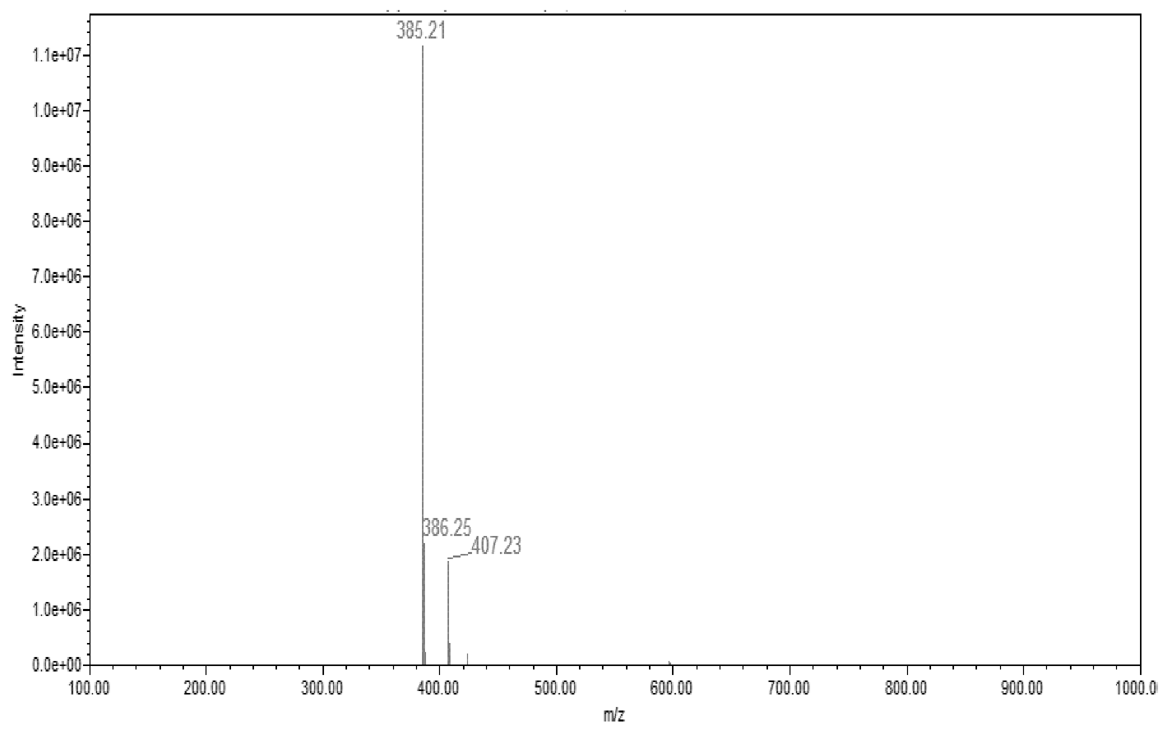

Figure 110: Mass spectrum of compound **34**
